# Online structure-based screening of purchasable approved drugs and natural compounds: retrospective examples of drug repositioning on cancer targets

**DOI:** 10.18632/oncotarget.25966

**Published:** 2018-08-17

**Authors:** Nathalie Lagarde, Julien Rey, Aram Gyulkhandanyan, Pierre Tufféry, Maria A. Miteva, Bruno O. Villoutreix

**Affiliations:** ^1^ Université Paris Diderot, Sorbonne Paris Cité, Molécules Thérapeutiques In Silico, INSERM UMR-S 973, Paris, France; ^2^ INSERM, U973, Paris, France

**Keywords:** virtual screening, docking, purchasable approved drugs, repositioning, cancer

## Abstract

Drug discovery is a long and difficult process that benefits from the integration of virtual screening methods in experimental screening campaigns such as to generate testable hypotheses, accelerate and/or reduce the cost of drug development. Current drug attrition rate is still a major issue in all therapeutic areas and especially in the field of cancer. Drug repositioning as well as the screening of natural compounds constitute promising approaches to accelerate and improve the success rate of drug discovery. We developed three compounds libraries of purchasable compounds: Drugs-lib, FOOD-lib and NP-lib that contain approved drugs, food constituents and natural products, respectively, that are optimized for structure-based virtual screening studies. The three compounds libraries are implemented in the MTiOpenScreen web server that allows users to perform structure-based virtual screening computations on their selected protein targets. The server outputs a list of 1,500 molecules with predicted binding scores that can then be processed further by the users and purchased for experimental validation. To illustrate the potential of our service for drug repositioning endeavours, we selected five recently published drugs that have been repositioned *in vitro* and/or *in vivo* on cancer targets. For each drug, we used the MTiOpenScreen service to screen the Drugs-lib collection against the corresponding anti-cancer target and we show that our protocol is able to rank these drugs within the top ranked compounds. This web server should assist the discovery of promising molecules that could benefit patients, with faster development times, and reduced costs and risk.

## INTRODUCTION

Virtual screening methods are nowadays fully integrated in the drug discovery pipelines and combined with high throughput screening to accelerate and reduce the cost of drug development [1–4]. Among the different virtual screening approaches, docking methods can be used to probe the binding pocket of a potential target and rationally select and reduce the number of compounds that should be experimentally tested. The approach is of interest as the availability of three-dimensional structures (experimental and modeled) of proteins (including membrane proteins) and nucleic acids is increasing significantly each year. However, despite the use of various modern high-throughput technologies, the drug discovery process requires many years of research and development and the failure rate in clinical trials remains very high, close to 90% [5], essentially because of the lack of efficacy and safety of the drug candidates [6]. The drug attrition rate is particularly high in the cancer area with a failure rate in clinical trials around 95% [7]. In order to speed-up the discovery of new treatments, drug repositioning that aims to find new uses for existing drugs is considered as an effective and alternative paradigm in drug development. Indeed, existing drugs are well-understood ingredients that regulatory agencies have approved for human use in the context of diseases and as such have in general well characterized pharmacokinetic behaviours with known safety issues if any [8]. Another possibility to identify novel drugs is to learn from natural products. Here the process is obviously longer than by using drug repositioning approaches, but natural products present the advantages of being in general chemically dissimilar from the synthetic compounds which enable to search for hits in a larger chemical space with often enhanced bioavailability potency or that act via original molecular mechanisms evolved over millions of years. For example, natural compounds constitute promising candidates for target considered as difficult in drug discovery such as protein-protein interactions and antimicrobial targets. [9]. Integration in the drug discovery process of virtual screening strategies making use of libraries containing approved drugs [10] and natural compounds [11] represent a very attractive approach in all therapeutic areas and definitively in the field of cancer.

Very few automatic and open access structure-based virtual screening tools are available to conduct drug repositioning studies. Several webservers are available for target fishing [12], i.e. to try to identify a protein target for a given compound, and thus can be applied to drug repositioning [13–15]. Yet, to the best of our knowledge, the only webserver that provides a prepared virtual library of approved drugs (1852 molecules approved by the FDA between 1939 and 2017) and a facility to screen these compounds over the users' selected protein target is the e-Drugs3D webserver [16]. Our aim here was to provide to researchers interested in drug discovery, regardless of their scientific backgrounds (biologists, chemists, clinicians, computer scientists…), a user-friendly structure-based virtual screening protocol combined with prepared and purchasable approved drug and natural compound collections. We indeed decided to focus here only on purchasable molecules such as to facilitate the discovery process. We thus constructed a compound collection named Drugs-lib, by compiling and filtering four databases of approved drugs to produce a final library that includes only purchasable approved drugs presenting structures suitable for docking computations. We proceeded the same way to obtain two other electronic libraries of natural compounds, one is named FOOD-lib that includes food constituents and the NP-lib dedicated to natural products. These three libraries are implemented in our free structure-based virtual screening webservice, MTiOpenScreen. Here, we present the construction and implementation of these three libraries and the potential use of the Drugs-lib to identify new anti-cancer indications for existing drugs using five retrospective examples of approved drugs successfully repositioned experimentally, *in vitro* and *in vivo*, onto oncogenic targets [17–22].

## RESULTS

### Purchasable approved drugs, food and natural compounds libraries

**Figure 1:**
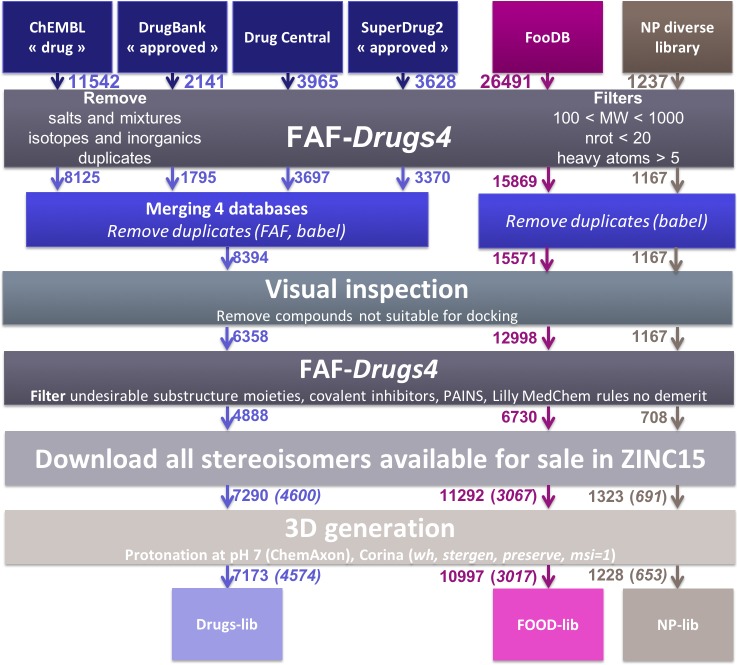
Schematic representation of the protocol used to generate the Drugs-lib, FOOD-lib and NP-lib The number located close to the arrow indicates the number of compounds remaining in the database at each step. After the Download all stereoisomers available for sale in ZINC15 step, the number in brackets corresponds to the number of compounds when considering only one isomer in the library.

**Figure 2:**
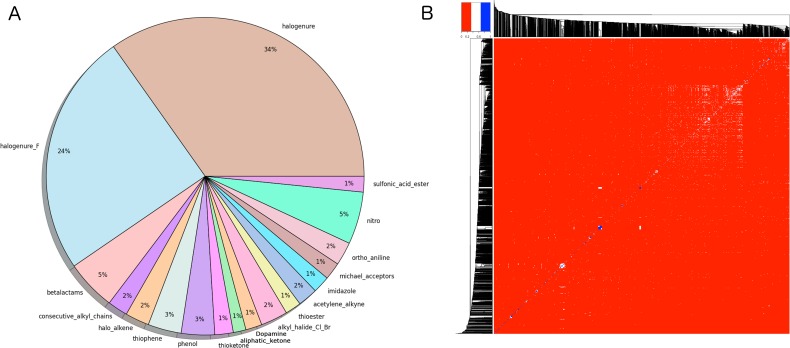
**(A)** Graphical representation of the problematic moieties identified using the of FAF-Drugs4 toxicophore-like filters for the Drugs-lib. **(B)** Heatmap representation of (1 - distance matrix) for the Drugs-lib. The distance matrix was computed using atom pair fingerprints and the dendogram was computed using PubChem's fingerprints. The closer the regions of the grid are to the color red, the less similar the pair of ligands.

Compounds libraries generation. Drugs-lib, FOOD-lib and NP-lib are compounds libraries that contain approved drugs, food constituents and natural products, respectively, optimized for structure-based virtual screening studies. The three compounds libraries are implemented in the MTiOpenScreen web server [23] that allows user to perform structure-based virtual screening computations on a selected protein target. The three compounds libraries were built using the same protocol (see Methods section and Figure 1) that includes the use of FAF*-Drugs4* [24] physico-chemical and toxicophore filtering (Figures 2A and Supplementary Figure 1), a visual inspection step to remove compounds not suitable for docking (e.g., compounds with long aliphatic flexible side chains, compounds with very complex structures, …) and the assessment of their purchasability according to the ZINC15 database [25]. The Drugs-lib was constructed by using as a starting point four databases of approved drugs, the “drug” subset of the ChEMBL database [26], the “approved” subset of DrugBank [27] version 5.0.10, the DrugCentral online compendium [28] and the “approved” SuperDrug2 database version 2.0 [29]. A total of 21,276 compounds were initially included in the Drugs-lib construction process, but only 7173 stereoisomers corresponding to 4574 single isomer drugs were kept after removal of the duplicates and the filtering protocol mentioned in the method section (Table 1). The 26,941 food constituents of the FooDB (http://www.foodb.ca/) with available smiles strings were used as input for the FOOD-lib generation. After removal of the duplicates and the filtering steps, the FOOD-lib included 10997 stereoisomers corresponding to 3015 single isomer food constituents (Table 1). A purchasable diverse library of natural products of 1237 compounds [30] was processed to construct the NP-lib that gathers, after the filtering steps, 1228 stereoisomers corresponding to 653 single isomer natural products (Table 1).

**Table 1:** Number of compounds (#cmpds), number of unique compounds (#unique cpds) and mean values (in bold) of the six descriptors computed with FAF-*Drugs4* for the whole Drugs-lib (7173 compounds), the whole FOOD-lib (10997 compounds) and the whole NP-lib (1228 compounds): molecular weight (MW), octanol-water partition coefficient (logP), number of hydrogen bond donors (HBA) and acceptors (HBD), topological polar surface area (tPSA), and number of rotatable bonds (nrotB). The minimum and maximum values for each property are in italic, standard deviation values are indicated in brackets, the 95%-confidence interval is noted in square brackets

	#cmpds	#unique cmpds	MW	logP	HBD	HBA	tPSA	nrotB
Drugs-lib	7173	4574	**357.464** *128.22 – 974.61* (113.121) [354.84;360.09]	**2.656** *-10.59 – 12.75* (2.261) [2.60;2.71]	**1.914** *0 - 20* (1.920) [1.87;1.95]	**5.571** *0 - 23* (3.030) [5.49;5.63]	**79.954** *0 – 414.34* (49.050) [78.64;80.91]	**5.506** *0 - 20* (4.650) [5.56;5.77]
FOOD-lib	10997	3015	**346.798** *185.07 – 796.98* (103.851) [344.85;348.75]	**2.429** *-10.17 – 11.08* (3.535) [2.36;2.50]	**2.944** *0 - 12* (2.246) [2.90;2.99]	**5.500** *0 - 21* (3.546) [5.43;5.57]	**93.964** *0 – 379.05* (61.949) [92.77;95.10]	**4.280** *0 - 20* (3.438) [4.24;4.37]
NP-lib	1228	653	**388.044** *131.17 – 961.14* (165.429) [378.78;397.31]	**2.870** *-11.03 – 13.54* (2.902) [2.71;3.03]	**2.926** *0 - 17* (2.884) [2.76;3.09]	**6.113** *0 - 28* (4.306) [5.87;6.35]	**96.424** *0 – 445.44* (70.021) [92.50;100.34]	**4.354** *0 - 20* (3.507) [4.35;4.55]

**Figure 3:**
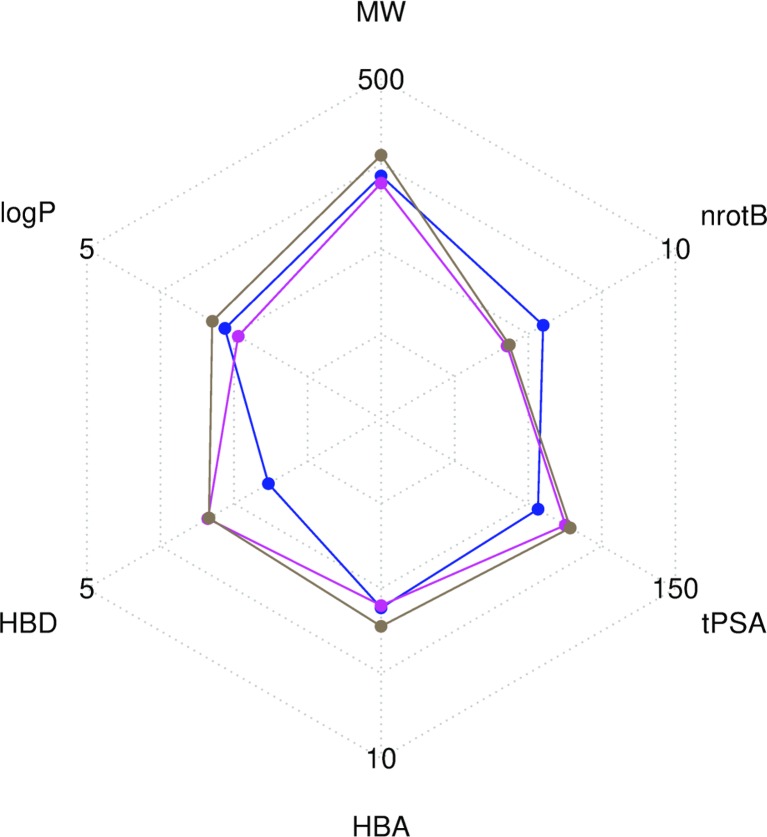
Radar plot representation of the mean values of six descriptors computed with FAF-*Drugs4* The Drugs-lib is shown in blue, the FOOD-lib in magenta and the NP-lib in brown. The plot involves the following molecular descriptors: molecular weight (MW), number of rotatable bonds (nrotB), topological polar surface area (tPSA), number of hydrogen bond acceptors (HBA) and donors (HBD) and octanol-water partition coefficient (logP).

Compounds libraries diversity. For each compound, all stereoisomers tagged as purchasable in the ZINC15 database were included in the corresponding compounds library. Up to 38, 42 and 12 stereoisomers per unique compound are counted in respectively the Drugs-lib, the FOOD-lib and the NP-lib. The average number of stereoisomers per molecule (Supplementary Figure 2) is of 1.6 for the Drugs-lib (median value equal to 1), 3.6 for the FOOD-lib (median value equal to 2), and 1.9 and for the NP-lib (median value equal to 1). The structural diversity of the compounds included in each data set was investigated using atom pair fingerprints (Figures 2B Supplementary Figure 3). To visualize the chemical space covered by each library, we used principal component analysis and the FragFp descriptors (binary fingerprint that relies on a dictionary of 512 predefined structure fragments) computed in DataWarrior [31] (Supplementary Figure 4). The values of six important physico-chemical properties were computed with our FAF*-Drugs4* server and the mean values for the different libraries are shown in Figure 3 and Table 1.

### Retrospective examples of the use of the Drugs-lib for drug repositioning on cancer targets

The Drugs-lib was developed for structure-based virtual screening and conceived to be implemented within the MTiOpenScreen service such that if users have an experimental 3D structure of a target or a model built by homology, they could initiate a drug repositioning study. Indeed, as output, the server will provide a list of compounds that could be purchased and tested experimentally.

In this study, we selected five published examples of drugs that have been repositioned on cancer targets. For each drug, we used the MTiOS server to screen the Drugs-lib against the corresponding anti-cancer target and we evaluated the ability of our virtual screening protocol to identify the known repositioned drug within the top 1500 scores.

#### Fluspirilene

**Figure 4:**
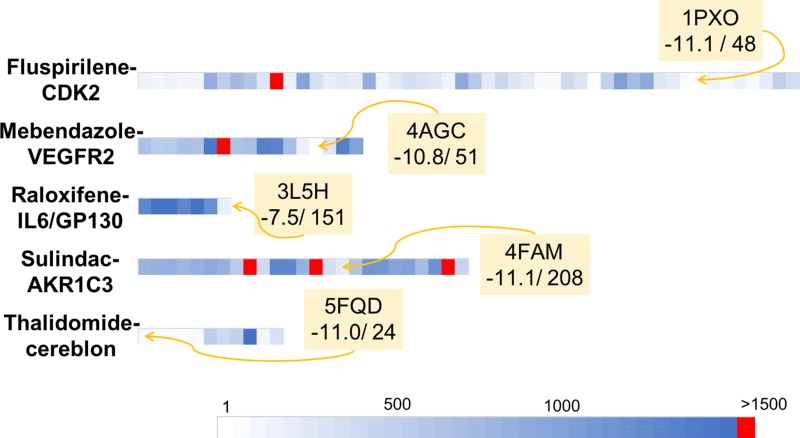
Graphical representation of the retrospective virtual screening results for each example of repositioned drug - oncogenic target system: fluspirilene - CDK2, mebendazole – VEGFR2, raloxifene – IL6/GP130, sulindac – AKR1C3 and thalidomide – cereblon (each system is represented by one line) For each line, each coloured square represents the rank obtained for the studied repositioned drug (i.e. fluspirilene, mebendazole, raloxifene, sulindac, thalidomide for the first, second, third, fourth and fifth lines respectively) in a given PDB structures. The gradient of colours scale from white for structures for which the repositioned drug was ranked in the first positions to blue for structures for which the repositioned drug was ranked close to the position 1500. Red square represents structure for which the repositioned drug was not identified as a hit (i.e. ranked over the 1500 position).

Fluspirilene [32] is an antipsychotic drug, administered by intramuscular injection, used for the treatment of schizophrenia. Fluspirilene mechanism of action is thought to be mediated by inhibition of the dopamine D2 receptor [33] and blockade of a calcium channel [34]. In 2015, Shi et al. [35] used *in silico* screening computations carried out over 4914 FDA-approved small molecule drugs to identify the cyclin-dependent kinase 2 (CDK2) as a new target for fluspirilene. Both *in vitro* and *in vivo* experiments confirmed the potential of fluspirilene as a new anti-cancer drug for the treatment of hepatocellular carcinoma. CDK2 is a member of the cyclin-dependent kinases family that is involved in cell replication and tumor growth, and is known to be a promising therapeutic target for cancer therapy [36]. Numerous CDK2 inhibitors have already been reported in the literature, and the CDK2 binding site is well-characterized. In the Protein Data Bank (PDB), 358 human CDK2 structures co-crystallized with a ligand (holo structures) are available. We selected the 4EK4 and 4KFL PDB structures to perform the virtual screening computations, as they are holo PDB structures, i.e. co-crystallized with a ligand, that present the best resolution. In the 4EK4 and 4KFL MTiOpenScreen service outcomes, fluspirilene was ranked respectively at position 186 and 886 with predicted AutoDock Vina scores equal to −9.3 and −8.8 kcal/mol respectively (Supplementary Table 1). We repeated four times the screening protocol using the 4EK4 structure to ensure the repeatability and the reliability of the results (Supplementary Table 1). The AutoDock Vina scores and ranks associated to fluspirilene in these four runs were highly similar to those obtained in the first screening run with 4EK4, with scores varying between −9.4 to −9.3 and ranks ranging from 149 to 190. To reduce the computational time associated with MTiOpenScreen service virtual screening of the Drugs-lib on each of the 358 holo CDK2 PDB structures, we selected a representative dataset of 44 CDK2 proteins (Supplementary Table 1) that was used to identify fluspirilene as a CDK2 inhibitor [35]. The best result was obtained using the 1PXO structure, for which fluspirilene was ranked 48 with a predicted AutoDock vina score of −11.1 (Supplementary Table 1 and Figure 4). For all the other structures, except 1E1V, fluspirilene was ranked in the top 1500 best compounds, with ranks ranging from 83 to 1048 and AutoDock Vina scores between −12.2 to −8.4 according to the PDB structure used (Supplementary Table 1 and Figure 4). To get some insights about the diversity of molecules identified as hits by our virtual screening protocol, we used the 1500 best scored compounds associated with the 4EK4 PDB structure and we computed in DataWarrior [31] the similarity between each pair of compounds according to the FragFp descriptors (Supplementary Figure 5). Three compounds that are clustered together with fluspirilene, i.e. that are very similar to fluspirilene, were also ranked within the 1500 best scored drugs: spirilene, spiperone and fluspiperone.

#### Mebendazole

Mebendazole [37] is an anthelmintic drug that has been used for more than 20 years for the treatment of a variety of parasitic infestations, both in human and veterinary medicine. Mebendazole acts by binding to tubulin [38] and induces tubulin de-polymerization which then disrupts the formation and functions of microtubules [39]. With its roles on tubulin and induction of apoptosis, it was postulated that mebendazole could be used for cancer treatment [40, 41]. Interestingly, it was shown that mebendazole not only inhibits cancer cell growth by induction of apoptosis (and thus by interacting with tubulin) but also seems to have an inhibitory effect on angiogenesis [42]. VEGFR2 (Vascular Endothelial Growth Factor 2), a major regulator of angiogenesis [43] was identified as a new molecular target for mebendazole [44], that could explain its anti-angiogenic potency. Mebendazole ability to bind and inhibit VEGFR2 function was first predicted using the *train, match, fit, streamline* (TMFS) proteo-chemometric method applied on a library of 3671 FDA-approved compounds, and then confirmed experimentally with *in vitro* assays. Another study [19] showed that mebendazole competes with ATP, selectively inhibits tumor angiogenesis but not the normal brain vasculatures and suppresses VEGFR2 kinase *in vivo*. Numerous VEGFR2 inhibitors have been described in the scientific literature, and some of them have been co-crystallized with VEGFR2, revealing the VEGFR2 tyrosine kinase domain binding site. The 2XIR PDB structure, the holo VEGFR2 structure presenting the best resolution (1.5 Å), was selected to screen the Drugs-lib against the 2XIR PDB structure using the MTiOpenScreen service. Mebendazole was ranked at position 713 with a predicted AutoDock Vina score of −9.5 kcal/mol (Supplementary Table 2). The screening protocol was repeated five times using the 2XIR structure to ensure the repeatability and the reliability of the results (Supplementary Table 2). Very similar AutoDock Vina scores and rank were obtained for mebendazole, in the five MTiOpenScreen service runs, with scores varying between −9.6 kcal/mol and −9.5kcal/mol and ranks ranging from 585 to 713. In order to explore additional regions of the receptor conformational space, we extracted from the PDB, 35 other holo VEGR2 crystallographic structures and we repeated the same protocol on the 13 structures with no missing residues in the binding sites (see Methods section) by screening the Drugs-lib against each one of these holo VEGFR2 PDB structures (Supplementary Table 2). The structure associated with the best outcomes for mebendazole was 3U6J with a rank at position 51 and a corresponding AutoDock vina score equal to −10.8 (Supplementary Table 2 and Figure 4). For all other structures except the 3U6J PDB structure, mebendazole was ranked in the top 1500 best compounds, with ranks between positions 205 and 1448 and scores ranging from −10.6 to −8.6 (Supplementary Table 2 and Figure 4). We investigated the diversity of the 1500 best scored drugs associated with the 2XIR PDB structure by using the FragFp descriptors to compute the similarity between each pair of compounds in DataWarrior [31] (Supplementary Figure 6). We noticed that four compounds that are chemically similar to mebendazole, namely flubendazole, luxabendazole, nocodazole and oxfendazole are also identified as hits by our virtual screening protocol.

#### Raloxifene

Raloxifene is a non-hormonal anti-resorptive agent widely prescribed for the prevention and treatment of postmenopausal osteoporosis [45]. Raloxifene acts by binding to estrogen receptor (ER) isoforms ER_alpha and ER_beta and belongs to the selective estrogen receptor modulator (SERM) class [46]. Recently, an *in silico* protocol [47] identified raloxifene and its analogue bazedoxifene as new potential IL-6/GP130 protein-protein interface inhibitors. IL-6 (Interleukin-6) and GP130 are part of the IL-6/JAK/STAT3 pathway that is involved in proliferation, survival, invasiveness and metastasis of tumor cells and in suppression of the anticancer immune response, and constitutes an attractive target for anti-cancer therapies. [48] The *in silico* findings reported by Li et al. were confirmed by *in vitro* experiments, paving the way for the investigation of the potential use of raloxifene and its analogues in therapies for IL-6/GP130/STAT3 dependent cancers. The predicted binding site of raloxifene is located at the surface of the GP130 protein, in the GP130/IL-6 D1 domain interface. In the PDB, three crystal structures of the human GP130 protein including this D1 domain are available (PDB IDs: 1I1R, 1P9M, 3L5H). We used the MTiOpenScreen service to screen the Drugs-lib against the 1P9M structure as it was the only PDB structure of the trimer IL-6/IL-6R/GP130. Raloxifene was ranked 1088 with a predicted AutoDock Vina score of −6.5 kcal/mol (Supplementary Table 3). We repeated the screening protocol five times using the 1P9M structure to ensure the repeatability and the reliability of the results (Supplementary Table 3). Similar raloxifene's AutoDock Vina scores (from −6.4 to −6.5) and ranks (from 1088 to 1269) were obtained. We then reproduced the same protocol using this time the two other PDB structures 1I1R and 3L5H (Supplementary Table 3). The best result was obtained by using the 3L5H PDB structure, where raloxifene was ranked at position 151 with a predicted AutoDock Vina score of −7.5 kcal/mol (Supplementary Table 3 and Figure 4). Using the 1I1R structure, the AutoDock Vina score associated to raloxifene was equal to −6.4 corresponding to rank 1012 (Supplementary Table 3 and Figure 4). The chemical similarity of the compounds identified as hits by screening the Drugs-lib on the 1P9M PDB structure was evaluated using the FragFp descriptors in DataWarrior [31] (Supplementary Figure 7). Within these 1500 compound, only arzoxifene presents a high chemical similarity to raloxifene.

#### Sulindac

Sulindac is a non-steroidal anti-inflammatory drug (NSAID), commonly used to treat pathologies such as osteoarthritis, ankylosing spondylitis, rheumatoid arthritis and gout. Sulindac share the classical NSAID anti-inflammatory mechanism of action, by targeting the COX enzymes (sulindac is a non-selective COX inhibitor) and inhibition of prostaglandins synthesis [49]. Sulindac and other NSAID have been identified as AKR1C3 (Aldo-Keto Reductase 1C3) inhibitors. Interestingly, AKR1C3 is over-expressed in a variety of cancers and its inhibition leads to the conversion of the PGD2 prostaglandin to PGJ2 prostanoids that presents anti-neoplastic properties [50]. Thus, AKR1C3 constitutes a promising anti-cancer target and its inhibitors, among which sulindac, represent potential new leads for anti-cancer drug development. The crystallographic structure of sulindac bound to AKR1C3 is available in the PDB (PDBID: 3R7M), together with 39 other holo crystallographic structures of human AKR1C3. Among these 40 structures, 19 presenting missing binding site residues (including 3R7M) are not directly suitable for the docking and weren't considered during the protein structure selection process. We selected the 4WDT PDB structure that presents the best resolution (1.5 Å), among the 21 remaining PDB structures, to screen the Drugs-lib using the MTiOpenScreen service. Sulindac was ranked 817 with a corresponding predicted AutoDock Vina score was equal to −10.1 (Supplementary Table 4). To evaluate the repeatability of the results, we proceeded with four additional runs of the screening protocol using the 4WDT structure (Supplementary Table 4). The same AutoDock Vina score of −10.1 kcal/mol was obtained in the four supplementary runs, with very similar corresponding ranks in the range 778 to 858. We then investigated using the 20 remaining complete holo AKR1C3 PDB structures whether using a different PDB structure would lead to similar results. We applied the same virtual screening protocol and we screened the Drugs-lib against each holo AKR1C3 PDB structure using the MTiOpenScreen service (Supplementary Table 4). For 17 structures among 20, sulindac was ranked in the top 1500 best compounds, the best rank being associated with the 1S2A PDB structure (rank 88) (Supplementary Table 4 and Figure 4). The corresponding predicted AutoDock Vina scores ranged from −11.6 (5JM5) to −9.2 (4DBU). We used the crystallographic structure of the complex AKR1C3/Sulindac (PDBID: 3R7M) to evaluate the accuracy of the AutoDock Vina predicted binding modes for sulindac (Supplementary Table 4) using the Root Mean Square Deviation (RMSD) metric. The MTiOpenScreen service predicted binding modes for sulindac to AKR1C3 were not similar to the experimental one (with RMSD values ranging from 3.741 to 7.310, Supplementary Figure 8), yet, in several cases, the ligand still had several similar non-covalent contacts as found in the experimental structure. To investigate the chemical diversity of 1500 best scored compounds associated with the 4WDT PDB structure, the similarity between each pair of compounds according to the FragFp descriptors was computed in DataWarrior [31] (Supplementary Figure 9). Three compounds that are very similar to sulindac were also ranked within the 1500 best scored drugs: exisulind and two other sulindac stereoisomers.

### Thalidomide

Thalidomide is one of the most famous examples of drug repositioning. The history of thalidomide started in 1956 when it was first marketed in Germany as a sedative also used to prevent morning sickness in pregnancy. However, thalidomide was withdrawn from the worldwide market in 1963, after the discovery of the severe teratogenic effects presented by the babies exposed to thalidomide *in utero* (between the 34^th^ and 50^th^ day of pregnancy) [51]. Surprisingly, this compound, that will ever be associated with one of the worst pharmaceutical disaster, has become the object of major interest after demonstration of its wide range of biological effects, notably its immunomodulatory and anti-angiogenic effects. Thalidomide has been successfully used for the treatment of multiple myeloma [52], its beneficial effect resulting from the binding and the activation of cereblon [53], that is part of E3 ubiquitin ligase complex. This activation leads to the down-regulation of two transcription factors involved in B cell development (IKZF1 and IKZF3), highly expressed in multiple myeloma. The binding site of thalidomide on cereblon was experimentally resolved and seven holo X-Ray structures of the human cereblon protein are available at the PDB (Supplementary Table 5). We chose the 5FQD PDB structure because of its lower resolution (2.45 Å) and we used it to screen the Drugs-lib with the MTiOpenScreen service. Thalidomide was ranked 24 with a predicted AutoDock Vina score of −11.0 kcal/mol (Supplementary Table 5). We repeated 5 times the same screening procedure on the 5FQD structure to assess the repeatability of the results. Highly similar ranks for thalidomide were obtained within the different runs (varying from rank 24 to rank 27) and the associated AutoDock Vina scores were ranging from −11.0 kcal/mol to −10.9 kcal/mol (Supplementary Table 5). We then evaluated the performance of our screening protocol using a different cereblon PDB structure as input. We screened the Drugs-lib against each one of the six additional holo cereblon structure available. We obtained results very similar to the 5FQD outcomes (Supplementary Table 5, Figure 4). The best result was obtained with the 5HXB PDB structure for which the thalidomide was ranked in position 53 with a predicted AutoDock vina score of −10.5. For all other structures, thalidomide was always ranked in the 1500 best compounds with ranks varying between position 138 and 637 and the scores ranged from −9.9 to −9.1. We took advantage of the crystallographic structure of the complex thalidomide/cereblon (PDBID: 4CI1) to evaluate the accuracy of the AutoDock Vina predicted binding modes for thalidomide (Supplementary Table 5, Supplementary Figure 10) as measured by the RMSD metric. For all cereblon PDB structures, the MTiOpenScreen service predicted binding modes for thalidomide to cereblon were fitting the experimental one (with RMSD values ranging from 0.349 to 1.133). It is interesting to note that for the 4TZ4 structure, the first pose was not similar to the thalidomide experimental binding mode (RMSD of 6.179) but using the second pose (with also a very favorable score of −8.8) we could recover the experimental binding mode of thalidomide to cereblon (RMSD of 0.711). The chemical similarity of the hits compounds when using the 5FQF PDB structure was evaluated using the FragFp descriptors implemented in DataWarrior [31] (Supplementary Figure 11). Additionally to the second thalidomide stereoisomer, the thalidomide analog lenalidomide was also ranked in the 1500 best scored compounds.

**Figure 5:**
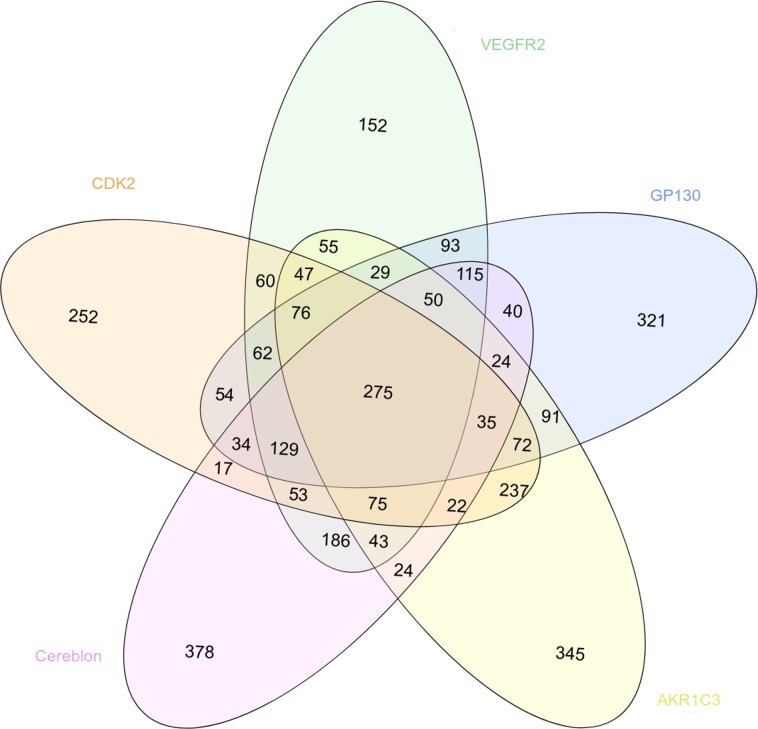
Venn diagram showing the overlapping between the 1500 best scored drugs resulting from the virtual screening with MTiOpenScreen of the Drugs-lib against the 5 cancer targets CDK2 (in light orange, PDB structure 4EK4), VEGFR2 (in green, PDB structure 2XIR), GP130 (in blue, PDB structure 1P9M), AKR1C3 (in yellow, PDB structure 4WDT) and cereblon (in pink, PDB structure 5FQD)

We also evaluated the specificity of the virtual screening results obtained with our 5 cancer targets by comparing the list of the 1500 best scored compounds obtained with the structure presenting the best resolution for each target (Figure 5). According to the considered system, between 152 and 378 compounds were specific for one target, i.e. predicted as a hit for one target only. In the opposite, 275 compounds were identified as hits for the 5 systems.

## DISCUSSION

Virtual screening methods are commonly used in drug discovery to rationally narrow the number of compounds that needs to be tested experimentally [1, 3]. The choice of the initial virtual library of compounds is a critical step to ensure the success of the whole protocol. In particular, to overcome the actual attrition rate of new drugs approval, which is particularly dramatic in the cancer area, interest has emerged towards exploiting already approved drugs [10] and natural products [11]. We thus decided to construct a virtual library of purchasable approved drugs and two virtual libraries of purchasable natural compounds (natural products and food constituents) that were then implemented in the online structure-based virtual screening web service MTiOpenScreen service.

### Purchasable approved drugs, food and natural compounds libraries

Compounds libraries generation. The three virtual libraries of compounds were constructed by gathering and/or filtering available databases of approved drugs and natural products. To construct the Drugs-lib, four databases of approved drugs were used as initial sets of compounds: the “drug” subset of the ChEMBL database [26], the “approved” subset of DrugBank [27] version 5.0.10, the DrugCentral online compendium [28] and the “approved” SuperDrug2 database version 2.0 [29]. We assumed that selecting these four databases should ensure an exhaustive inclusion of approved drugs in the Drugs-lib. Even if precisely quantify the overlaps among these four databases is impaired by drugs name and stereochemistry variations between the databases, we could obtain an approximation using the babel “remove duplicates” utility (Supplementary Figure 12). Around 3000 compounds are common to all databases and the majority of compounds are found in the ChEMBL ‘drug’ subset, but each of the three remaining databases provides around 100 additional unique compounds. The FOODB-lib is dedicated to food constituent molecules and initially included the compounds of the FooDB (http://www.foodb.ca/) with available smiles strings. Finally, several databases of natural products are available [54], but we chose to use as a starting point for the NP-lib, a purchasable diverse library of natural products of 1237 compounds [30]. The compounds of this diverse library were extracted from the UNPD database [55] and the Dictionary of Natural Products [56] according to a protocol developed to select a small but representative subset of natural compounds. The Drugs-lib, FOOD-lib and NP-lib were constructed to be implemented in the MTiOpenScreen service such as to be used in drug discovery structure-based virtual protocols. As a consequence, we filtered our initial sets of compounds according to three criteria: (1) the compounds should be suitable for docking computations (which excludes highly flexible compounds and very small or large molecules); (2) the compounds structures should not include major documented toxicophores; and (3) the compounds should be purchasable to enable experimental validation of the docking predictions. Our filtration protocol associates both automatic filtration steps using FAF*-Drugs4* and babel, and a visual inspection step to validate the selection of compounds that should be included in the datasets. Finally, the Drugs-lib includes 7173 stereoisomers corresponding to 4574 single isomer drugs, the FOOD-lib gathers 10997 stereoisomers corresponding to 3015 single isomer food constituents and the NP-lib is formed of 1228 stereoisomers corresponding to 653 single isomer natural products.

Compounds libraries diversity. Structural dissimilarity of compounds, which is used to measure the chemical space covered by a dataset, is one of the key points to ensure success of virtual screening protocols. Among the different approaches that can be used for its measure (see [57] for a review), we chose in this study, to use atom pair fingerprints to measure distances between compounds within each library and FragFp fingerprint descriptors to visualize the chemical space covered by the three libraries. Results show that, despite their relative limited size, the three libraries include compounds that are structurally diverse. The FOOD-lib and NP-lib chemical space coverage are largely overlapping, which is logical since the food constituents listed in the FooDB are chemical compounds present in unprocessed foods and are thus also natural products. Interestingly, even if there is a clear separation between the Drugs-lib chemical space coverage and the FOOD-lib and NP-lib ones, there are some overlapping areas as numerous approved drugs were inspired by natural products or natural product mimics [58]. The predicted physico-chemical property values (MW, logP, nrotB, TPSA, HBD and HBA) of the three final libraries are reported in Table 1 and Figure 3. These further highlight an interesting distribution of properties for drug discovery endeavors.

### Retrospective examples of the use of the Drugs-lib for drug repositioning on cancer targets

We were here interested to apply our screening server with the Drugs-lib on previously reported drug repositioning [59] studies. The aims of this approach are to accelerate and improve the success rate of drug development, which are essential for all therapeutic areas and especially in the field of cancer. Indeed, current development of new anti-cancer drugs suffers of several major difficulties: the process is long and expensive, the success rate in Phase I of clinical trials is low with poor survival benefits, while chemo-resistance and adverse effects are frequently observed [60]. To evaluate the ability of our screening protocol to identify new use for existing drugs in anti-cancer therapies, we searched the literature for examples of drugs that were successfully repositioned as anti-cancer agents. Because we are using a structure-based virtual screening approach, we focused on cases for which (1) the molecular target responsible for the anti-cancer properties of the drug was identified and (2) an experimental structure of the molecular target was available in the PDB. We selected five drug-protein target test cases to realize this retrospective validation: fluspirilene-CDK2, mebendazole-VEGFR2, raloxifene-GP130, sulindac-AKR1C3 and thalidomide-cereblon. For the five systems, several PDB structures were available for the target proteins. The protein being considered as rigid during the docking computations, the choice of the starting protein structure is one of the key points that could seal the success or failure of the virtual screening protocol [61–66]. In this study, we selected the holo PDB structures (i.e. co-crystallized with a ligand) that had the best resolution with no missing residues in the binding site areas. It is to note that for GP130, only apo structures (i.e. structures in unbound conformations) were available and then were used for this study. For the other oncotargets presented here, suitable apo structures were available for CDK2 (PDB ID: 5IF1) and cereblon (PDB ID: 6BN8) and the screening of the Drugs-lib using these structures led to results comparable to those obtained with the holo structures (data not shown). Yet, it is in general recommended to use holo structures as starting point for virtual screening. We are aware that the resolution is not the perfect metric to guide protein structure selection for virtual screening, but it presents the advantage of being directly available in the PDB file and can be easily followed by researchers that are not familiar with structure-based methods and protein structure handling. More experienced users could also investigate the binding pocket and select the conformations that are more open. Yet, using our automatic virtual screening server and the structures presenting the best resolutions, we successfully identified, for each test case, the corresponding repositioned drug ranked within the first 1500 best scores. The results obtained could be reproduced since repeating the docking protocols several times for each system led to highly similar or identical outcomes in terms of docking scores and ranks. Despite some conformational flexibility observed in the binding sites (Supplementary Figure 13), the results were also reproducible with different PDB structures of the same protein. Indeed, regardless the structure used, the repositioned drug was always retrieved in the 1500 best compounds, except for one CDK2 PDB structure among 44 coordinate files (1E1V), one VEGFR2 structure among 13 (3U6J) and three AKR1C3 structures among 21 coordinate files (1SC, 4DBU and 5HNT). Using the 1E1V PDB structure, fluspirilene was ranked 1526 with a predicted AutoDock vina score equal to −8.5. Fluspirilene is very close to be in the top 1500 list and the score of −8.5 is shared by compounds ranked from positions 1451 to 1668. The docking of mebendazole to the 3U6J structure was associated with a predicted AutoDock Vina score of −9.1 and the corresponding rank was 1793. The comparison of 3U6J binding site residues conformations with the other 12 VEGFR2 PDB structures used in this study (Supplementary Figure 14) shows that the GLU885 in the 3U6J PDB structure present a conformation quite different from the other VEGFR2 structures. This could explain why using the 3U6J PDB structure, mebendazole was not ranked in the first 1500 best compounds. Sulindac predicted binding mode in the 4DBU AKR1C3 PDB structure obtained an AutoDock Vina score of −9.2 and was ranked 2254. AKR1C3 represents a very challenging target for docking computations, with a large binding site with many different subpockets, and a high flexibility of binding site residues (as illustrated in Supplementary Figure 13D) and of entire loop regions [67]. This may explain both the failure of our protocol with the 1S2C, 4DBU and 5HNT structures and its inability to reproduce the crystallized sulindac pose (observed in the 3R7M PDB structure) using other AKR1C3 PDB structures. This suggests that for such proteins, additional work would be required to prepare a coordinate input file for docking purposes.

When several PDB structures are available for docking and when there is no obvious reason to choose one structure over the others, the “ensemble docking” approach (i.e. realize the docking of the database into multiple and diverse conformations of the receptor) can be used. The set of receptor conformations can be obtained by selecting different PDB structures [68] or generated using molecular dynamics simulations and normal mode analysis [69]. The results obtained with such approaches can thus be interpreted not only by considering the scores and ranks of the compounds in each structure of the considered target but by selecting the best AutoDock Vina score in the ensemble of PDB structures for each compound. Using the ensemble docking approach that makes use of several PDB files, the final ranks of fluspirilene, mebendazole, raloxifene, sulindac and thalidomide were respectively 636, 409, 300, 1164 and 53 (and the corresponding AutoDock Vina scores were respectively −12.2, −10.8, −7.5, 11.4 and −11.0) and in all situations the drugs were identified as hits in our output lists.

Focusing on the chemical diversity of the compounds that are ranked within the 1500 best scored drugs, we showed that, for each target, some compounds that are chemically very similar to the query repositioned drug and more dissimilar compounds were predicted as hits. On one side, experimentally testing different analogs of a same chemical series presents the advantages to possibly identify one compound that will present an enhanced activity compared to the other analogs and to give some insights about structure-activity relationships within the series. On the other side, investigating compounds that present quite diverse chemical structures is also very interesting to identify the most potent compounds and the most promising chemical series. Moreover, for each target, between 10 and 25% of compounds were specifically identified as a hit for one target only. It is to note that overlapping between the predicted hits for different targets is expected since we are selecting 1500 compounds over 7173 but the predicted AutoDock Vina scores and ranks should be different between the targets and can also be considered by the user. For example, the glicetanile was ranked in the position 175 on the cereblon target, but only 542 on AKR1C3 and after the 1000^th^ position for CDK2, VEGFR2 and GP130.

Our server should be beneficial to reduce the experimental cost of drug repositioning. Indeed, assuming no additional post-processing investigations of the docked poses provided by the server and testing experimentally 1500 compounds instead of the entire collection enable time- and money-saving. To compare the cost of buying 1500 compounds of the Drugs-lib instead of the whole library, we used information provided at the Sigma Aldrich website (https://www.sigmaaldrich.com/) to make an estimation of the cost of some compounds present in Drugs-lib. This information was directly available for 598 compounds (Supplementary Figure 15) and we hypothesized that these molecules were representative of the whole Drugs-lib. The median price value was $1.32 per mg of compound. Thus, buying 5 mg for the top 1500 compounds selected *in silico* would approximately cost ∼$10,000 versus ∼$48,000 for the whole Drugs-lib collection. Similar costs could be envisioned by investigating the website of other vendors. It is also important to note here that we assume that users have a difficult target with a complex assay that requires a relatively high amount of compounds while in numerous cases, initial biochemical screening experiments can be done with 1 mg of compound, thus lowering significantly the costs of one screening campaign as compared to the one that we mention here. When considering only one occurrence of each drug name in the server output list (ie., as we have several stereoisomers, the same compound name can appear several times in the list but the users can decide to only purchase the main bioactive form, if known, from a chemical vendor), we note that the threshold can be lowered to the top 1000 best compounds. In this case, the associated cost for 5mg for 1000 molecules will be around ∼$7,000 versus ∼$30,000 for the 4574 single isomer drugs of the whole Drugs-lib.

We suggest that using MTiOpenScreen service and the Drugs-lib collection on cancer targets represent a promising and affordable approach to identify new anti-cancer properties of already approved and marketed drugs.

## MATERIALS AND METHODS

### Libraries generation

#### Ligand collection

The compounds used to generate the three datasets, Drugs-lib, Food-lib and NP-lib, were extracted from six different freely available databases. To construct the Drugs-lib, four different drug databases were merged: (1) the “drug” subset of the ChEMBL database [26] included 11.542 compounds (chemicals and biologics), (2) the “approved” subset of DrugBank [27] version 5.0.10 gathering 2141 compounds, (3) the DrugCentral online compendium [28] that contained 3965 compounds and (4) the “approved” SuperDrug2 database version 2.0 [29] that contained 3628 molecules. The Food-lib was constructed using as a starting point the FooDB (http://www.foodb.ca/) a large database on food constituents. The NP-lib was obtained by filtering a purchasable diverse library of natural products [30].

#### Library curation for structure-based virtual screening computations

The three libraries were prepared using the same protocol (Figure 1), with only one additional step of databases merging and duplicates removing to prepare Drugs-lib. The ChEMBL “drug” (https://www.ebi.ac.uk/chembl/drugstore, downloaded 28/11/2017), DrugBank “approved” (https://www.drugbank.ca/releases/latest, downloaded the 14/11/21017) and SuperDrug2 “approved” (http://cheminfo.charite.de/superdrug2/downloads.html, downloaded 13/12/2017) subsets were downloaded in SDF format from their respective website. The DrugCentral subset was downloaded in smiles format (http://drugcentral.org/download, downloaded 04/01/2018). The FooDB database was downloaded (http://foodb.ca/downloads, downloaded 04/01/2018) in sql format and all compounds for which a smiles string was provided were extracted in smiles format. The DrugCentral and FooDB subsets were converted from smiles format to SDF format using Babel [70]. The MolPort IDs of the diverse library of natural products were retrieved from the supplementary data of ref [30] and the corresponding compounds were downloaded in SDF format from the MolPort website (https://www.molport.com/shop/index). Some MolPort compounds with outlier structures were removed. For each of the six initial compound datasets, we used FAF-Drugs4 (Free ADME-Tox Filtering) [24] to perform a primary filter that involved the removal of salts, mixtures, inorganics, isotopes and duplicates. Furthermore, we applied soft physico-chemical filters to remove compounds with a molecular weight inferior to 100 or superior to 1000, more than 20 rotatable bonds and less than 5 heavy atoms. After this first step, and for the Drugs-lib only, we merged the FAF-Drugs4 “Accepted” compounds of the ChEMBL “drug”, DrugBank “approved”, DrugCentral and SuperDrug2 “approved” into a unique dataset. The duplicates compounds were removed using both the Babel --unique /nostereo/nochg option (version 2.3.2, for more information see http://openbabel.org/wiki/Main_Page) and FAF-Drugs4 duplicate tool that compares the canonical smiles of the molecules. This collection containing unique drugs was then used as input for the next library preparation steps. A second filtering step of the three compound collections was achieved by visual inspection of their 2D structures in DataWarrior [31] such as to remove molecules with structures not suitable for docking studies (e.g., drugs with long aliphatic flexible chains, some sugars, some sub-structures that could not be generated in 3D nor docked with the currently available approaches…). We then applied another filtering step to remove compounds containing toxicophores. The computations were performed with several FAF-Drugs4 filtering subroutines: undesirable substructure moieties, retrieve covalent inhibitors, PAINS A, B and C. In FAF-Drugs4, a SMARTS substructure search is used to identity substructures known to be potentially involved in toxicity problems (undesirable substructure moieties), to form covalent bonds with the macromolecular target (retrieve covalent inhibitors) and to belong to the PAINS (Pan Assay Interference Compounds) category of molecules (the PAINS A, B and C filters were used) [71]. For some substructures, a pre-defined cutoff of occurrences is used to decide whether a molecule should be filtered out or not. The output of FAF4-Drugs classifies the input molecules in 3 categories “Accepted” (i.e. compounds with no structural alerts), “Intermediate” (i.e. compounds with low-risk structural alerts and/or for which the number of occurrences are below the defined threshold) and “Rejected” (i.e. compounds that include a high-risk structural alert and/or exceed the threshold of occurrence of low-risk structural alerts) (for more information, see http://fafdrugs4.mti.univ-paris-diderot.fr/groups.html). The FAF-Drugs4 “Accepted” and “Intermediate” compounds were downloaded in SDF format and converted in smiles format using Babel. To investigate whether or not the selected compounds could be purchased, we searched the ZINC15 database [25] with our list of smiles as query input. The compounds tagged as ‘for-sale’, i.e. in-stock compounds (compounds purchased from manufacturers and ready for sale), on-demand compounds (all substances that are for sale) and boutique compounds (compounds often made to order), were kept and their corresponding purchasable stereoisomers were downloaded from the ZINC15 database [25] in SDF format. The compounds were then protonated at pH = 7 using the ChemAxon (Marvin Calculator Plugins version 17.23) calculator plugin [72] (option majormicrospecies -H 7). The 3D conformation of each compound was generated with CORINA Classic [73] (option stergen), by preserving the stereocenters that were already defined in the ZINC SDF file (option preserve). For the present study, only one stereoisomer was generated for each compound (option msi=1). The hydrogen atoms were also added during the 3D conformation generation (option wh). Finally, the compounds were converted in pdbqt format. The final list of drug molecules is shown in Supplementary Table 6.

#### MTi OpenScreen

The MTiOpenScreen web service provides users with a blind docking (full surface) service for a small number of molecules or for extensive docking in a user defined binding pocket (this service is called MTiAutoDock and the computations are carried out with AutoDock4.2 [74]) and a screening service named MTiOpenScreen [23]. The screening service implements the docking program AutoDock Vina [75] to perform virtual screening computations and two in-house prepared drug-like chemical libraries (a Diverse-library and library expected to be enriched in inhibitors of protein-protein interaction, the iPPI-library) are available. It is also possible to upload a library of up to 5000 molecules generated by the user. AutoDock Vina associates a gradient-based conformational search approach and an empirical scoring function. AutoDock Vina parameters as implemented in MTiOpenScreen service are the following: the grid resolution is set to 1 Å, the maximum number of binding modes to output is fixed to 10, and the exhaustiveness level (controlling the number of independent runs performed) is assigned to 8. To define the search space, the user can choose whether to simply provide a list of residues forming part of the binding site or to manually specify the grid dimensions and the grid center coordinates. In the updated MTiOpenScreen service reported here, we now provide the purchasable approved drugs, food and natural compounds libraries discussed above. The virtual screening results of the 1500 top ranked compounds are reported by the server. The file containing the predicted binding poses (in pdbqt and mol2 format) and the predicted binding affinity (kcal/mol) table can be downloaded. Screening the Drug-lib using the MTiOpenScreen service took approximately 2 hours per structure.

### Protein receptor preparation

MTiOpenScreen service allows the user to upload the protein receptor with two different formats (mol2 and pdb). Users can thus download the protein structures from the PDB and use them directly without any preparation step in the MTiOpenScreen service. However, as some PDB structures can be problematic (missing side chains, alternate side chains conformations, presence of crystallographic adjuvant molecules in the binding site, …), we recommend to prepare the protein structures for docking. One very simple two-step protein preparation protocol is available within the Chimera package, a free to academic and nonprofit users and user-friendly visualization tool [76]. The procedure is described in the Supplementary Data (Supplementary Figure 16).

### Test cases protein structure selection and grid calculation parameters

Fluspirilene. 368 holo X-ray crystallographic structures of human CDK2 are available in the Protein Data Bank [77]. We used as test dataset of CDK2 proteins the 44 CDK2 proteins used for the ensemble docking study of ref [35] (Supplementary Table 1). In order to use the same AutoDock Vina grid center for all the proteins of the test dataset, the 44 proteins were aligned using the 4EK4 structure as the reference. The x, y, z AutoDock Vina grid center coordinates used are −1.560, −7.963, 27.836 and the size of the search space was set to 20 Å x 20 Å x 20 Å.

Mebendazole. 36 holo X-ray crystallographic structures of human VEGFR2 are available in the PDB [77]. 23 holo VEGFR2 structures presenting missing crystallized residues in the binding site area were excluded and the 13 remaining structures were used for this study (Supplementary Table 2). In order to use the same AutoDock Vina grid center for all the proteins of the test dataset, the 17 proteins were aligned using the 2XIR structure as the reference. The x, y, z grid center coordinates used are 21.592, 24.657, 38.079 and the size of the search space was set to 20 Å x 20 Å x 20 Å.

Raloxifene. 3 apo structures of the human GP130 proteins including the GP130-IL6 interface domain are available in the PDB [77] (PDB ID: 1I1R, 1P9M, 3L5H) and were used for this study (Supplementary Table 3). In order to use the same AutoDock Vina grid center for all the proteins of the test dataset, the 36 proteins were aligned using the 1P9M structure as the reference. The x, y, z grid center coordinates used are −101.693, 216.308, 44.304 and the size of the search space was set to 20 Å x 20 Å x 20 Å.

Sulindac. 40 holo X-ray crystallographic structures of human AKR1C3 are available in the PDB [77]. 19 holo AKR1C3 structures presenting missing residues in the binding site area were excluded and the 21 remaining structures were used for this study (Supplementary Table 4). In order to use the same AutoDock Vina grid center for all the proteins of the test dataset, the 40 proteins were aligned using the 4WDT structure as the reference. The x, y, z grid center coordinates used are −4.179, 26.732, 33.010 and the size of the search space was set to 20 Å x 20 Å x 20 Å.

Thalidomide. 7 holo X-ray crystallographic structures of human cereblon are available in the PDB [77] and were used for this study (Supplementary Table 5). In order to use the same AutoDock Vina grid center for all the proteins of the test dataset, the 7 proteins were aligned using the 5FQD structure as the reference. The x, y, z grid center coordinates used are 44.356, 130.694, 17.347 and the size of the search space was set to 20 Å x 20 Å x 20 Å.

### Statistical analysis

Graphics were produced using the statistical and graphical tool R (http://www.r-project.org/). The ChemmineR package [78] was used to compute atom pair fingerprints and PubChem's fingerprints for distance matrix computation. Supplementary Figures 1 and 2 were plotted with the ggplot2 package [79] and Figure 1 was plotted with the fmsb package (https://cran.r-project.org/web/packages/fmsb/index.html). DataWarrior [31] was used to perform the principal component analysis using the FragFp fingerprint descriptors and to produce the Supplementary Figure 4. DataWarrior [31] was also used to evaluate the chemical diversity of compounds using the FragFp fingerprint descriptors and to produce the Supplementary Figure 5, 6, 7, 9 and 11 with the defined similarity limit set to 80%. The Figure 5 was produced with the InteractiVenn web-based tool [80].

## SUPPLEMENTARY MATERIALS FIGURES AND TABLES





## Abbreviations

AKR1C3: aldo-keto reductase 1C3
CDK2: cyclin-dependent kinase 2
ER: estrogen receptor
FDA: food drug administration
HBA: hydrogen bond acceptor
HBD: hydrogen bond donor
IL-6: interleukin-6
logP: partition coefficient
MW: molecular weight
nrotB: number of rotatable bonds
NSAID: non-steroidal anti-inflammatory drug
PDB: Protein Data Bank
RMSD: Root Mean Square Deviation
SERM: selective estrogen receptor modulator
TMFS: train, match, fit, streamline
TPSA: topological polar surface area
VEGFR2: vascular endothelial growth factor 2
