# Supplementary material for: Online structure-based screening of purchasable approved drugs and natural compounds: retrospective examples of drug repositioning on cancer targets
Source: Oncotarget. 2018 Aug 17;9(64):32346–61. doi: 10.18632/oncotarget.25966 (PMC6122352; doi:10.18632/oncotarget.25966)
Supplement: Supplementary file 1 [file oncotarget-09-32346-s001.pdf]

Online structure-based screening of purchasable approved drugs and natural compounds: retrospective examples of drug repositioning on cancer targets

SUPPLEMENTARY MATERIALS

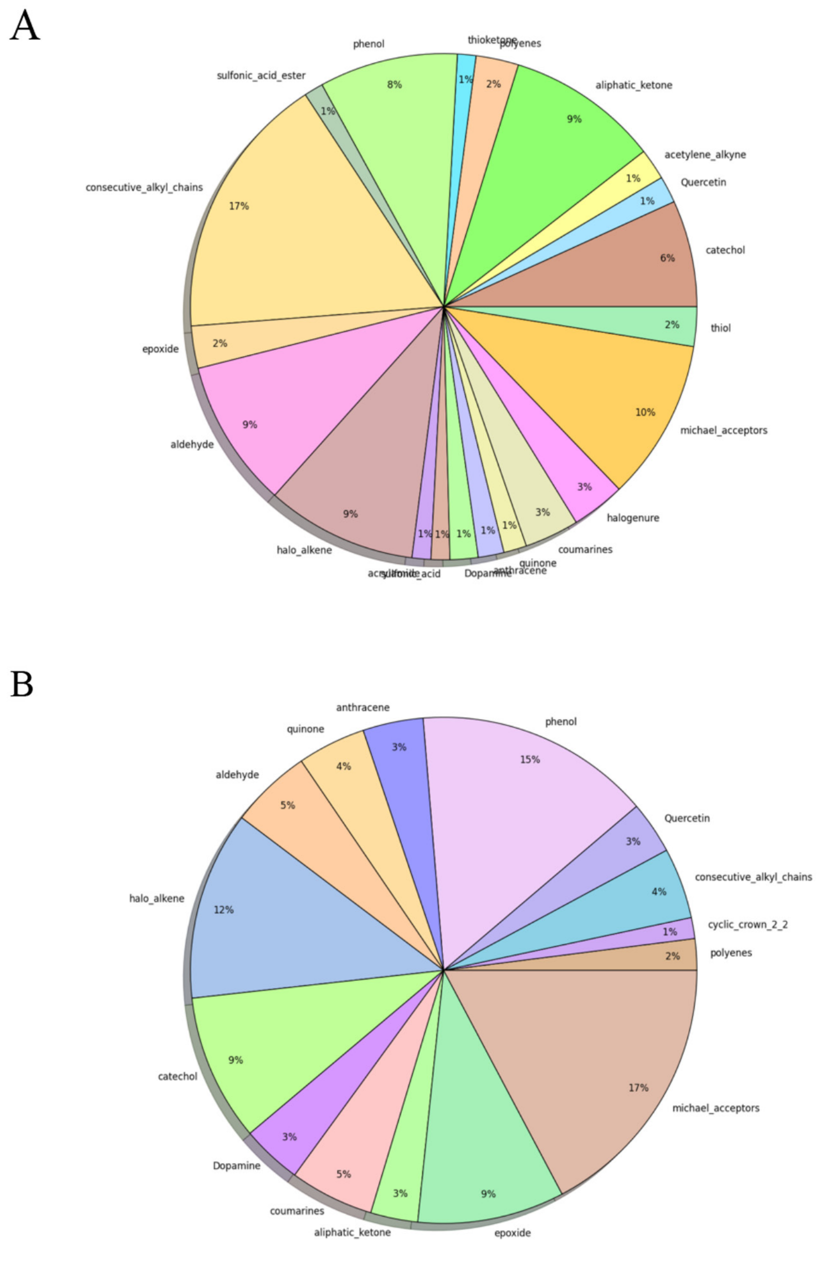

Supplementary Figure 1: Graphical representation of the problematic moieties identified using the of FAF-*Drugs4* toxicophore-like filters for the FOOD-lib **(A)** and the NP-lib **(B)**.

A

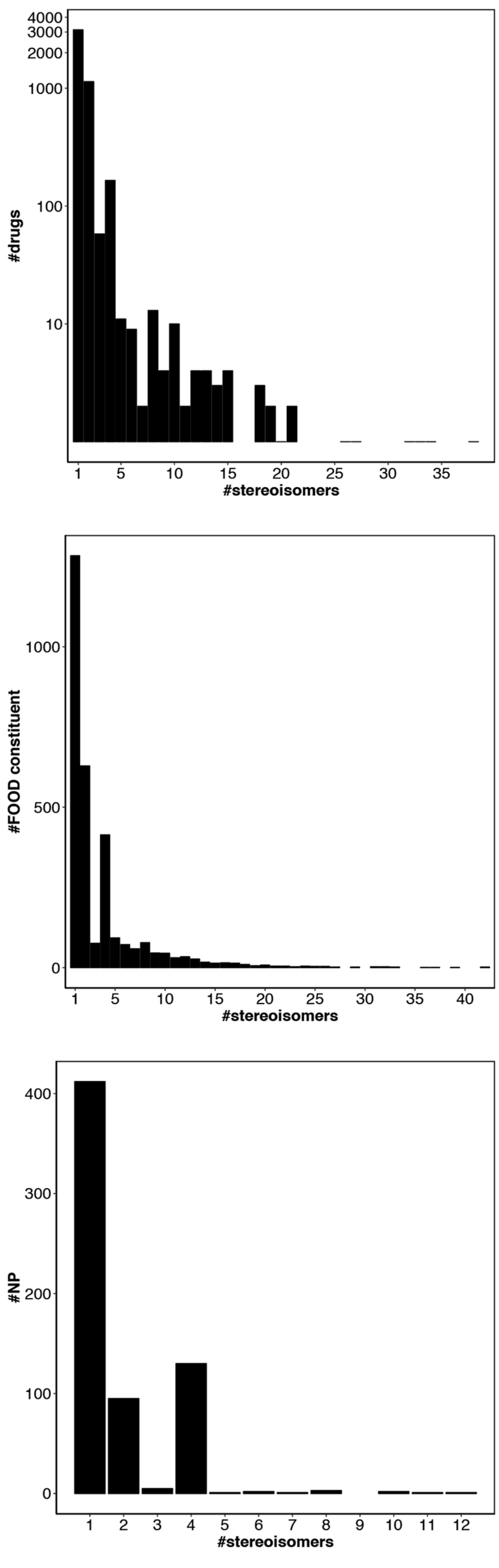

B

C

Supplementary Figure 2: Distribution of the number of stereoisomers per **(A)** drug for the Drugs-lib, **(B)** FOOD constituent for the FOOD-lib and **(C)** natural product (NP) for the NP-lib. For visualization convenience, the y-axis of the plot (A) is in log10 scale.

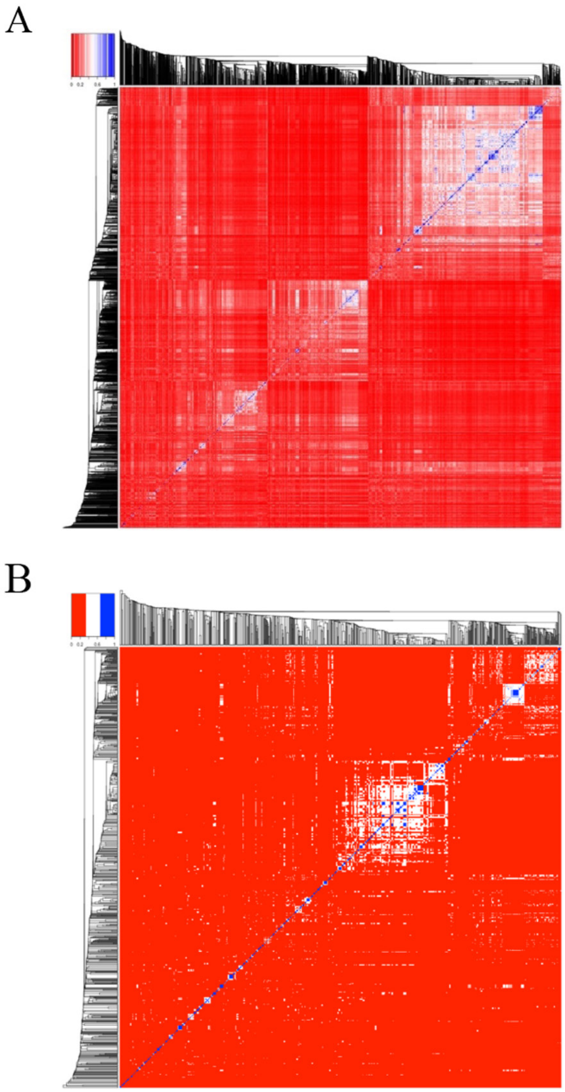

Supplementary Figure 3: Heatmap representation of (1 - distance matrix) for the FOOD-lib **(A)** and the NP-lib **(B)**. The distance matrices were computed using atom pair fingerprints and the dendogram was computed using PubChem's fingerprints. The closer the regions of the grid are to the color red, the less similar the pair of ligands.

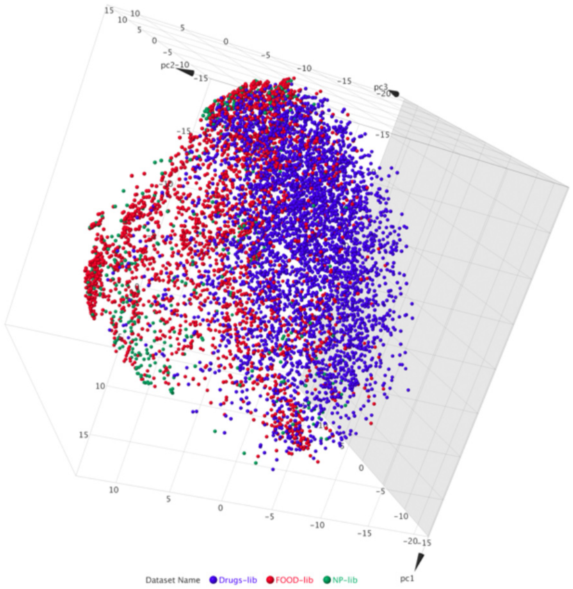

Supplementary Figure 4: 3D-view of the Principal Component Analysis results for the Drugs-lib (blue), FOOD-lib (red) and NP-lib (green), with the first three components from the FragFp descriptor assigned to the axes.

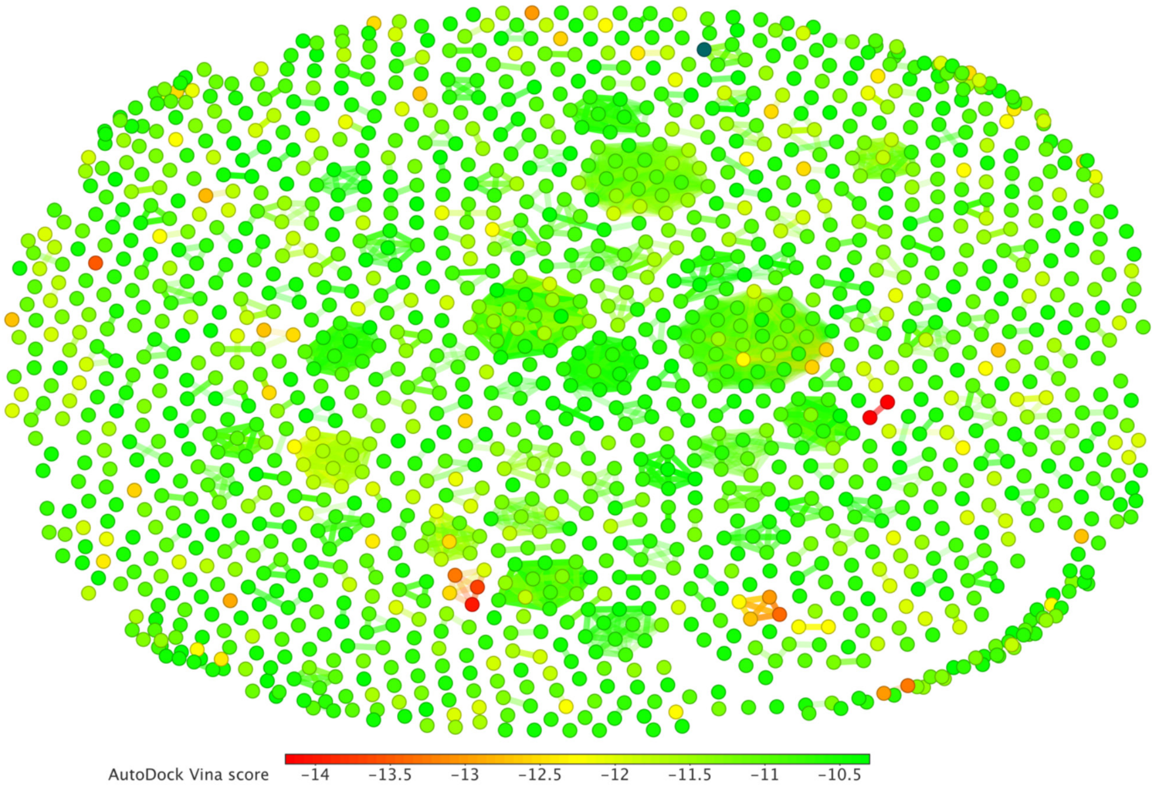

Supplementary Figure 5: Visualization of the 1500 best scored compounds for the CDK2 target (PDB structure 4EK4) using the 2D-Ruber Band Scaling approach implemented in DataWarrior. The compounds having high chemical similarity are connected by lines. Increased transparency of the lines reflects decreasing similarity. Fluspirilene is shown in dark green. The other compounds are colored according to their predicted AutoDock Vina score.

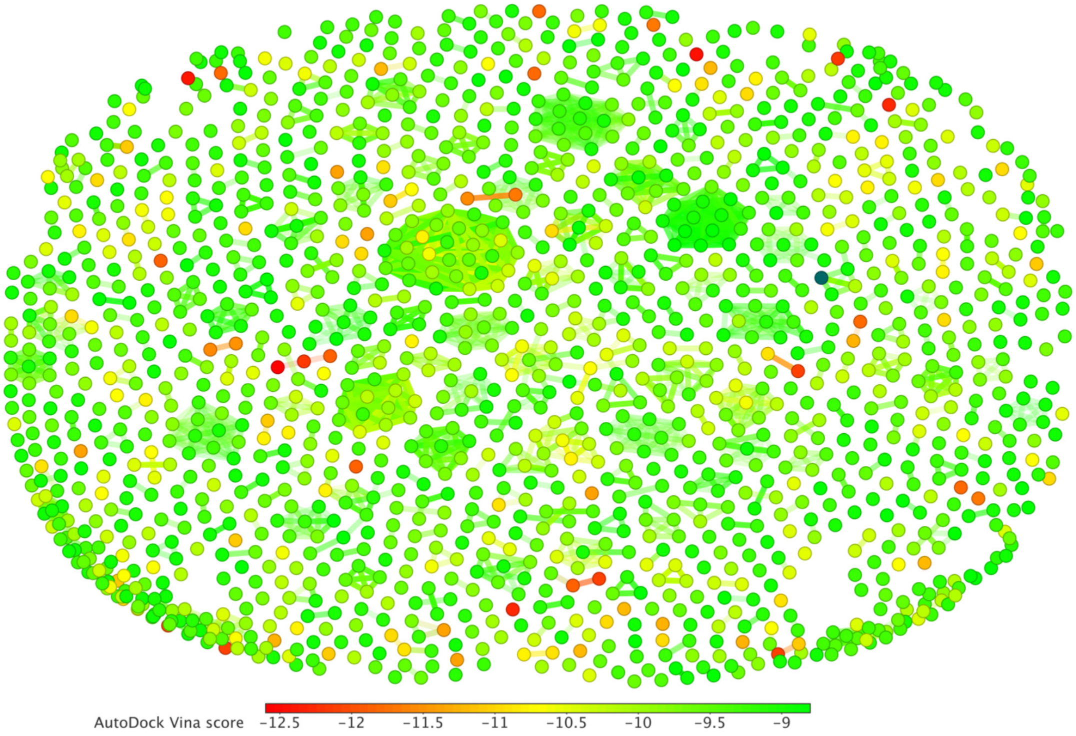

Supplementary Figure 6: Visualization of the 1500 best scored compounds for the VEGFR2 target (PDB structure 2XIR) using the 2D-Ruber Band Scaling approach implemented in DataWarrior. The compounds having high chemical similarity are connected by lines. Increased transparency of the lines reflects decreasing similarity. Mebendazole is shown in dark green. The other compounds are colored according to their predicted AutoDock Vina score.

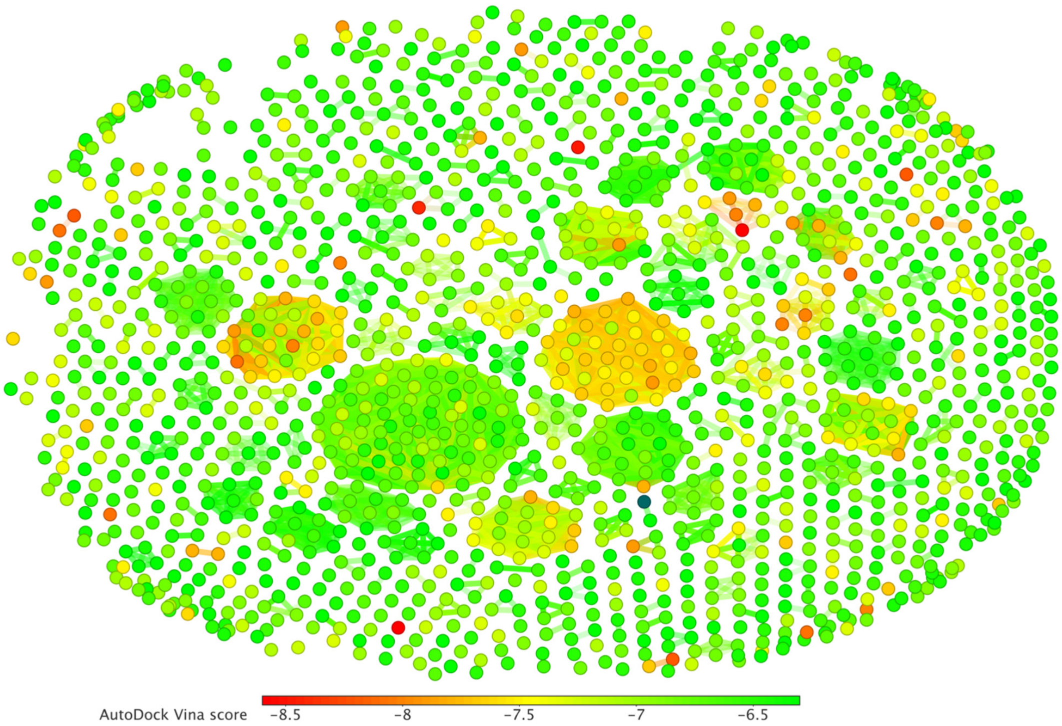

Supplementary Figure 7: Visualization of the 1500 best scored compounds for the GP130 target (PDB structure 1P9M) using the 2D-Ruber Band Scaling approach implemented in DataWarrior. The compounds having high chemical similarity are connected by lines. Increased transparency of the lines reflects decreasing similarity. Raloxifene is shown in dark green. The other compounds are colored according to their predicted AutoDock Vina score.

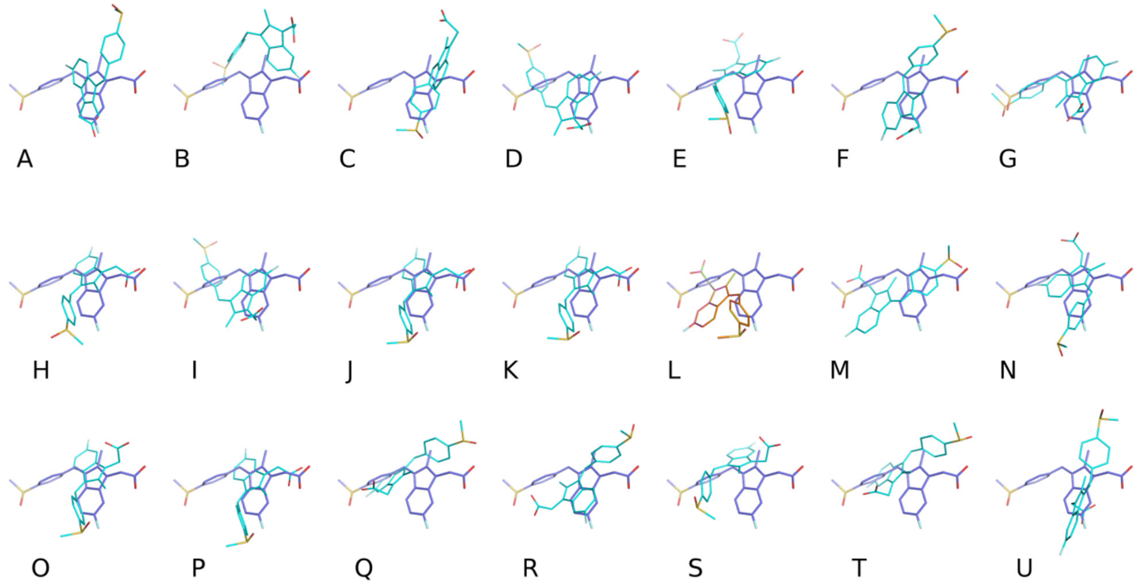

Supplementary Figure 8: Comparison of the sulindac experimental binding mode to AKR1C3 (as observed in the 3R7M PDB structure, in blue) and of the Sulindac predicted binding mode to AKR1C3 (docked pose associated with the best score, in cyan) obtained during the virtual screening performed with MTiOSs of the Drugs-lib against **(A)** 1RY0, **(B)** 1RY8, **(C)** 1S2A, **(D)** 1S2C, **(E)** 1XF0, **(F)** 2F38, **(G)** 3R43, **(H)** 4DBS, **(I)** 4DBU, **(J)** 4DBW, **(K)** 4FAM, **(L)** 4WDT (the pose associated with the best score in the 5 different runs are perfectly superimposed and are colored in cyan, magenta, orange, green and yellow), **(M)** 4WDU, **(N)** 4WRH, **(O)** 4XVD, **(P)** 4XVE, **(Q)** 4YVV, **(R)** 4YVX, **(S)** 4ZFC, **(T)** 5HNT, **(U)** 5JM5.

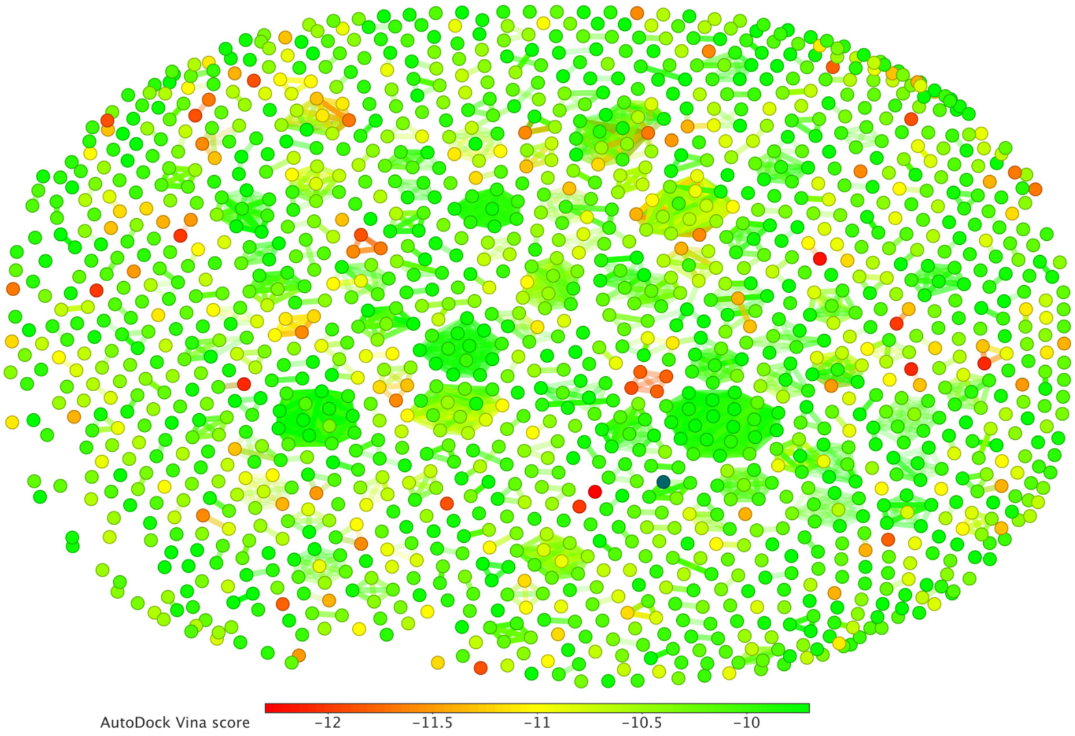

Supplementary Figure 9: Visualization of the 1500 best scored compounds for the AKR1C3 target (PDB structure 4WDT) using the 2D-Ruber Band Scaling approach implemented in DataWarrior. The compounds having high chemical similarity are connected by lines. Increased transparency of the lines reflects decreasing similarity. Sulindac is shown in dark green. The other compounds are colored according to their predicted AutoDock Vina score.

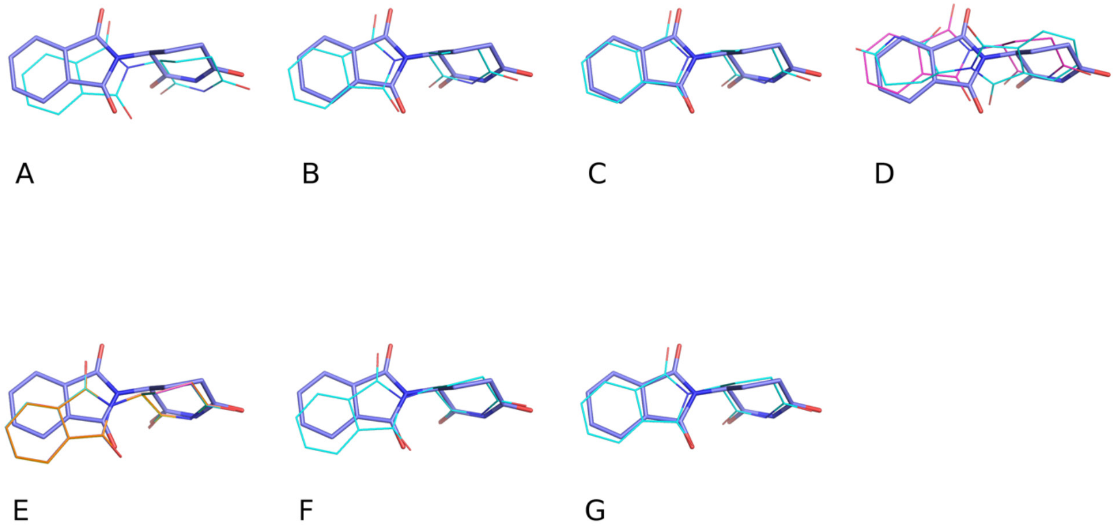

Supplementary Figure 10: Comparison of the Thalidomide experimental binding mode to cereblon (as observed in the 4CI1 PDB structure, in blue) and of the Thalidomide predicted binding mode to cereblon (docked pose associated with the best score, in cyan) obtained during the virtual screening performed with MTiOSs of the Drugs-lib against the different holo X-Ray structures of cereblon available in the PDB: **(A)** 4CI1, **(B)** 4CI2, **(C)** 4CI3, **(D)** 4TZ4, **(E)** 5FQD (the pose associated with the best score in the 5 different runs are perfectly superimposed and are colored in cyan, magenta, orange, green and yellow), **(F)** 5HXB and **(G)** 5V3O. It is to note that for 4TZ4 **(D)**, the first pose (in cyan) was not fitting the experimental binding mode, but the second pose (in magenta) was.

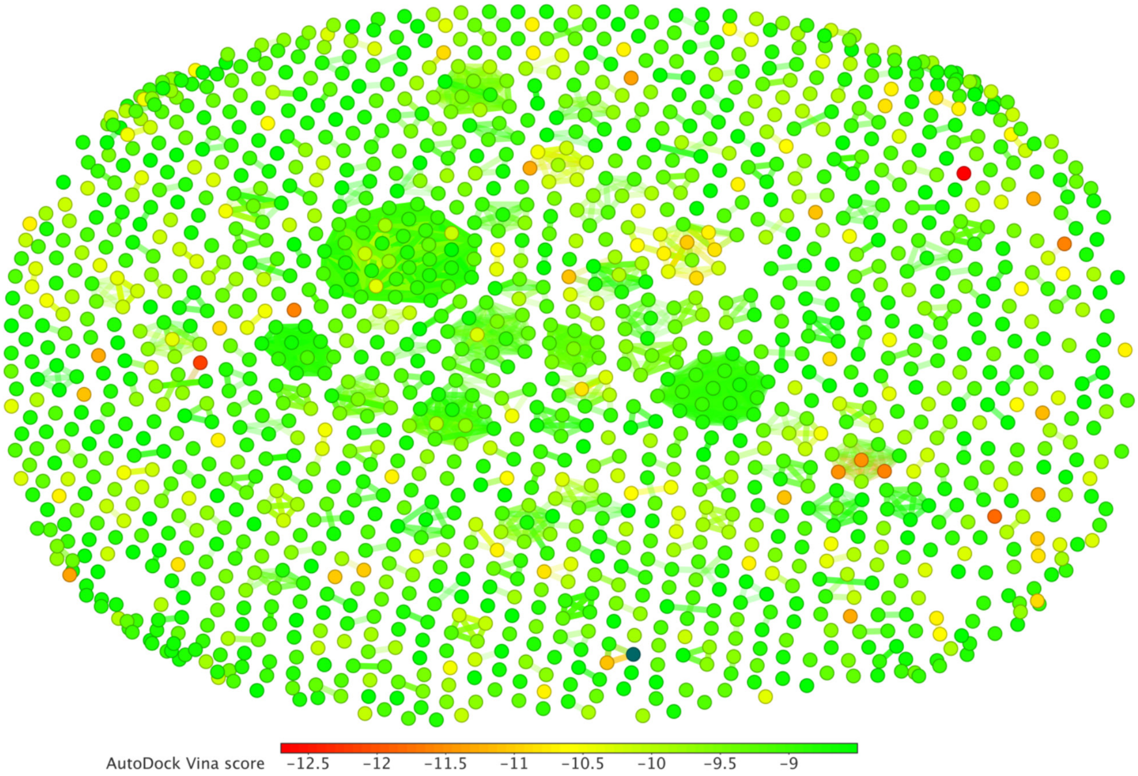

Supplementary Figure 11: Visualization of the 1500 best scored compounds for the cereblon target (PDB structure 5FQD) using the 2D-Ruber Band Scaling approach implemented in DataWarrior. The compounds having high chemical similarity are connected by lines. Increased transparency of the lines reflects decreasing similarity. Thalidomide is shown in dark green. The other compounds are colored according to their predicted AutoDock Vina score.

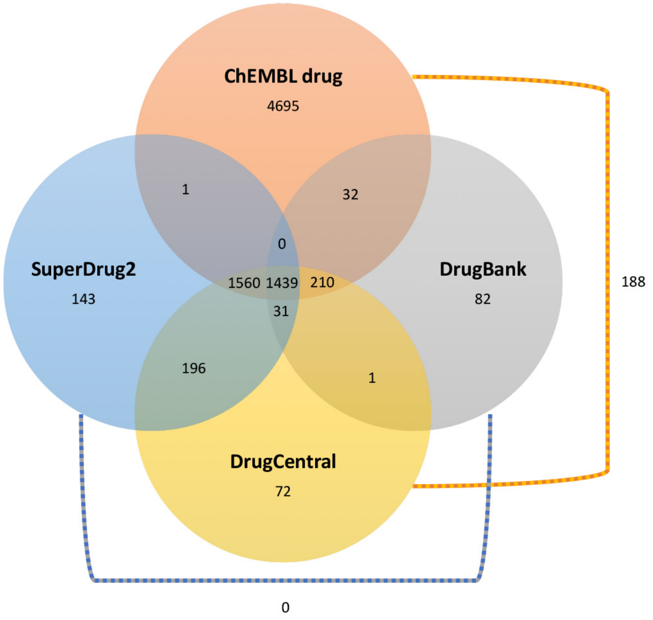

Supplementary Figure 12: Venn diagram representing the overlapping between the ChEMBL "drug" subset (in orange), the DrugBank "approved" subset (in grey), the DrugCentral online compendium (in yellow) and the SuperDrug2 "approved" database (in blue). The overlapping was computed by using the babel duplicates remove command line (--unique /nostereo/nochg option) on the 4 databases after the first filtration step with FAF-*Drugs* 4.

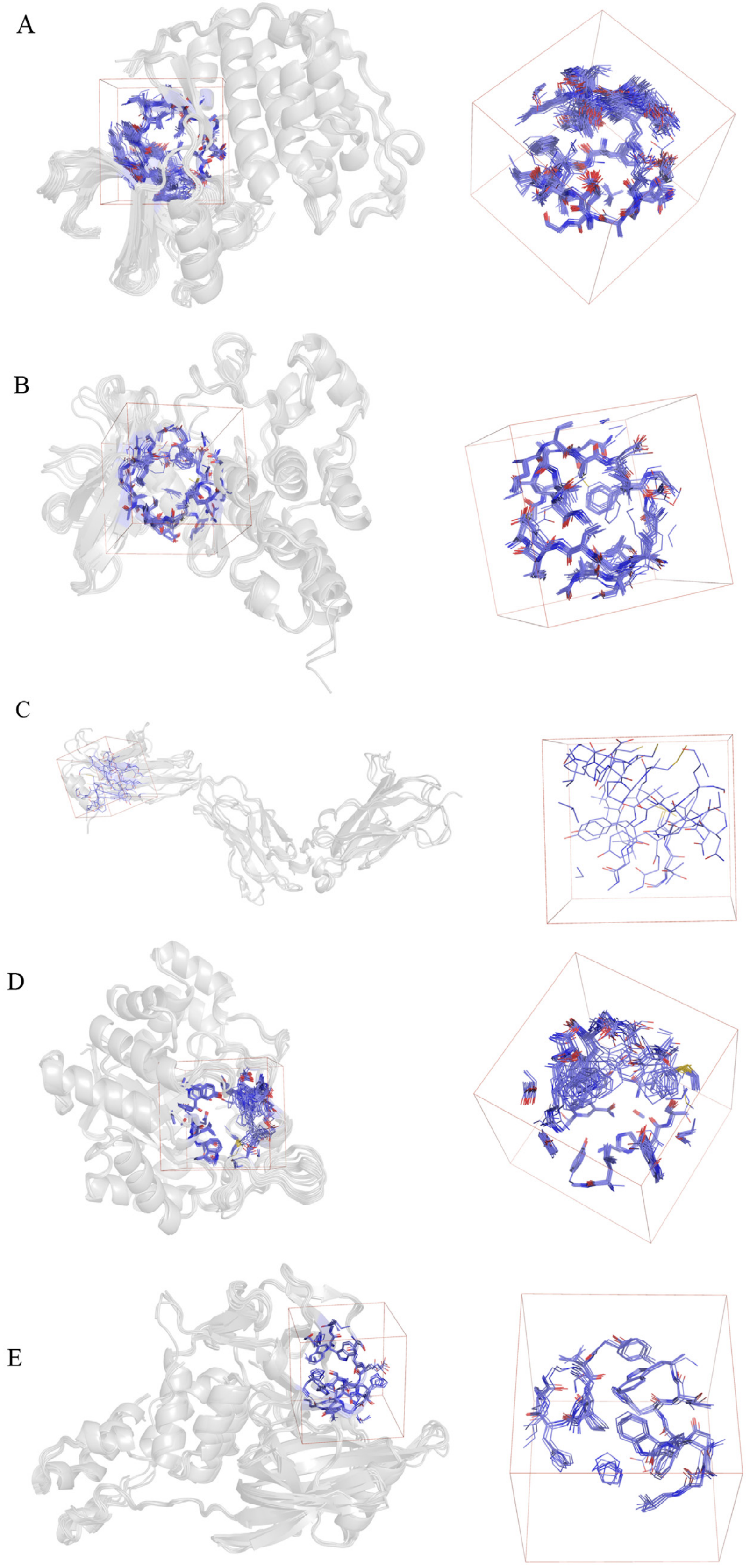

Supplementary Figure 13: Visual representation of the AutoDock Vina box search space (in red) for **(A)** CDK2, **(B)** VEGFR2, **(C)** GP130, **(D)** AKR1C3, **(E)** Cerebon. All structures used for this study are superimposed and represented in gray cartoon, the residues of the binding sites are represented in blue lines. The left panels enable to locate the binding sites within the whole protein, whereas the right panels provide a more detailed view of the conformational flexibility that append in the binding site.

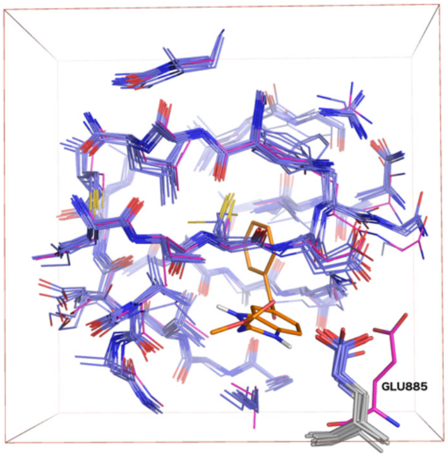

Supplementary Figure 14: Predicted binding mode for mebendazole (in orange) within the 3U6J PDB structure (in magenta). The binding site residues conformations of the other 12 VEGFR2 PDB structures are shown in blue. GLU885 presents a conformation different in the 3U6J PDB structure compared to the other VEGFR2 structures.

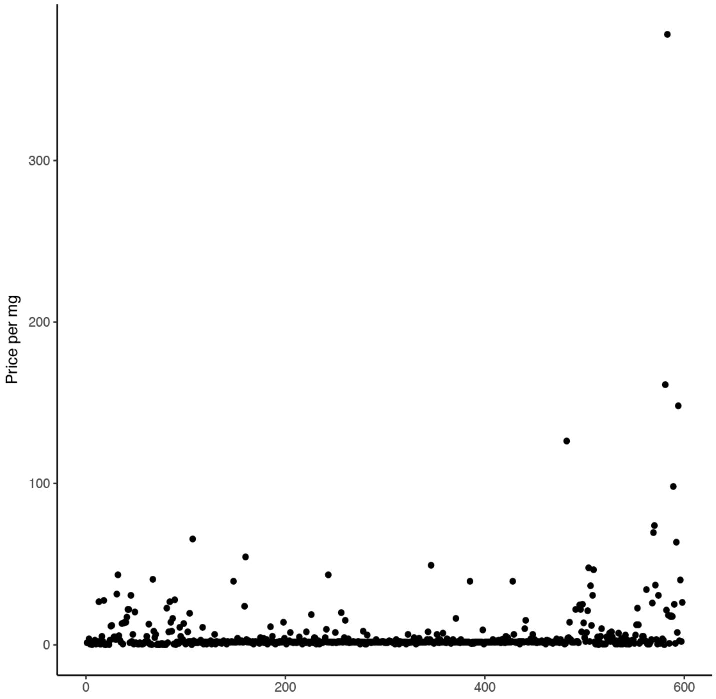

Supplementary Figure 15: Price per mg of 598 compounds of the Drugs-lib retrieved from the Sigma Aldrich website (https://www.sigmaaldrich.com/).

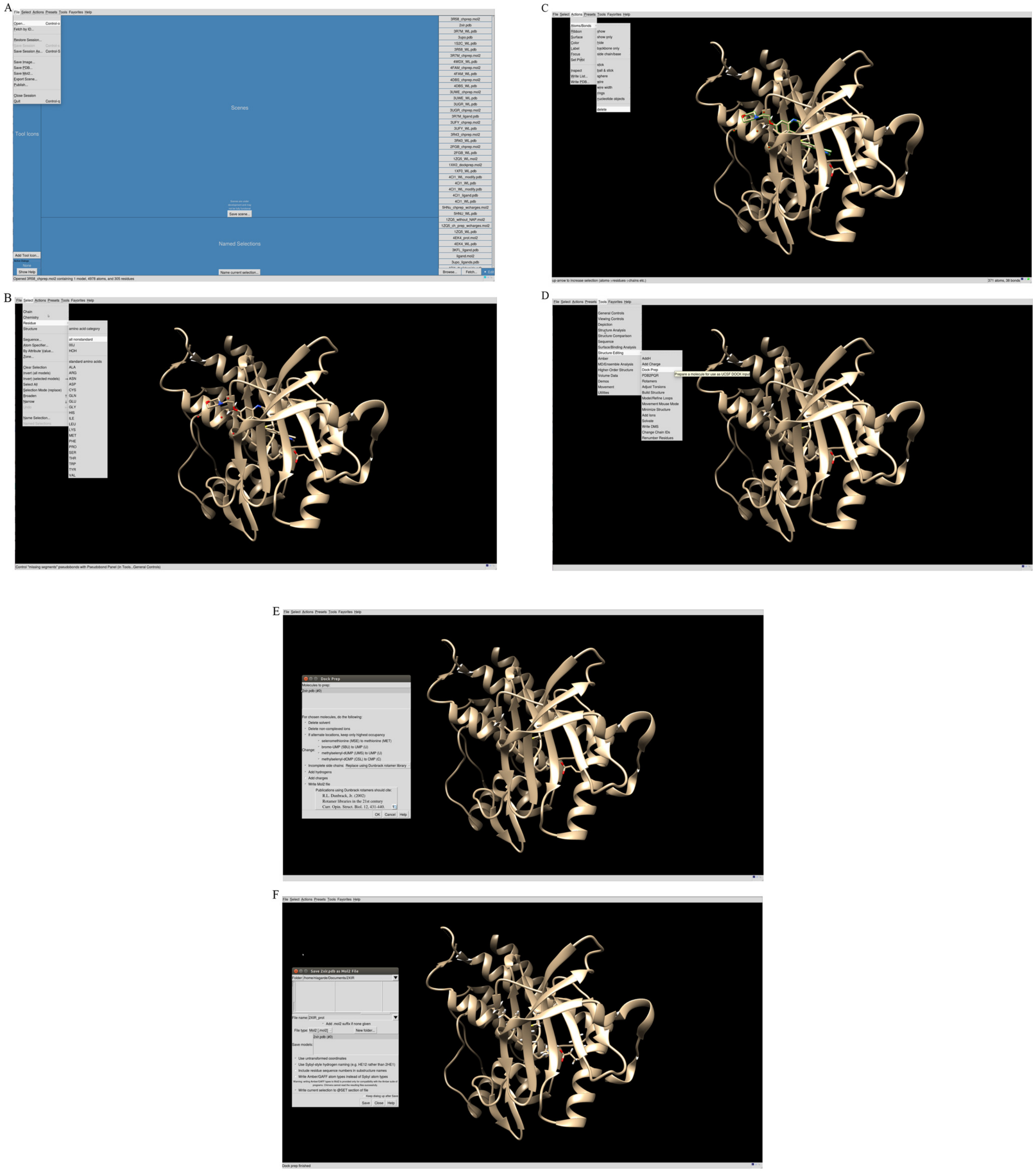

Supplementary Figure 16: The protein receptor preparation can be achieved using a two-step protocol and the Chimera package, a free to academic and nonprofit users and user-friendly visualization tool. 1. After opening the PDB structure in the Chimera graphical interface **(A)**, the user should select all non standard residues (except co-factors located in the binding site) **(B)** and delete them **(C)**. 2. The Dock Prep tool should then be applied **(D)** with all default options except the “Add charges” option that should be deselected (AutoDock Vina uses a specific charges format, and the charges will be added by MTiOSs during the conversion of the protein receptor form mol2 to the AutoDock Vina specific pdbqt format) **(E)**. The prepared protein structure can then be saved with the mol2 format **(F)**.

Supplementary Table 1: MTiOpenScreen outcomes for Fluspirilene obtained by screening the Drugs-lib on the 44 selected CDK2 X-Ray structures. The first and second column indicates the PDB ID of the X-Ray structure used (for 4EK4, 5 runs were proceeded and are labelled S1, S2, S3, S4 and S5) and its corresponding resolution. The third column presents the score attributed by AutoDock Vina to Fluspirilene. The fourth column represents the rank associated with Fluspirilene, computed by ranking all compounds of the Drugs-lib according to their AutoDock Vina Score. The last column represents the rank associated with Fluspirilene when considering only one occurrence (the best) of each drug.

PDB ID	Resolution (Å)	AutoDock Vina score	Rank (/7173)	Unique rank (/4574)
1AQ1	2.0	-10.0	566	451
1CKP	2.05	-8.7	770	614
1DI8	2.2	-9.7	648	503
1DM2	2.1	-10.1	322	265
1E1V	1.95	-8.5	1526	1173
1E1X	1.85	-10.1	209	178
1FVT	2.2	-9.2	1000	756
1G5S	2.61	-10.1	373	302
1GIH	2.8	-10.4	233	209
1GII	2.0	-9.7	284	233
1GIJ	2.2	-10.1	249	220
1GZ8	1.3	-8.5	906	686
1H00	1.6	-10.2	169	144
1H01	1.79	-10.4	281	235
1H07	1.85	-9.8	395	309
1H08	1.8	-9.9	344	287
1H0V	1.9	-9.1	297	248
1H0W	2.1	-9.8	185	132
1JSV	1.96	-8.5	946	732
1JVP	1.53	-12.2	620	494
1KE5	2.2	-9.7	271	236
1KE6	2.0	-9.6	475	403
1KE7	2.0	-9.4	324	269
1KE8	2.0	-9.6	254	221
1KE9	2.0	-10.2	117	104
1OIQ	2.31	-9.8	124	109
1OIR	1.91	-10.0	443	343
1OIT	1.6	-9.7	327	273
1P2A	2.5	-10.5	73	66
1PF8	2.51	-10.0	530	438
1PXI	1.95	-8.7	1048	805
1PXJ	2.3	-8.9	765	600
1PXK	2.8	-8.4	943	726
1PXL	2.5	-8.9	308	250
1PXM	2.53	-9.4	245	220
1PXN	2.5	-10.8	83	78
1PXO	1.96	-11.1	48	45
1PXP	2.3	-10.4	176	161
1PYE	2.0	-10.0	130	109
1R78	2.0	-10.3	206	176
1URW	1.6	-10.5	102	95
1V1K	2.31	-9.5	245	211
1VYZ	2.21	-9.2	559	443
1W0X	2.2	-9.8	401	327
4EK4 – S1	1.26	-9.3	186	152
4EK4 – S2	1.26	-9.4	149	120
4EK4 – S3	1.26	-9.3	190	154
4EK4 – S4	1.26	-9.4	154	124
4EK4 – S5	1.26	-9.4	157	126
4KFL	1.26	-8.8	889	657

Supplementary Table 2: MTiOpenScreen outcomes for Mebendazole obtained by screening the Drugs-lib on the 13 selected holo human VEGFR2 X-Ray structures. The first and second column indicates the PDB ID of the X-Ray structure used (for 2XIR, 5 runs were proceeded and are labelled S1, S2, S3, S4 and S5) and its corresponding resolution. The third column presents the score attributed by AutoDock Vina to Mebendazole. The fourth column represents the rank associated with Mebendazole, computed by ranking all compounds of the Drugs-lib according to their AutoDock Vina Score. The last column represents the rank associated with Mebendazole when considering only one occurrence (the best) of each drug.

PDB ID	Resolution (Å)	AutoDock Vina score	Rank (/7173)	Unique rank (/4574)
2XIR – S1	1.5	-9.5	713	553
2XIR – S2	1.5	-9.6	585	472
2XIR – S3	1.5	-9.6	604	484
2XIR – S4	1.5	-9.5	703	542
2XIR – S5	1.5	-9.5	684	528
3EWH	1.6	-8.9	1448	1089
3U6J	2.15	-9.1	1793	1321
3VHE	1.55	-9.8	684	529
3VHK	2.49	-9.1	717	543
3VNT	1.64	-8.6	1363	994
3VO3	1.52	-9.1	1327	990
3WZE	1.9	-9.4	730	582
4AG8	1.95	-10.6	205	179
4AGC	2.0	-10.8	51	46
4AGD	2.81	-9.3	310	256
4ASD	2.03	-8.6	1372	995
4ASE	1.83	-9.1	1012	771

Supplementary Table 3: MTiOpenScreen outcomes for Raloxifene obtained by screening the Drugs-lib on the 3 GP130 X-Ray structures including the GP130/IL-6 D1 domain. The first and second column indicates the PDB ID of the X-Ray structure used (for 1P9M, 5 runs were proceeded and are labelled S1, S2, S3, S4 and S5) and its corresponding resolution. The third column presents the score attributed by AutoDock Vina to Raloxifene. The fourth column represents the rank associated with Raloxifene, computed by ranking all compounds of the Drugs-lib according to their AutoDock Vina Score. The last column represents the rank associated with Raloxifene when considering only one occurrence (the best) of each drug.

PDB ID	Resolution (Å)	AutoDock Vina score	Rank (/7173)	Unique rank (/4574)
1I1R	2.4	-6.4	1012	692
1P9M – S1	3.65	-6.5	1088	737
1P9M – S2	3.65	-6.4	1269	860
1P9M – S3	3.65	-6.4	1268	858
1P9M – S4	3.65	-6.5	1091	743
1P9M – S5	3.65	-6.4	1270	858
3L5H	3.6	-7.5	151	126

Supplementary Table 4: MTiOpenScreen outcomes for Sulindac obtained by screening the Drugs-lib on the 21 selected holo human AKR1C3 X-Ray structures. The first and second column indicates the PDB ID of the X-Ray structure used (for 4WDT, 5 runs were proceeded and are labelled S1, S2, S3, S4 and S5) and its corresponding resolution. The third column presents the score attributed by AutoDock Vina to Sulindac. The fourth column represents the rank associated with Sulindac, computed by ranking all compounds of the Drugs-lib according to their AutoDock Vina Score. The fifth column represents the rank associated with Sulindac when considering only one occurrence (the best) of each drug. The last column reports the RMSD values in angstroms (Å) computed between the Sulindac experimental binding mode (as observed in the 3R7M PDB structure) and the Sulindac predicted pose outputted by MTiOSs

PDB ID	Resolution (Å)	AutoDock Vina score	Rank (/7173)	Unique rank (/4574)	RMSD
1RY0	1.69	-10.9	894	665	7.912
1RY8	1.69	-10.6	836	622	5.034
1S2A	1.7	-10.7	569	451	6.030
1S2C	1.8	-9.4	1618	1198	4.605
1XF0	2.0	-11.0	398	336	5.948
2F38	2.0	-10.7	1290	883	7.421
3R43	2.0	-9.8	1301	974	3.649
4DBS	1.85	-10.5	839	641	5.830
4DBU	2.53	-9.2	2250	1645	4.535
4DBW	1.8	-10.8	354	259	5.996
4FAM	2.0	-11.1	208	177	5.907
4WDT – S1	1.5	-10.1	817	652	7.297
4WDT – S2	1.5	-10.1	847	668	7.186
4WDT – S3	1.5	-10.1	854	670	7.200
4WDT – S4	1.5	-10.1	778	614	7.200
4WDT – S5	1.5	-10.1	858	679	5.334
4WDU	1.7	-10.6	647	518	7.310
4WRH	1.6	-10.7	1152	876	6.147
4XVD	2.81	-10.1	1121	850	6.200
4XVE	1.55	-10.0	979	737	6.191
4YVV	2.3	-10.5	1020	770	7.550
4YVX	2.3	-10.8	724	559	7.318
4ZFC	2.0	-10.3	1173	858	5.384
5HNT	2.0	-10.2	1707	1235	7.620
5JM5	1.99	-11.4	464	376	7.685

Supplementary Table 5: MTiOpenScreen outcomes for Thalidomide obtained by screening the Drugs-lib on the 7 holo human Cereblon X-Ray structures. The first and second column indicates the PDB ID of the X-Ray structure used (for 5FQD, 5 runs were proceeded and are labelled S1, S2, S3, S4 and S5) and its corresponding resolution. The third column presents the score attributed by AutoDock Vina to Thalidomide. The fourth column represents the rank associated with Thalidomide, computed by ranking all compounds of the Drugs-lib according to their AutoDock Vina Score. The fifth column represents the rank associated with Thalidomide when considering only one occurrence (the best) of each drug. The last column reports the RMSD values in angstroms (Å) computed between the Thalidomide experimental binding mode (as observed in the 4CI1 PDB structure) and the Thalidomide predicted pose outputted by MTiOSs.

PDB ID	Resolution (Å)	AutoDock Vina score	Rank (/7173)	Unique rank (/4574)	RMSD (Å)
4CI1	2.98	-9.8	226	196	0.760
4CI2	2.95	-9.6	176	154	0.565
4CI3	3.5	-9.7	212	188	0.349
4TZ4	3.01	-9.1	637	517	6.179
5FQD – S1	2.45	-11.0	24	21	1.132
5FQD – S2	2.45	-11.0	27	24	1.133
5FQD – S3	2.45	-10.9	25	24	1.125
5FQD – S4	2.45	-11.0	24	19	1.041
5FQD – S5	2.45	-11.0	24	22	1.055
5HXB	3.6	-10.5	53	51	0.608
5V3O	3.2	-9.9	138	121	0.436

Supplementary Table 6: List of the 7173 compounds in the Drugs-lib, with their generic drug denomination and the corresponding ZINC ID for each stereoisomer.

See Supplementary File 1
