# Supplementary material for: Online structure-based screening of purchasable approved drugs and natural compounds: retrospective examples of drug repositioning on cancer targets
Source: Oncotarget. 2018 Aug 17;9(64):32346–61. doi: 10.18632/oncotarget.25966 (PMC6122352; doi:10.18632/oncotarget.25966)
Supplement: Supplementary file 2 [file oncotarget-09-32346-s002.docx]

Supplementary Table 6: List of the 7173 compounds in the Drugs-lib, with their generic drug denomination and the corresponding ZINC ID for each stereoisomer.

| Drug name | ZINC ID |
| --- | --- |
| Abacavir | ZINC000002015928 |
| Abamectin-component-b1a | ZINC000242649028, ZINC000242649029, ZINC000242649030, ZINC000242649031, ZINC000242649032, ZINC000245224132, ZINC000245224134, ZINC000248170378, ZINC000252673974, ZINC000252673975, ZINC000252673976, ZINC000252694903, ZINC000257373234, ZINC000306147032, ZINC000306147033, ZINC000306147034, ZINC000306147035, ZINC000575231316 |
| Abemaciclib | ZINC000072318121 |
| Abexinostat | ZINC000006716700 |
| Abitesartan | ZINC000003806120 |
| Abt-102 | ZINC000028964595, ZINC000028964600 |
| Abt-107 | ZINC000038304971 |
| Abt-126 | ZINC000821864283 |
| Abunidazole | ZINC000001481713, ZINC000005114332 |
| Ac-430 | ZINC000095079935, ZINC000095079936 |
| Ac-480 | ZINC000006717782, ZINC000072190221 |
| Acadesine | ZINC000003798074 |
| Acaprazine | ZINC000004214705 |
| Acebutolol | ZINC000000156792, ZINC000000968255 |
| Acecainide | ZINC000001999404 |
| Aceclofenac | ZINC000003805798 |
| Acedapsone | ZINC000000000861 |
| Acefluranol | ZINC000000607681, ZINC000031490233, ZINC000031490235, ZINC000031490237 |
| Acefylline_clofibrol | ZINC000000608152 |
| Acefylline | ZINC000000057633 |
| Acemetacin | ZINC000000601272 |
| Aceperone | ZINC000004214717 |
| Acepromazine | ZINC000000057198 |
| Aceprometazine | ZINC000001481714, ZINC000002019621 |
| Acequinoline | ZINC000001481715 |
| Acetamidoeugenol | ZINC000000000007 |
| Acetaminosalol | ZINC000000000865 |
| Acetazolamide | ZINC000003813042 |
| Acetergamine | ZINC000004172319, ZINC000005196949 |
| Acetohexamide | ZINC000018067894 |
| Acetomenaphthone | ZINC000000000867 |
| Acetophenazine | ZINC000022446634 |
| Acetryptine | ZINC000000000870 |
| Acetyldihydrocodeine | ZINC000004214723 |
| Acetylpheneturide | ZINC000003871376, ZINC000003871377 |
| Acitazanolast | ZINC000001532937 |
| Acitemate | ZINC000004214730, ZINC000004626715, ZINC000005858388, ZINC000005858390 |
| Acodazole | ZINC000018009339 |
| Acolbifene | ZINC000000598598 |
| Aconiazide | ZINC000000043420 |
| Acotiamide | ZINC000002002237 |
| Acoxatrine | ZINC000001640527, ZINC000005196905 |
| Acridorex | ZINC000004172322, ZINC000005550642 |
| Acriflavine | ZINC000003775641 |

Table S1. (continued next page)

| Drug name | ZINC ID |
| --- | --- |
| Acronine | ZINC000004098812 |
| Actinoquinol | ZINC000001645025 |
| Actisomide | ZINC000028755841, ZINC000028755844 |
| Acyclovir | ZINC000001530555 |
| Adafenoxate | ZINC000004214735 |
| Adamexine | ZINC000004214737 |
| Adapalene | ZINC000003784182 |
| Adaprolol | ZINC000003777540, ZINC000005442107 |
| Adatanserin | ZINC000053046001 |
| Adefovir-dipivoxil | ZINC000003930376 |
| Adefovir | ZINC000021297308 |
| Aderbasib | ZINC000034997457 |
| Adibendan | ZINC000000000883 |
| Adimolol | ZINC000001999500, ZINC000001999501 |
| Adinazolam | ZINC000004214740 |
| Adiphenine | ZINC000001717372 |
| Adipiplon | ZINC000030691732 |
| Aditeren | ZINC000000000884 |
| Aditoprim | ZINC000000000011 |
| Adomeglivant | ZINC000117040414, ZINC000117040417 |
| Adosopine | ZINC000000012519 |
| Adrafinil | ZINC000000000012, ZINC000004611316 |
| Adrogolide | ZINC000030690682 |
| Aee-788 | ZINC000022453679, ZINC000033196300 |
| Afacifenacin | ZINC000001554383, ZINC000003991067 |
| Afalanine | ZINC000000135391, ZINC000000135392 |
| Afimoxifene | ZINC000008602413 |
| Afloqualone | ZINC000013831145 |
| Afuresertib | ZINC000043197674 |
| Afurolol | ZINC000003649841, ZINC000003649842 |
| Ag-13958 | ZINC000034037183, ZINC000095909141 |
| Ag-24322 | ZINC000200158122 |
| Aganodine | ZINC000000000886 |
| Agomelatine | ZINC000000005608 |
| Ajmaline | ZINC000008220548, ZINC000014880005, ZINC000014880007, ZINC000100069270, ZINC000105824024 |
| Alacepril | ZINC000003775143 |
| Alagebrium | ZINC000003916477 |
| Alamifovir | ZINC000003964493 |
| Alaproclate | ZINC000052969655, ZINC000052969658 |
| Alatrofloxacin | ZINC000100067477 |
| Alazanine | ZINC000004824262 |
| Albaconazole | ZINC000000602086 |
| Albendazole | ZINC000017146904 |
| Albuterol | ZINC000000007601, ZINC000000020230 |
| Alclofenac | ZINC000002014875 |
| Aleglitazar | ZINC000049573657 |
| Alentemol | ZINC000000004092, ZINC000001851382 |
| Aleplasinin | ZINC000013580622 |
| Alepride | ZINC000004172332, ZINC000005157863 |

Table S1. (continued next page)

| Drug name | ZINC ID |
| --- | --- |
| Aletamine | ZINC000001481718, ZINC000001678582 |
| Alfatradiol | ZINC000000002298, ZINC000000125040, ZINC000000518397, ZINC000001319879, ZINC000002036738, ZINC000002041353, ZINC000003201368, ZINC000003815415, ZINC000003830763, ZINC000003830764, ZINC000003830765, ZINC000003875384, ZINC000003881869, ZINC000003881870, ZINC000003978002, ZINC000004521752, ZINC000005275868, ZINC000009231977, ZINC000013520815 |
| Alfentanil | ZINC000000601281 |
| Alfuzosin | ZINC000001542392, ZINC000001843099 |
| Alibendol | ZINC000001842915 |
| Aliconazole | ZINC000000608163 |
| Alifedrine | ZINC000001999497 |
| Alimadol | ZINC000004214760 |
| Alinastine | ZINC000003800366 |
| Alinidine | ZINC000000003723 |
| Alisertib | ZINC000040939534 |
| Aliskiren | ZINC000004393164 |
| Alitame | ZINC000002020491 |
| Alizapride | ZINC000002001364, ZINC000002001365 |
| Alloclamide | ZINC000001843032 |
| Allopurinol | ZINC000013298313 |
| Allylprodine | ZINC000000000021, ZINC000005459478, ZINC000027877188 |
| Almestrone | ZINC000004214770 |
| Alminoprofen | ZINC000000000022, ZINC000001999253 |
| Almitrine | ZINC000004214772 |
| Almotriptan | ZINC000000018087 |
| Almoxatone | ZINC000004214773 |
| Alnespirone | ZINC000003635820 |
| Alniditan | ZINC000001536693 |
| Aloin | ZINC000004214775, ZINC000100018557 |
| Alonacic | ZINC000084759261, ZINC000084759262 |
| Alonimid | ZINC000002035715, ZINC000011677082 |
| Alosetron | ZINC000013537284 |
| Alovudine | ZINC000001725270 |
| Alpelisib | ZINC000068198368, ZINC000169302884 |
| Alpertine | ZINC000004214776 |
| alpha_Ergocryptine | ZINC000014952151, ZINC000017654092, ZINC000059796556, ZINC000059796557, ZINC000095810539, ZINC000105284878, ZINC000222341315, ZINC000575417835 |
| Alphameprodine | ZINC000001850976 |
| Alphaprodine | ZINC000001087483, ZINC000004626719 |
| Alpidem | ZINC000000599598 |
| Alpiropride | ZINC000000607703, ZINC000001999471 |
| Alprafenone | ZINC000001533706, ZINC000002015986 |
| Alprazolam | ZINC000000000903 |
| Alprenolol | ZINC000000000023, ZINC000000086599 |
| Alrestatin | ZINC000003871501 |
| Altapizone | ZINC000006096074 |
| Althiazide | ZINC000002037169, ZINC000002037170 |
| Altinicline | ZINC000003826943 |
| Altiratinib | ZINC000113198271 |

Table S1. (continued next page)

| Drug name | ZINC ID |
| --- | --- |
| Altretamine | ZINC000000000905 |
| Alvameline | ZINC000000006302 |
| Alverine | ZINC000001481966 |
| Alvimopan | ZINC000000598850, ZINC000000605670, ZINC000003782341, ZINC000003802417, ZINC000011677127, ZINC000026378311, ZINC000136850645 |
| Alvocidib | ZINC000021288966 |
| Am-211 | ZINC000043206277 |
| Amanozine | ZINC000000000907 |
| Ambenonium | ZINC000003995599 |
| Ambenoxan | ZINC000001843055, ZINC000001843057 |
| Ambrisentan | ZINC000000538627 |
| Ambroxol | ZINC000100001905 |
| Ambruticin | ZINC000008214482 |
| Ambucaine | ZINC000002018952 |
| Ambucetamide | ZINC000002036733, ZINC000002036734 |
| Ambuside | ZINC000100378867 |
| Ambutonium | ZINC000001700052 |
| Amdoxovir | ZINC000003919810 |
| Amedalin | ZINC000002015641, ZINC000002015642 |
| Amesergide | ZINC000083261402 |
| Amezepine | ZINC000003627189 |
| Amezinium | ZINC000000000908 |
| Amfenac | ZINC000000000909 |
| Amfepentorex | ZINC000001842983, ZINC000001842985 |
| Amflutizole | ZINC000000000910 |
| Amfonelic-acid | ZINC000000000911 |
| Amg-208 | ZINC000034285235 |
| Amg-517 | ZINC000014974132 |
| Amg-548 | ZINC000013982572, ZINC000073336080 |
| Amg-900 | ZINC000043208325 |
| Amibegron | ZINC000003797401 |
| Amicarbalide | ZINC000000031235 |
| Amicibone | ZINC000000607709, ZINC000002015221 |
| Amidantel | ZINC000100375414 |
| Amidephrine_mesylate | ZINC000049902433, ZINC000095060664 |
| Amiflamine | ZINC000000000915, ZINC000029212975 |
| Amifloverine | ZINC000000000916 |
| Amifloxacin | ZINC000000000917 |
| Amiglumide | ZINC000003780994 |
| Amikhelline | ZINC000000000918 |
| Amiloride | ZINC000004340269 |
| Aminacrine | ZINC000019014768 |
| Amindocate | ZINC000000000919 |
| Amino-diphenylhydantoin-valeric-acid | ZINC000038613690, ZINC000038613692 |
| Aminopentamide-sulfate | ZINC000000000030, ZINC000002020048 |
| Aminopromazine | ZINC000019366084, ZINC000022442730 |
| Aminopropylone | ZINC000000000031, ZINC000005211994 |

Table S1. (continued next page)

| Drug name | ZINC ID |
| --- | --- |
| Altretamine | ZINC000000000905 |
| Alvameline | ZINC000000006302 |
| Alverine | ZINC000001481966 |
| Alvimopan | ZINC000000598850, ZINC000000605670, ZINC000003782341, ZINC000003802417, ZINC000011677127, ZINC000026378311, ZINC000136850645 |
| Alvocidib | ZINC000021288966 |
| Am-211 | ZINC000043206277 |
| Amanozine | ZINC000000000907 |
| Ambenonium | ZINC000003995599 |
| Ambenoxan | ZINC000001843055, ZINC000001843057 |
| Ambrisentan | ZINC000000538627 |
| Ambroxol | ZINC000100001905 |
| Ambruticin | ZINC000008214482 |
| Ambucaine | ZINC000002018952 |
| Ambucetamide | ZINC000002036733, ZINC000002036734 |
| Ambuside | ZINC000100378867 |
| Ambutonium | ZINC000001700052 |
| Amdoxovir | ZINC000003919810 |
| Amedalin | ZINC000002015641, ZINC000002015642 |
| Amesergide | ZINC000083261402 |
| Amezepine | ZINC000003627189 |
| Amezinium | ZINC000000000908 |
| Amfenac | ZINC000000000909 |
| Amfepentorex | ZINC000001842983, ZINC000001842985 |
| Amflutizole | ZINC000000000910 |
| Amfonelic-acid | ZINC000000000911 |
| Amg-208 | ZINC000034285235 |
| Amg-517 | ZINC000014974132 |
| Amg-548 | ZINC000013982572, ZINC000073336080 |
| Amg-900 | ZINC000043208325 |
| Amibegron | ZINC000003797401 |
| Amicarbalide | ZINC000000031235 |
| Amicibone | ZINC000000607709, ZINC000002015221 |
| Amidantel | ZINC000100375414 |
| Amidephrine_mesylate | ZINC000049902433, ZINC000095060664 |
| Amiflamine | ZINC000000000915, ZINC000029212975 |
| Amifloverine | ZINC000000000916 |
| Amifloxacin | ZINC000000000917 |
| Amiglumide | ZINC000003780994 |
| Amikhelline | ZINC000000000918 |
| Amiloride | ZINC000004340269 |
| Aminacrine | ZINC000019014768 |
| Amindocate | ZINC000000000919 |
| Amino-diphenylhydantoin-valeric-acid | ZINC000038613690, ZINC000038613692 |
| Aminopentamide-sulfate | ZINC000000000030, ZINC000002020048 |
| Aminopromazine | ZINC000019366084, ZINC000022442730 |
| Aminopropylone | ZINC000000000031, ZINC000005211994 |

Table S1. (continued next page)

| Drug name | ZINC ID |
| --- | --- |
| Aminopterin | ZINC000002036915 |
| Aminoquinol | ZINC000003882238, ZINC000003882239 |
| Aminoquinuride | ZINC000022910880 |
| Amiperone | ZINC000004214827 |
| Amiprilose | ZINC000004211931 |
| Amiquinsin | ZINC000000000924 |
| Amisulpride | ZINC000000601255, ZINC000001846088 |
| Amitifadine | ZINC000000003189 |
| Amitraz | ZINC000100025258 |
| Amitriptyline | ZINC000000968257 |
| Amitriptylinoxide | ZINC000001481969 |
| Amixetrine | ZINC000000000035, ZINC000001842933 |
| Amlexanox | ZINC000000000928 |
| Amodiaquine | ZINC000000608172 |
| Amolanone | ZINC000006720796, ZINC000053021878 |
| Amonafide | ZINC000004214836 |
| Amoproxan | ZINC000031771396, ZINC000031771397 |
| Amopyroquine | ZINC000004214841 |
| Amorolfine | ZINC000013512582, ZINC000013512584 |
| Amosulalol | ZINC000001536931, ZINC000002019996 |
| Amotosalen | ZINC000002541705 |
| Amotriphene | ZINC000004214845 |
| Amoxapine | ZINC000000000931 |
| Amoxydramine | ZINC000000000932 |
| Amp579 | ZINC000256226376, ZINC000256226379, ZINC000256226382, ZINC000256226387 |
| Amperozide | ZINC000004214849 |
| Amprolium | ZINC000000000934 |
| Amprotropine | ZINC000002041004, ZINC000002041005 |
| Ampyrimine | ZINC000000000935 |
| Amquinate | ZINC000005315911 |
| Amsacrine | ZINC000003812923 |
| Amsalog | ZINC000005461015 |
| Amtolmetin_guacil | ZINC000000596929 |
| Amylmetacresol | ZINC000002039651 |
| Amylocaine | ZINC000000000038, ZINC000002034893 |
| Anagrelide | ZINC000003871541 |
| Anatibant | ZINC000053261548 |
| Ancarolol | ZINC000000000039, ZINC000005353020 |
| Ancitabine | ZINC000004027061 |
| Andolast | ZINC000000004680 |
| Androisoxazole | ZINC000004211958, ZINC000013640952 |
| Anidoxime | ZINC000004211961 |
| Anilamate | ZINC000000000950 |
| Aniracetam | ZINC000000015951 |
| Anirolac | ZINC000000000043, ZINC000001844865 |
| Anisindione | ZINC000100015486 |
| Anisopirol | ZINC000002040279, ZINC000004211966 |
| Anisotropine | ZINC000100036830 |
| Anitrazafen | ZINC000000000957 |

Table S1. (continued next page)

| Drug name | ZINC ID |
| --- | --- |
| Anpirtoline | ZINC000000000958 |
| Ansoxetine | ZINC000005957953, ZINC000005957963 |
| Antafenite | ZINC000000000044, ZINC000001559439 |
| Antazoline | ZINC000000057204 |
| Anthelmycin | ZINC000072267007 |
| Antienite | ZINC000004211973, ZINC000006093619 |
| Antrafenine | ZINC000053073961 |
| Apabetalone | ZINC000043199551 |
| Apadoline | ZINC000002012779 |
| Apafant | ZINC000000608180 |
| Apaxifylline | ZINC000000005156 |
| Apazone | ZINC000000005349, ZINC000002019585 |
| Apitolisib | ZINC000059224388 |
| Apixaban | ZINC000011677837 |
| Aplindore | ZINC000003952389 |
| Apraclonidine | ZINC000000020231 |
| Apramycin | ZINC000008214486, ZINC000079114506, ZINC000082149513, ZINC000082149515 |
| Apremilast | ZINC000030691736 |
| Aprepitant | ZINC000014768568, ZINC000027428708, ZINC000027428713, ZINC000043770782, ZINC000043770784, ZINC000052245742, ZINC000059125344, ZINC000097974568 |
| Apricoxib | ZINC000003941695 |
| Aprindine | ZINC000001420561 |
| Aprofene | ZINC000002003680 |
| Aptazapine | ZINC000000000050 |
| Aptiganel | ZINC000012466820 |
| Aptocaine | ZINC000000000051, ZINC000001999274 |
| Aqw051 | ZINC000003942685 |
| Ar-12 | ZINC000003960083 |
| Aranotin | ZINC000004214877 |
| Araprofen | ZINC000000011669, ZINC000005141751 |
| Arbaclofen | ZINC000000000061, ZINC000000085733 |
| Arbidol | ZINC000019907652 |
| Arbutamine | ZINC000001537194 |
| Arbutin | ZINC000000518554 |
| Arclofenin | ZINC000004214881 |
| Arfendazam | ZINC000000000965 |
| Argatroban | ZINC000003807172, ZINC000003917722 |
| Arhalofenate | ZINC000002012859, ZINC000002012860 |
| Arimoclomol | ZINC000000008826 |
| Aripiprazole | ZINC000001851149 |
| Arnolol | ZINC000002019987, ZINC000002019988 |
| Arofylline | ZINC000013658656 |
| Aronixil | ZINC000004214883 |
| Arotinolol | ZINC000001542905, ZINC000001846151 |
| Arprinocid | ZINC000000000969 |
| Arpromidine | ZINC000014951255, ZINC000014951267 |
| Articaine | ZINC000002019953, ZINC000002019954 |
| Artilide | ZINC000003782823 |
| Arzoxifene | ZINC000001544683 |
| Asapiprant | ZINC000115250035 |

Table S1. (continued next page)

| Drug name | ZINC ID |
| --- | --- |
| Asenapine | ZINC000000004893 |
| Aseripide | ZINC000082125620 |
| Asimadoline | ZINC000003800054 |
| Asobamast | ZINC000001537504 |
| Asp-3026 | ZINC000068120928 |
| Aspirin | ZINC000000000053 |
| Ast-487 | ZINC000023247639 |
| Astemizole | ZINC000000601274 |
| Astromicin | ZINC000004654843 |
| Asunaprevir | ZINC000085540202 |
| At-13148 | ZINC000095920747, ZINC000095938075 |
| At-7519 | ZINC000016052857 |
| At-9283 | ZINC000038995988 |
| Ataciguat | ZINC000003612878 |
| Ataluren | ZINC000013831791 |
| Ataprost | ZINC000004214891 |
| Ataquimast | ZINC000000006705 |
| Atenolol | ZINC000000014007, ZINC000000113415 |
| Atevirdine-mesylate | ZINC000000597817 |
| Atibeprone | ZINC000000005661 |
| Atipamezole | ZINC000005424171 |
| Atiprimod | ZINC000001540226 |
| Atiprosin | ZINC000019367444 |
| Atizoram | ZINC000003810794 |
| Atliprofen | ZINC000000003771, ZINC000006037116 |
| Atolide | ZINC000000000972 |
| Atomoxetine | ZINC000001842633, ZINC000002570822 |
| Atopaxar | ZINC000038913962 |
| Atorvastatin | ZINC000003920719 |
| Atrasentan | ZINC000003812144 |
| Atromepine | ZINC000100367555, ZINC000100367561 |
| Atropine-oxide | ZINC000000394477, ZINC000100092856, ZINC000100379436, ZINC000254042524, ZINC000254042528, ZINC000254043040, ZINC000255214899, ZINC000255214902, ZINC000261506608 |
| Atx08-001 | ZINC000003820333 |
| Avagacestat | ZINC000043202993 |
| Avanafil | ZINC000011677851, ZINC000011677857 |
| Avarofloxacin | ZINC000034661474 |
| Avasimibe | ZINC000001540245 |
| Avatrombopag | ZINC000072190218 |
| Avibactam | ZINC000009302239 |
| Avitriptan | ZINC000000600434 |
| Avizafone | ZINC000001532335 |
| Axamozide | ZINC000004212030, ZINC000006117407 |
| Axelopran | ZINC000103298099 |
| Axitinib | ZINC000003816287 |
| Axitirome | ZINC000001538134, ZINC000011677432 |
| Axomadol | ZINC000000021744 |
| Azabuperone | ZINC000020792463 |

Table S1. (continued next page)

| Drug name | ZINC ID |
| --- | --- |
| Azacitidine | ZINC000000967756, ZINC000001078622, ZINC000001482063, ZINC000003830251, ZINC000003830252, ZINC000003861768, ZINC000011592974, ZINC000011592975, ZINC000013547768, ZINC000021984062 |
| Azaclorzine | ZINC000019804668 |
| Azaconazole | ZINC000000000975 |
| Azacyclonol | ZINC000012375998 |
| Azaftozine | ZINC000022462753 |
| Azaloxan | ZINC000002021295 |
| Azamethiphos | ZINC000001999379 |
| Azamulin | ZINC000004214902 |
| Azanator | ZINC000000000978 |
| Azanidazole | ZINC000004214905 |
| Azaperone | ZINC000002596977 |
| Azapetine | ZINC000001481977 |
| Azaprocin | ZINC000038158054 |
| Azaribine | ZINC000004214908 |
| Azarole | ZINC000004214910 |
| Azasetron | ZINC000000004132, ZINC000002528474 |
| Azaspirium | ZINC000000608188 |
| Azatadine | ZINC000000968337 |
| Azathioprine | ZINC000004258316 |
| Azd0328 | ZINC000033961869, ZINC000113684442 |
| Azd1386 | ZINC000139948300, ZINC000139948444 |
| Azd1446 | ZINC000081213894, ZINC000081213895, ZINC000081213896 |
| Azd-1480 | ZINC000058631551, ZINC000066099927 |
| Azd-1775 | ZINC000063539231 |
| Azd1981 | ZINC000073196066 |
| Azd-2014 | ZINC000049019448, ZINC000059259287, ZINC000059259288 |
| Azd2066 | ZINC000034885049 |
| Azd2624 | ZINC000043169621, ZINC000043169623 |
| Azd3514 | ZINC000101673084 |
| Azd-4547 | ZINC000091352092, ZINC000091352093, ZINC000095616598 |
| Azd-4769 | ZINC000001914117, ZINC000013888387 |
| Azd4818 | ZINC000034773954 |
| Azd-5438 | ZINC000040442496 |
| Azd6280 | ZINC000071295757 |
| Azd-6482 | ZINC000038628584 |
| Azd-7624 | ZINC000002105296, ZINC000002105298 |
| Azd-7762 | ZINC000033359230, ZINC000034885165 |
| Azd-8055 | ZINC000052509466, ZINC000095567488, ZINC000115586131, ZINC000143201183 |
| Azd9056 | ZINC000034356159 |
| Azelastine | ZINC000000601229, ZINC000000897240 |
| Azepexole | ZINC000000000982 |
| Azepindole | ZINC000000000983 |
| Azetirelin | ZINC000003804057 |
| Azilsartan-medoxomil | ZINC000014210642 |
| Azilsartan | ZINC000000598390 |
| Azimilide | ZINC000021983255 |
| Azintamide | ZINC000000000984 |
| Azipramine | ZINC000001481979 |

Table S1. (continued next page)

| Drug name | ZINC ID |
| --- | --- |
| Azolimine | ZINC000000000985 |
| Azosemide | ZINC000005843546 |
| Aztreonam | ZINC000003830263, ZINC000003830264, ZINC000012503091, ZINC000015848211, ZINC000016958002, ZINC000017214369, ZINC000252430978, ZINC000256010240, ZINC000256010241, ZINC000256010242 |
| Azumolene | ZINC000005842585 |
| Bafetinib | ZINC000022940637 |
| Bagrosin | ZINC000026663179, ZINC000026663185 |
| baicalin | ZINC000003943903 |
| Bakeprofen | ZINC000006020170, ZINC000006035951 |
| Balaglitazone | ZINC000001481805, ZINC000001489816 |
| Balofloxacin | ZINC000002015981, ZINC000004654976 |
| Balsalazid | ZINC000003952881 |
| Bamaquimast | ZINC000001544908 |
| Bambuterol | ZINC000000597556, ZINC000002021931 |
| Bamifylline | ZINC000000608191 |
| Bamipine | ZINC000000000987 |
| Bamirastine | ZINC000003927238 |
| Bamnidazole | ZINC000001481981 |
| Baquiloprim | ZINC000000000989 |
| Barasertib | ZINC000043129461 |
| Barmastine | ZINC000004214938 |
| Barucainide | ZINC000003643459 |
| Basmisanil | ZINC000145814743 |
| Batabulin | ZINC000002003142 |
| Batefenterol | ZINC000096941866 |
| Batelapine | ZINC000031284355 |
| Batimastat | ZINC000003789788 |
| Batoprazine | ZINC000000004465 |
| Bavisant | ZINC000034962220 |
| Bazedoxifene | ZINC000001895505 |
| Becanthone | ZINC000004212058 |
| Becatecarin | ZINC000003950898 |
| Becliconazole | ZINC000003780731, ZINC000005425441 |
| Beclobrate | ZINC000004212060, ZINC000005651229 |
| Bedaquiline | ZINC000004655029 |
| Bederocin | ZINC000003821131 |
| Bedoradrine | ZINC000000584203 |
| Befetupitant | ZINC000028570456 |
| Befiradol | ZINC000001492397 |
| Befloxatone | ZINC000003776953 |
| Befunolol | ZINC000000000063, ZINC000002007481 |
| Befuraline | ZINC000019632922 |
| Belaperidone | ZINC000003822014 |
| Belarizine | ZINC000022463137 |
| Belnacasan | ZINC000003963010 |
| Belotecan | ZINC000003826691 |
| Bemarinone | ZINC000005424965 |
| Bemesetron | ZINC000100373652 |
| Bemetizide | ZINC000000607726, ZINC000001842744, ZINC000001842745, ZINC000001842746 |

Table S1. (continued next page)

| Drug name | ZINC ID |
| --- | --- |
| Bemitradine | ZINC000002017714 |
| Bemoradan | ZINC000005736150, ZINC000005752381 |
| Bemotrizinol | ZINC000011677890, ZINC000011677894, ZINC000011677901 |
| Benactyzine | ZINC000001734034 |
| Benafentrine | ZINC000003780828 |
| Benanserin | ZINC000000000998 |
| Benapryzine | ZINC000002032621 |
| Benaxibine | ZINC000000000066, ZINC000004000052 |
| Benazeprilat | ZINC000003814184 |
| Benazepril | ZINC000003781943 |
| Bencianol | ZINC000004214953 |
| Bencisteine | ZINC000006020916, ZINC000006036733, ZINC000072266849 |
| Benclonidine | ZINC000072266865, ZINC000072266866 |
| Bencyclane | ZINC000001999382 |
| Bendazac | ZINC000000001000 |
| Bendazol | ZINC000000113392 |
| Benderizine | ZINC000038655326 |
| Bendroflumethiazide | ZINC000000601301, ZINC000000897222 |
| Benfluorex | ZINC000002015039, ZINC000002015040 |
| Benfosformin | ZINC000004214960 |
| Benhepazone | ZINC000000001002 |
| Benorilate | ZINC000000001003 |
| Benoxaprofen | ZINC000000000070, ZINC000001872015 |
| Benoxinate | ZINC000002019492 |
| Benperidol | ZINC000009232411 |
| Benproperine | ZINC000001481729, ZINC000003635554 |
| Benrixate | ZINC000004214964 |
| Bensalan | ZINC000004214965 |
| Bentazepam | ZINC000005850754 |
| Bentemazole | ZINC000005425471 |
| Bentipimine | ZINC000022462814, ZINC000031392373 |
| Bentiromide | ZINC000000608204 |
| Benurestat | ZINC000000001008 |
| Benzalamide | ZINC000002029423, ZINC000033844914 |
| Benzaprinoxide | ZINC000000001009 |
| Benzarone | ZINC000000000071 |
| Benzbromarone | ZINC000000608205 |
| Benzestrol | ZINC000001600954, ZINC000003875595, ZINC000003875596, ZINC000003875597 |
| Benzethidine | ZINC000004214975 |
| Benzethonium | ZINC000001571009 |
| Benzhydryloxyethyl-diethyl-methylammonium | ZINC000005651729 |
| Benzilonium | ZINC000000607729, ZINC000002015882 |
| Benzindopyrine | ZINC000001481982 |
| Benznidazole | ZINC000000056949 |
| Benzobarbital | ZINC000000437559, ZINC000002899983 |
| Benzoclidine | ZINC000000000075, ZINC000000135992 |
| Benzoctamine | ZINC000001481983 |
| Benzomethamine | ZINC000002038381 |
| Benzopyrronium | ZINC000000000077, ZINC000001843001 |

Table S1. (continued next page)

| Drug name | ZINC ID |
| --- | --- |
| Benzotript | ZINC000002539823 |
| Benzoxiquine | ZINC000000001015 |
| Benzphetamine | ZINC000000968305, ZINC000000968306 |
| Benzpiperylon | ZINC000000001019 |
| Benzpyrinium | ZINC000000001020 |
| Benzthiazide | ZINC000003871698 |
| Benztropine | ZINC000100036536 |
| Benzydamine | ZINC000002020083 |
| Benzylhydrochlorothiazide | ZINC000000607731, ZINC000002015922 |
| Benzylsulfamide | ZINC000000001022 |
| Bepafant | ZINC000002016003, ZINC000013531919 |
| Beperidium | ZINC000004212118, ZINC000005688398 |
| Bephenium | ZINC000000001023 |
| Bepiastine | ZINC000000001024 |
| Bepotastine | ZINC000000602128 |
| Bepridil | ZINC000003812918, ZINC000003830283 |
| Berefrine | ZINC000003778687, ZINC000013901022 |
| Bergenin | ZINC000004046820 |
| Berlafenone | ZINC000000004353, ZINC000002019970 |
| Bermoprofen | ZINC000000000080, ZINC000001846075 |
| Bertosamil | ZINC000013891848 |
| Berupipam | ZINC000000598671 |
| Bervastatin | ZINC000001547675 |
| Besifloxacin | ZINC000003787097 |
| Besipirdine | ZINC000000004896 |
| Besonprodil | ZINC000027299440, ZINC000027299445 |
| Besulpamide | ZINC000005765093 |
| Besunide | ZINC000004214996 |
| beta_Ergocryptine | ZINC000059213491, ZINC000064633860, ZINC000100071818, ZINC000100071821 |
| Betahistine | ZINC000001675415 |
| Betamipron | ZINC000000012614 |
| Betiatide | ZINC000006095847 |
| Betoxycaine | ZINC000004215003 |
| Betrixaban | ZINC000030691754 |
| Bevenopran | ZINC000038896576 |
| Bevonium | ZINC000000607738, ZINC000001999350 |
| Bexagliflozin | ZINC000059047505, ZINC000072319782, ZINC000108646761, ZINC000108646764, ZINC000108646769, ZINC000208242340, ZINC000669679359, ZINC000888086749, ZINC000888086750 |
| Bexarotene | ZINC000001539579 |
| Bexlosteride | ZINC000003785769 |
| Bezafibrate | ZINC000003956919 |
| Bgt-226 | ZINC000073069304 |
| Bi-2536 | ZINC000013986815, ZINC000035851315 |
| Bialamicol | ZINC000004215006 |
| Bibenzonium | ZINC000000000085, ZINC000005764638 |
| Bicifadine | ZINC000001844916, ZINC000005781680 |
| Biclodil | ZINC000005784353 |
| Biclotymol | ZINC000004215009 |
| Bidimazium | ZINC000004215011 |

Table S1. (continued next page)

| Drug name | ZINC ID |
| --- | --- |
| Bidisomide | ZINC000004212146, ZINC000005442038 |
| Bietamiverine | ZINC000001842750, ZINC000001842752 |
| Bifemelane | ZINC000001542896 |
| Bifepramide | ZINC000001846099, ZINC000001846100 |
| Bifeprofen | ZINC000031502465, ZINC000031502469 |
| Bifeprunox | ZINC000052971454 |
| Bifluranol | ZINC000001999258, ZINC000001999259, ZINC000001999260, ZINC000004212152 |
| Bifonazole | ZINC000003812958, ZINC000003871723 |
| Bilastine | ZINC000003822702 |
| Bimatoprost | ZINC000003994921, ZINC000004474405, ZINC000011676867, ZINC000013532605, ZINC000013532609, ZINC000043772350, ZINC000043772352, ZINC000043772354, ZINC000043772356, ZINC000043773182, ZINC000043773184, ZINC000043773186, ZINC000043773188, ZINC000043898683, ZINC000045789171, ZINC000045789174, ZINC000045789177, ZINC000045789180, ZINC000052509427, ZINC000059867924, ZINC000071789572, ZINC000071789573, ZINC000117044865, ZINC000248375379, ZINC000248375380, ZINC000306120456, ZINC000306120457, ZINC000306120458, ZINC000334161402, ZINC000622788413, ZINC000725422835, ZINC000725422836, ZINC000725422837 |
| Bimethoxycaine | ZINC000001648449, ZINC000001648450, ZINC000001648452 |
| Bimoclomol | ZINC000000006126, ZINC000006093425 |
| Bindarit | ZINC000000004594 |
| Binedaline | ZINC000000001032 |
| Binfloxacin | ZINC000000596925 |
| Binifibrate | ZINC000003812859 |
| Binodenoson | ZINC000003932652 |
| Binospirone | ZINC000001536786, ZINC000001999423 |
| Bioresmethrin | ZINC000000001033, ZINC000000900670, ZINC000000900673, ZINC000000900675 |
| Bipenamol | ZINC000000056761 |
| Biperiden | ZINC000000968361, ZINC000001481738 |
| Biphenamine | ZINC000001999340 |
| Biricodar | ZINC000003945332 |
| Birinapant | ZINC000096941868 |
| Biriperone | ZINC000019366609 |
| Bisacodyl | ZINC000003830321 |
| Bisantrene | ZINC000001550957, ZINC000001577736 |
| Bisfenazone | ZINC000000608210 |
| Bisfentidine | ZINC000071325802 |
| Bisoctrizole | ZINC000011677911 |
| Bisoprolol | ZINC000001530569, ZINC000001530570 |
| Bisoxatin-acetate | ZINC000000608211 |
| Bisoxatin | ZINC000002017901 |
| Bisulepine | ZINC000002004348, ZINC000002004349 |
| Bithionoloxide | ZINC000000608214 |
| Bithionol | ZINC000000608213 |
| Bitolterol | ZINC000000896452, ZINC000003830323 |
| Bitopertin | ZINC000053294263 |
| Blonanserin | ZINC000000597434 |
| Bms-387032 | ZINC000003816409 |
| Bms-582949 | ZINC000036475284 |
| Bms-690514 | ZINC000043152599, ZINC000098091230, ZINC000115584290, ZINC000115584461 |
| Bms-754807 | ZINC000043203317, ZINC000145213336 |

Table S1. (continued next page)

| Drug name | ZINC ID |
| --- | --- |
| Bms-777607 | ZINC000039716080 |
| Bms-833923 | ZINC000096170449 |
| Bms-863233 | ZINC000084668615, ZINC000299853377 |
| Bms-911543 | ZINC000100468481 |
| Bolazine | ZINC000008214506 |
| Bopindolol | ZINC000001542901, ZINC000002032320 |
| Bornaprine | ZINC000004212177, ZINC000072266812 |
| Bornaprolol | ZINC000004212179, ZINC000004626622, ZINC000071325805, ZINC000071325806 |
| Bosentan | ZINC000001538857 |
| Botiacrine | ZINC000000001038 |
| Boxidine | ZINC000000001039 |
| Bradanicline | ZINC000064540331 |
| Brasofensine | ZINC000003945986 |
| Brazergoline | ZINC000004215062 |
| Brecanavir | ZINC000003994828 |
| Brefonalol | ZINC000004212184, ZINC000005439974 |
| Bremazocine | ZINC000014952631 |
| Brequinar | ZINC000001587011 |
| Bretazenil | ZINC000000597316 |
| Bretylium | ZINC000000001041 |
| Brexpiprazole | ZINC000084758479 |
| Brigatinib | ZINC000148723177 |
| Brimonidine | ZINC000021303210 |
| Brinazarone | ZINC000001541062 |
| Brindoxime | ZINC000004212188, ZINC000072266863 |
| Brinzolamide | ZINC000001530900, ZINC000003953037 |
| Brivanib | ZINC000013684256 |
| Brivaracetam | ZINC000003979899 |
| Brivudine | ZINC000003653378 |
| Broclepride | ZINC000004215067 |
| Brocresine | ZINC000000001045 |
| Brocrinat | ZINC000004215069 |
| Brodimoprim | ZINC000000005824 |
| Brofaromine | ZINC000000001046 |
| Brofezil | ZINC000000000091, ZINC000006037202 |
| Brofoxine | ZINC000002016193 |
| Brolaconazole | ZINC000005117378, ZINC000005117397 |
| Brolamfetamine | ZINC000000000092, ZINC000000402865 |
| Bromadoline | ZINC000004215075 |
| Bromamid | ZINC000000001049 |
| Bromazepam | ZINC000000001051 |
| Bromchlorenone | ZINC000000001052 |
| Bromerguride | ZINC000004215078 |
| Bromfenac | ZINC000002570817 |
| Bromhexine | ZINC000000608220 |
| Bromindione | ZINC000100003605 |
| Bromocriptine | ZINC000053683151 |
| Bromodiphenhydramine | ZINC000000000095, ZINC000001668588 |
| Bromopride | ZINC000002038104 |

Table S1. (continued next page)

| Drug name | ZINC ID |
| --- | --- |
| Bromosalicychloranilidine | ZINC000000001178 |
| Bromoxanide | ZINC000056897575 |
| Bromperidol | ZINC000000601270 |
| Brompheniramine | ZINC000000000096, ZINC000000896455 |
| Broparestrol | ZINC000008214508 |
| Broperamole | ZINC000000608223 |
| Bropirimine | ZINC000016051409 |
| Broquinaldol | ZINC000000001061 |
| Brosotamide | ZINC000004215099 |
| Brosuximide | ZINC000002019956, ZINC000095564734 |
| Brotizolam | ZINC000002570830 |
| Brovanexine | ZINC000004212197 |
| Brovincamine | ZINC000049925472 |
| Broxaldine | ZINC000000608225 |
| Broxaterol | ZINC000000000098, ZINC000001999557 |
| Broxitalamic-acid | ZINC000004215106 |
| Broxuridine | ZINC000001081243 |
| Broxyquinoline | ZINC000000001064 |
| Bucetin | ZINC000000000099, ZINC000000156801 |
| Buciclovir | ZINC000000001065 |
| Bucladesine | ZINC000003861742 |
| Buclizine | ZINC000019364226, ZINC000019364228 |
| Buclosamide | ZINC000001846512 |
| Bucloxic-acid | ZINC000000001067 |
| Bucolome | ZINC000002040305, ZINC000002040306 |
| Bucricaine | ZINC000003876083 |
| Bucromarone | ZINC000004215115 |
| Bucumolol | ZINC000000000100, ZINC000006070757 |
| Budipine | ZINC000001481990 |
| Budralazine | ZINC000011616689 |
| Bufenadrine | ZINC000001481742, ZINC000005439905 |
| Bufetolol | ZINC000000000101, ZINC000001846353, ZINC000001846354, ZINC000001846356 |
| Bufexamac | ZINC000003871797 |
| Bufezolac | ZINC000000001069 |
| Buflomedil | ZINC000000005191 |
| Buformin | ZINC000004097425 |
| Bufrolin | ZINC000004215124 |
| Bufuralol | ZINC000000000102, ZINC000000002269 |
| Bumecaine | ZINC000002019930, ZINC000002019932 |
| Bumepidil | ZINC000000001071 |
| Bumetanide | ZINC000003813061 |
| Bumetrizole | ZINC000000056954 |
| Bunaftine | ZINC000002010639 |
| Bunaprolast | ZINC000001542264 |
| Bunazosin | ZINC000000601249 |
| Bunolol | ZINC000003812876, ZINC000003830339 |
| Buparlisib | ZINC000043154039 |
| Bupicomide | ZINC000001574578 |
| Bupivacaine | ZINC000001530812, ZINC000001530814 |

Table S1. (continued next page)

| Drug name | ZINC ID |
| --- | --- |
| Bupranolol | ZINC000000000106, ZINC000000002276 |
| Bupropion | ZINC000000020228, ZINC000000057206 |
| Buquineran | ZINC000004215135 |
| Buquinolate | ZINC000003983799 |
| Burapitant | ZINC000003990019 |
| Burixafor | ZINC000140585706 |
| Burodiline | ZINC000004215138 |
| Buspirone | ZINC000001530571 |
| Butabarbital | ZINC000000968345, ZINC000003651680 |
| Butacetin | ZINC000000001075 |
| Butaclamol | ZINC000002008410, ZINC000040933021 |
| Butadiazamide | ZINC000002029677 |
| Butalamine | ZINC000002002230 |
| Butallylonal | ZINC000018167404, ZINC000018191874 |
| Butamirate | ZINC000002017415, ZINC000002017416 |
| Butamisole | ZINC000000000109, ZINC000004212236 |
| Butanilicaine | ZINC000001841441 |
| Butanixin | ZINC000004215142 |
| Butanserin | ZINC000002019989 |
| Butantrone | ZINC000000001077 |
| Butaperazine | ZINC000022446639 |
| Butaverine | ZINC000001569382, ZINC000004212244 |
| butazolamide | ZINC000032150186 |
| Butazopyridine | ZINC000015990251, ZINC000100365951, ZINC000253977919 |
| Butenafine | ZINC000001530975 |
| Buterizine | ZINC000022463149 |
| Butetamate | ZINC000001718577, ZINC000002018726 |
| Buthiazide | ZINC000000607750, ZINC000002038967 |
| Butibufen | ZINC000000015537, ZINC000000395681 |
| Butidrine | ZINC000004212253, ZINC000027637698, ZINC000027637704, ZINC000027637710 |
| Butilfenin | ZINC000001844982 |
| Butirosin | ZINC000049776327, ZINC000049776328, ZINC000060184704 |
| Butoconazole | ZINC000001530973, ZINC000001530974 |
| Butocrolol | ZINC000004212261, ZINC000006069570 |
| Butofilolol | ZINC000000000112, ZINC000001846206 |
| Butopiprine | ZINC000056898781, ZINC000056898783 |
| Butoprozine | ZINC000004215163 |
| Butopyrammonium | ZINC000004212269 |
| Butorphanol | ZINC000003812988, ZINC000004626731 |
| Butoxamine | ZINC000000057208, ZINC000000057209, ZINC000000057210, ZINC000000057211 |
| Butoxycaine | ZINC000002038682 |
| Butriptyline | ZINC000001481747, ZINC000001678302 |
| Butylphenamide | ZINC000002041011 |
| Buzepide | ZINC000000607754 |
| C-1311 | ZINC000003825292 |
| Cabergoline | ZINC000001531027, ZINC000003800008, ZINC000003830350, ZINC000003830351, ZINC000003830352, ZINC000011592905, ZINC000011592906, ZINC000011592907 |
| Cabotegravir | ZINC000096927633 |
| Cabozantinib | ZINC000070466416 |
| Cadazolid | ZINC000043195938 |

Table S1. (continued next page)

| Drug name | ZINC ID |
| --- | --- |
| Cadrofloxacin | ZINC000003795937 |
| Cafaminol | ZINC000000001081 |
| Cafedrine | ZINC000001843137, ZINC000001843140, ZINC000001843146, ZINC000004212291 |
| Calcipotriene | ZINC000003921872, ZINC000004654793, ZINC000033938184, ZINC000038599402, ZINC000038791645, ZINC000043773451, ZINC000065731406, ZINC000065731407, ZINC000077285174, ZINC000095936503, ZINC000100489836, ZINC000100517381, ZINC000100517384, ZINC000142740411, ZINC000148917203 |
| Calcobutrol | ZINC000022446966, ZINC000030691571, ZINC000030691574, ZINC000030691579 |
| Caldaret | ZINC000001551732 |
| Caldiamide | ZINC000021297783 |
| Caloxetic-acid | ZINC000022448097, ZINC000022851765 |
| Camazepam | ZINC000000601288, ZINC000002008504 |
| Cambendazole | ZINC000005424098 |
| Camicinal | ZINC000034850365 |
| Camiverine | ZINC000019368425, ZINC000019368427 |
| Camobucol | ZINC000003937466 |
| Camonagrel | ZINC000000000115, ZINC000002016018 |
| Camostat | ZINC000003871842 |
| Camphor | ZINC000000967520 |
| Camylofin | ZINC000019369227, ZINC000019369230 |
| Canagliflozin | ZINC000043207238 |
| Candesartan | ZINC000003782818 |
| Candoxatril | ZINC000100036919 |
| Cannabidiol | ZINC000004097406 |
| Cannabinol | ZINC000001530833 |
| Capecitabine | ZINC000001539037, ZINC000001865093, ZINC000003806413, ZINC000005973243, ZINC000005973244, ZINC000005973245, ZINC000011616162, ZINC000011616163, ZINC000019615653, ZINC000019615657, ZINC000049015568 |
| Capeserod | ZINC000000603773 |
| Capmatinib | ZINC000043195321 |
| Capravirine | ZINC000000538635 |
| Capromorelin | ZINC000004393135, ZINC000011678025, ZINC000011678032 |
| Caproxamine | ZINC000004215179 |
| Captodiame | ZINC000002040210, ZINC000002040211 |
| Carabersat | ZINC000003823825 |
| Caramiphen | ZINC000001847743 |
| Carazolol | ZINC000001567835, ZINC000001999243 |
| Carbadox | ZINC000000156806, ZINC000011678038 |
| Carbamazepine | ZINC000000004785 |
| Carbantel-lauryl | ZINC000030690782 |
| Carbaril | ZINC000000001090 |
| Carbazeran | ZINC000000608235 |
| Carbetapentane | ZINC000003830375 |
| Carbiphene | ZINC000001698104 |
| Carbofenotion | ZINC000000001092 |
| Carboprost | ZINC000016343305 |
| Carbubarb | ZINC000002040108 |
| Carburazepam | ZINC000000000123, ZINC000001846261 |
| Carcainium | ZINC000002040034 |
| Carebastine | ZINC000004215193 |
| Carfentanil | ZINC000004215196 |

Table S1. (continued next page)

| Drug name | ZINC ID |
| --- | --- |
| Cariporide | ZINC000013474734 |
| Cariprazine | ZINC000100153731 |
| Carisbamate | ZINC000030691363 |
| Carisoprodol | ZINC000001530950, ZINC000001530951 |
| Carmantadine | ZINC000004212355, ZINC000005190478 |
| Carmetizide | ZINC000001846402, ZINC000001846403 |
| Carmoterol | ZINC000034853956 |
| Carmoxirole | ZINC000001533114 |
| Carocainide | ZINC000004215199 |
| Caroverine | ZINC000004212360 |
| Caroxazone | ZINC000000001095 |
| Carperidine | ZINC000000001096 |
| Carperone | ZINC000004215204 |
| Carphenazine | ZINC000022446644 |
| Carpindolol | ZINC000000000127, ZINC000003649831 |
| Carpipramine | ZINC000000597537 |
| Carprazidil | ZINC000000001097 |
| Carprofen | ZINC000000020235, ZINC000001869694 |
| Carsatrin | ZINC000022851028, ZINC000022851029 |
| Cartazolate | ZINC000021290032 |
| Carteolol | ZINC000000000128, ZINC000000896463 |
| Carumonam | ZINC000003917496 |
| Carvedilol | ZINC000001530579, ZINC000001530580 |
| Carvotroline | ZINC000029485479 |
| Carzenide | ZINC000000001099 |
| Casokefamide | ZINC000072266935 |
| Casopitant | ZINC000042856596 |
| Catramilast | ZINC000000024222 |
| Cc-401 | ZINC000038836256 |
| Ce-224535 | ZINC000034662892, ZINC000035939968 |
| Cebaracetam | ZINC000001533476, ZINC000002016005 |
| Cebranopadol | ZINC000003950145 |
| Cediranib | ZINC000003948085 |
| Celecoxib | ZINC000002570895 |
| Celgosivir | ZINC000003797478 |
| Celiprolol | ZINC000002001884, ZINC000003813069 |
| Cemadotin | ZINC000003946361 |
| Cenisertib | ZINC000063298059 |
| Centanafadine | ZINC000034986938 |
| Cep-11981 | ZINC000036411318 |
| Cep-1347 | ZINC000003928304 |
| Cep-32496 | ZINC000043207440 |
| Cep-5214 | ZINC000001551940 |
| Cep-7055 | ZINC000003955614 |
| Cerc-301 | ZINC000006716889, ZINC000006716890, ZINC000006716891, ZINC000140559171 |
| Cericlamine | ZINC000000000130, ZINC000003649817 |
| Ceritinib | ZINC000096272772 |
| Cerivastatin | ZINC000011330186 |
| Cerlapirdine | ZINC000034999931 |
| Ceronapril | ZINC000003785696 |

Table S1. (continued next page)

| Drug name | ZINC ID |
| --- | --- |
| Cetamolol | ZINC000000000131, ZINC000001853833 |
| Cetefloxacin | ZINC000003779887 |
| Cetiedil | ZINC000000000132, ZINC000001638266 |
| Cetirizine | ZINC000019364229, ZINC000019364230 |
| Cetoxime | ZINC000004215288 |
| Cetraxate | ZINC000100047606 |
| Cevimeline | ZINC000000008699, ZINC000000010164, ZINC000003792909 |
| Cevipabulin | ZINC000013981125 |
| Chir-265 | ZINC000018710085 |
| Chlophedianol | ZINC000000057253, ZINC000000057254 |
| Chloracyzine | ZINC000004215293 |
| Chlorazanil | ZINC000000001116 |
| Chlorbenzoxamine | ZINC000022462882, ZINC000022588434 |
| Chlorcyclizine | ZINC000019362735, ZINC000019362737 |
| Chlordiazepoxide | ZINC000019632917 |
| Chlordimorine | ZINC000031540242 |
| Chlorfenethazine | ZINC000001568623 |
| Chlorisondamine | ZINC000000608245 |
| Chlormezanone | ZINC000000000133, ZINC000000896468 |
| Chlormidazole | ZINC000000084088 |
| Chloroguanide | ZINC000095452610 |
| Chloroprocaine | ZINC000001530938 |
| Chloropyramine | ZINC000020148995 |
| Chloroquine | ZINC000019144226, ZINC000019144231 |
| Chloroserpidine | ZINC000004212444 |
| Chlorothen | ZINC000000001128 |
| Chlorothiazide | ZINC000003872055 |
| Chlorotrianisene | ZINC000001530598 |
| Chloroxine | ZINC000000001131 |
| Chlorphenesin-carbamate | ZINC000000000136, ZINC000000897317 |
| Chlorpheniramine | ZINC000000113404, ZINC000000113410 |
| Chlorphenoxamine | ZINC000000000137, ZINC000002034754 |
| Chlorproethazine | ZINC000000001135 |
| Chlorproguanil | ZINC000000001136 |
| Chlorpromazine | ZINC000000044027 |
| Chlorpropamide | ZINC000001530599 |
| Chlorprothixene | ZINC000000001137 |
| Chlorpyrifos | ZINC000000608250 |
| Chlorquinaldol | ZINC000000119403 |
| Chlorthalidone | ZINC000000020253, ZINC000000057255 |
| Chlorzoxazone | ZINC000084843283 |
| Choline-alfoscerate | ZINC000001532714, ZINC000001842903 |
| Chromonar | ZINC000004215319 |
| Ci-988 | ZINC000003920670, ZINC000014952320, ZINC000014952325, ZINC000014952331 |
| Cianidanol | ZINC000000119978, ZINC000000119983, ZINC000000119985, ZINC000000119988 |
| Cicarperone | ZINC000031297676 |
| Ciclafrine | ZINC000003649813, ZINC000004626626 |
| Ciclazindol | ZINC000000000142, ZINC000004212464 |
| Cicletanine | ZINC000000002277, ZINC000000009709 |
| Ciclonicate | ZINC000004215324 |

Table S1. (continued next page)

| Drug name | ZINC ID |
| --- | --- |
| Ciclopirox | ZINC000000001145 |
| Ciclopramine | ZINC000004212470, ZINC000004626627 |
| Cicloprofen | ZINC000000000144, ZINC000001566379 |
| Cicloprolol | ZINC000001534254, ZINC000004212476 |
| Ciclosidomine | ZINC000005784106 |
| Ciclotizolam | ZINC000004212479 |
| Cidofovir | ZINC000001530600 |
| Cifenline | ZINC000000000145, ZINC000000002266 |
| Ciglitazone | ZINC000001481754, ZINC000001854671 |
| Ciheptolane | ZINC000003649810, ZINC000004626628 |
| Ciladopa | ZINC000029563177 |
| Cilansetron | ZINC000000004035 |
| Cilazaprilat | ZINC000004212489 |
| Cilazapril | ZINC000001532328, ZINC000003781951, ZINC000003872072, ZINC000011615961, ZINC000011615962 |
| Cilobamine | ZINC000001693021 |
| Cilostamide | ZINC000003872076 |
| Cilostazol | ZINC000001552174 |
| Ciltoprazine | ZINC000004215333 |
| Cilutazoline | ZINC000000001151 |
| Cimemoxin | ZINC000019172733 |
| Cimicoxib | ZINC000001494105 |
| Cinacalcet | ZINC000001550499 |
| Cinalukast | ZINC000003803377 |
| Cinaproxen | ZINC000004215339 |
| Cinchonine | ZINC000003881680 |
| Cinchophen | ZINC000000125047 |
| Cinfenine | ZINC000004215341 |
| Cinitapride | ZINC000003784384, ZINC000005316843 |
| Cinnamedrine | ZINC000000000151, ZINC000001998378, ZINC000002015725, ZINC000005751636 |
| Cinnarizine | ZINC000019632891 |
| Cinnofuradione | ZINC000000000152, ZINC000003649799 |
| Cinoxacin | ZINC000000032350 |
| Cinperene | ZINC000002018994, ZINC000002018996 |
| Cinprazole | ZINC000022463155 |
| Cintazone | ZINC000001680067, ZINC000002038936 |
| Cinuperone | ZINC000031425112 |
| Cioteronel | ZINC000011678358 |
| Cipamfylline | ZINC000003781835 |
| Cipemastat | ZINC000000600699 |
| Ciprafamide | ZINC000004215363 |
| Ciprazafone | ZINC000004215365 |
| Ciprefadol | ZINC000003776671 |
| Ciprofibrate | ZINC000000004942, ZINC000002005072 |
| Ciprofloxacin | ZINC000000020220 |
| Cipropride | ZINC000000607772, ZINC000001846145 |
| Ciproquazone | ZINC000000000154 |
| Ciramadol | ZINC000004212551 |
| Cirazoline | ZINC000000001160 |
| Cisapride | ZINC000003775140, ZINC000003830564, ZINC000004215367 |

Table S1. (continued next page)

| Drug name | ZINC ID |
| --- | --- |
| Cisconazole | ZINC000003776651 |
| Citenamide | ZINC000000001162 |
| Cizolirtine | ZINC000000004037, ZINC000003649791 |
| Clamidoxic-acid | ZINC000000001166 |
| Clamoxyquin | ZINC000001571039 |
| Clanfenur | ZINC000000001167 |
| Clanobutin | ZINC000002034509 |
| Clantifen | ZINC000000001168 |
| Clazolam | ZINC000036387477, ZINC000037246129 |
| Clazolimine | ZINC000000001170 |
| Clazosentan | ZINC000003939238 |
| Clebopride | ZINC000000608261 |
| Clemastine | ZINC000000402830 |
| Clemeprol | ZINC000000000161, ZINC000034220986, ZINC000034220987, ZINC000034220988 |
| Clemizole | ZINC000000057260 |
| Clenbuterol | ZINC000000057261, ZINC000000057262 |
| Clenpirin | ZINC000004474484 |
| Clentiazem | ZINC000000607779, ZINC000001999850, ZINC000011678452, ZINC000011678458 |
| Cletoquine | ZINC000001843038, ZINC000001843040 |
| Clevudine | ZINC000000001484 |
| Clibucaine | ZINC000000000162, ZINC000002020010 |
| Clidafidine | ZINC000013835972 |
| Clidanac | ZINC000000000163, ZINC000003830577 |
| Clidinium | ZINC000000601316, ZINC000003830579 |
| Climazolam | ZINC000000001173 |
| Climiqualine | ZINC000000001174 |
| Clinafloxacin | ZINC000001278767, ZINC000003882444 |
| Clinofibrate | ZINC000003812946, ZINC000003872097, ZINC000003872098 |
| Cliprofen | ZINC000000000165, ZINC000006037080 |
| Cliropamine | ZINC000003649787, ZINC000006071251, ZINC000006071252, ZINC000006071253 |
| Clobazam | ZINC000000001175 |
| Clobenoside | ZINC000004212590, ZINC000031291735, ZINC000031291738, ZINC000038612108 |
| Clobenzepam | ZINC000000001176 |
| Clobenzorex | ZINC000019890801, ZINC000019890803 |
| Clobenztropine | ZINC000100092026, ZINC000100092028 |
| Clobutinol | ZINC000000000166, ZINC000002018980, ZINC000002018981, ZINC000002018982 |
| Clobuzarit | ZINC000000001179 |
| Clocanfamide | ZINC000005736063, ZINC000005752278 |
| Clocapramine | ZINC000000608266 |
| Clociguanil | ZINC000000001180 |
| Clocinizine | ZINC000022462888, ZINC000031298006 |
| Clodacaine | ZINC000019364698 |
| Clodanolene | ZINC000000001181 |
| Clodazon | ZINC000000001182 |
| Clodoxopone | ZINC000004215401 |
| Clofenamic-acid | ZINC000000001183 |
| Clofenamide | ZINC000001482010 |
| Clofenciclan | ZINC000000001185 |
| Clofenetamine | ZINC000001843059, ZINC000001843060 |
| Clofexamide | ZINC000001999309 |

Table S1. (continued next page)

| Drug name | ZINC ID |
| --- | --- |
| Clofibrate | ZINC000000056648 |
| Clofibride | ZINC000003830586 |
| Cloflucarban | ZINC000000001188 |
| Clofoctol | ZINC000002149829 |
| Cloforex | ZINC000000001189 |
| Clofurac | ZINC000000001190 |
| Clomacran | ZINC000000000169, ZINC000002015677 |
| Clometacin | ZINC000004212617 |
| Clomifenoxide | ZINC000003647465 |
| Clomiphene | ZINC000001530601, ZINC000012402836 |
| Clomipramine | ZINC000000020248 |
| Clonazepam | ZINC000003813003 |
| Clonazoline | ZINC000000001193 |
| Clonidine | ZINC000000896484 |
| Clonitazene | ZINC000004215423 |
| Clonixeril | ZINC000000000171, ZINC000001576406 |
| Clonixin | ZINC000000001195 |
| Clopamide | ZINC000000057235, ZINC000000057236, ZINC000018847050 |
| Clopenthixol | ZINC000000601293, ZINC000031483558 |
| Cloperastine | ZINC000000057251, ZINC000000057252 |
| Cloperidone | ZINC000004215426 |
| Clopidogrel | ZINC000034781704 |
| Clopimozide | ZINC000004215428 |
| Clopipazan | ZINC000000001197 |
| Clopirac | ZINC000000001198 |
| Cloquinozine | ZINC000000000172, ZINC000003608436, ZINC000031541365, ZINC000031541373 |
| Cloranolol | ZINC000000000174, ZINC000002019902 |
| Clorazepic-acid | ZINC000028973441, ZINC000028973446 |
| Clorexolone | ZINC000000001199 |
| Clorfenvinfos | ZINC000013531994 |
| Cloricromen | ZINC000000576842 |
| Cloridarol | ZINC000000000176, ZINC000001999569 |
| Clorindione | ZINC000100003913 |
| Clormecaine | ZINC000000001202 |
| Cloroperone | ZINC000004215442 |
| Cloroqualone | ZINC000000001203 |
| Clorotepine | ZINC000019368911, ZINC000022032760 |
| Clorprenaline | ZINC000000000178, ZINC000001576350 |
| Clorsulon | ZINC000002001200 |
| Closiramine | ZINC000000000179, ZINC000004212654 |
| Clothiapine | ZINC000026185346 |
| Clothixamide | ZINC000000608277 |
| Clotiazepam | ZINC000000001207 |
| Clovoxamine | ZINC000004215447 |
| Cloxacepride | ZINC000004212663 |
| Cloxazolam | ZINC000040017475, ZINC000040017476 |
| Cloximate | ZINC000038807192 |
| Cloxypendyl | ZINC000022463167 |
| Clozapine | ZINC000019796155 |
| Cns-5161 | ZINC000005423063 |

Table S1. (continued next page)

| Drug name | ZINC ID |
| --- | --- |
| Cobicistat | ZINC000085537014 |
| Cocaine | ZINC000003875336 |
| Cocarboxylase | ZINC000008215517 |
| Codeine | ZINC000003806721 |
| Codoxime | ZINC000004215450 |
| Cogazocine | ZINC000001481760 |
| Colchicine | ZINC000000607790, ZINC000000621853 |
| Colfenamate | ZINC000000001214 |
| Colterol | ZINC000000000184, ZINC000002015274 |
| Coluracetam | ZINC000000004939 |
| Conivaptan | ZINC000012503187 |
| Copanlisib | ZINC000068247389 |
| Cort-108297 | ZINC000029128012, ZINC000141514903 |
| Cortisuzol | ZINC000004215464 |
| Cotinine | ZINC000000402766 |
| Coumaphos | ZINC000000608284 |
| Coumazoline | ZINC000000001218 |
| Cp-724714 | ZINC000003817105, ZINC000018710086 |
| Crenolanib | ZINC000003820043 |
| Cresatin | ZINC000004284473 |
| Cridanimod | ZINC000003878688 |
| Crilvastatin | ZINC000003775270, ZINC000003780188, ZINC000005735917, ZINC000005752118 |
| Crisnatol | ZINC000000005042 |
| Crizotinib | ZINC000035902488, ZINC000035902489 |
| Croconazole | ZINC000000001223 |
| Cromoglicate-lisetil | ZINC000003914388, ZINC000004215474 |
| Cromolyn | ZINC000001530788 |
| Crufomate | ZINC000001557007, ZINC000002040871 |
| Crystal-violet | ZINC000013763987 |
| Cyc-116 | ZINC000003950132 |
| Cyclandelate | ZINC000000405331, ZINC000008551997, ZINC000008551998, ZINC000008551999 |
| Cyclarbamate | ZINC000000608287 |
| Cyclazocine | ZINC000003830613 |
| Cyclazodone | ZINC000000000190, ZINC000002019160 |
| Cyclexanone | ZINC000038600697, ZINC000038600699 |
| Cyclindole | ZINC000000000192, ZINC000002010690 |
| Cycliramine | ZINC000000001228 |
| Cyclizine | ZINC000019156872 |
| Cyclobendazole | ZINC000005387001 |
| Cyclobenzaprine | ZINC000000968263 |
| Cyclocumarol | ZINC000001481765, ZINC000002036735, ZINC000002036736, ZINC000004212731 |
| Cyclodrine | ZINC000001641450, ZINC000003630877 |
| Cyclofenil | ZINC000000608288 |
| Cycloguanil | ZINC000000001233 |
| Cycloheximide | ZINC000003872170 |
| Cyclomethycaine | ZINC000004212735, ZINC000006037520 |
| Cyclopentamine | ZINC000001481766, ZINC000002041152 |
| Cyclopenthiazide | ZINC000000601312, ZINC000003647770 |
| Cyclopentolate | ZINC000000000196, ZINC000000155531 |
| Cyclophenazine | ZINC000022463171 |

Table S1. (continued next page)

| Drug name | ZINC ID |
| --- | --- |
| Cycrimine | ZINC000000000199, ZINC000002041282 |
| Cyheptamide | ZINC000000001236 |
| Cyheptropine | ZINC000100372252 |
| Cypenamine | ZINC000002037166, ZINC000002037167, ZINC000002037168, ZINC000005934445 |
| Cyprodenate | ZINC000001842986 |
| Cyproheptadine | ZINC000000968264 |
| Cyprolidol | ZINC000001752005, ZINC000004822836, ZINC000012875914, ZINC000017354679 |
| Cyproquinate | ZINC000004215497 |
| Cyproximide | ZINC000002018728, ZINC000002018729 |
| Cyromazine | ZINC000000001239 |
| Cytarabine | ZINC000000895248, ZINC000001078621, ZINC000002583632, ZINC000003795098, ZINC000003830623, ZINC000003830624, ZINC000003830625, ZINC000003830626, ZINC000003978018, ZINC000006091575, ZINC000006524892, ZINC000012336757, ZINC000016969357 |
| Cythioate | ZINC000001569224 |
| Dabelotine | ZINC000003630870, ZINC000003630873 |
| Dabigatran | ZINC000001910616 |
| Dabrafenib | ZINC000068153186 |
| Dabuzalgron | ZINC000049952320 |
| Dacarbazine | ZINC000018099446, ZINC000100019007, ZINC000254748682 |
| Dacemazine | ZINC000000000200 |
| Daclatasvir | ZINC000068204830 |
| Dacopafant | ZINC000000004355, ZINC000003799334 |
| Dacuronium | ZINC000004212779 |
| Dagrocorat | ZINC000103297733 |
| Dalbraminol | ZINC000003630864, ZINC000003630866 |
| Dalcetrapib | ZINC000003976476 |
| Dalcotidine | ZINC000001889603 |
| Daledalin | ZINC000002015297, ZINC000002015298 |
| Daltroban | ZINC000000608296 |
| Dalvastatin | ZINC000000597504, ZINC000002020500 |
| Damotepine | ZINC000000001243 |
| Danegaptide | ZINC000040836075 |
| Daniquidone | ZINC000000003827 |
| Danitracen | ZINC000001481770 |
| Danofloxacin | ZINC000000607798 |
| Danusertib | ZINC000006718723 |
| Dapagliflozin | ZINC000003819138 |
| Dapiprazole | ZINC000000001246 |
| Dapitant | ZINC000003932681 |
| Dapoxetine | ZINC000001482019 |
| Daprodustat | ZINC000231226004 |
| Darapladib | ZINC000003842798 |
| Darglitazone | ZINC000001492464, ZINC000001851105 |
| Darifenacin | ZINC000001996117 |
| Darusentan | ZINC000003826221 |
| Dasabuvir | ZINC000095616937 |
| Dasantafil | ZINC000003839138 |
| Dasatinib | ZINC000003986735 |
| Dasotraline | ZINC000002510873 |
| Datelliptium | ZINC000000608300 |

Table S1. (continued next page)

| Drug name | ZINC ID |
| --- | --- |
| Davasaicin | ZINC000002009579 |
| Dazadrol | ZINC000000000201, ZINC000011616987 |
| Dazepinil | ZINC000000000202, ZINC000001854320 |
| Dazidamine | ZINC000003630852 |
| Dazmegrel | ZINC000035877948 |
| Dazolicine | ZINC000000001250 |
| Dazopride | ZINC000019168101 |
| Dazoquinast | ZINC000001999559 |
| Dazoxiben | ZINC000169365016 |
| Deboxamet | ZINC000000001251 |
| Debrisoquin | ZINC000003594299 |
| Decernotinib | ZINC000096941867 |
| Decitabine | ZINC000000001253, ZINC000004533818, ZINC000008627921, ZINC000008627922, ZINC000011678823, ZINC000011678826, ZINC000016929327, ZINC000017027370 |
| Declenperone | ZINC000004215520 |
| Declopramide | ZINC000001546399 |
| Decloxizine | ZINC000019364667 |
| Defactinib | ZINC000103297739 |
| Deferasirox | ZINC000001481815 |
| Deferitrin | ZINC000013645794 |
| Delafloxacin | ZINC000003827556 |
| Delamanid | ZINC000043100810 |
| Delapril | ZINC000003794599 |
| Delavirdine | ZINC000018516586 |
| Delequamine | ZINC000000597368 |
| Delfantrine | ZINC000000001255 |
| Delfaprazine | ZINC000000004054 |
| Delmetacin | ZINC000000001256 |
| Delmopinol | ZINC000031283194, ZINC000031283197 |
| Delorazepam | ZINC000001255325 |
| Delucemine | ZINC000002009020 |
| Demecolcine | ZINC000003872131, ZINC000003872132 |
| Demelverine | ZINC000008462709 |
| Demexiptiline | ZINC000001542913 |
| Democonazole | ZINC000001846112 |
| Demoxepam | ZINC000001677763 |
| Denatonium | ZINC000001601437 |
| Denaverine | ZINC000002003679 |
| Denibulin | ZINC000011726215 |
| Denipride | ZINC000005423187, ZINC000005423188 |
| Denotivir | ZINC000000608304 |
| Denpidazone | ZINC000100375729 |
| Denzimol | ZINC000000000207, ZINC000013209898 |
| Depramine | ZINC000000001260 |
| Deracoxib | ZINC000000607803 |
| Deramciclane | ZINC000003643198 |
| Derenofylline | ZINC000044672349, ZINC000100002114, ZINC000252673365 |
| Deriglidole | ZINC000000004503, ZINC000002015994 |
| Derquantel | ZINC000056898856 |
| Desalkylflurazepam | ZINC000001433338 |

Table S1. (continued next page)

| Drug name | ZINC ID |
| --- | --- |
| Desciclovir | ZINC000002021429 |
| Deserpidine | ZINC000003830660, ZINC000003830661, ZINC000003830662, ZINC000003830663, ZINC000004097186, ZINC000105090645, ZINC000257400370, ZINC000257400371, ZINC000257400372 |
| Desipramine | ZINC000001530611 |
| Desloratadine | ZINC000000001261 |
| Desmethylmoramide | ZINC000031560325 |
| Desocriptine | ZINC000004212849 |
| Desomorphine | ZINC000004215567 |
| Desvenlafaxine | ZINC000000009342, ZINC000002525885 |
| Detanosal | ZINC000001733655 |
| Detiviciclovir | ZINC000033639600 |
| Detomidine | ZINC000005116305 |
| Devazepide | ZINC000001847292 |
| Dexamisole | ZINC000000119839, ZINC000000119842, ZINC000135897132, ZINC000136472050 |
| Dexefaroxan | ZINC000000002748, ZINC000001842775 |
| Dexelvucitabine | ZINC000000002300 |
| Dexetimide | ZINC000006927490, ZINC000006927491 |
| Dexibuprofen | ZINC000000002647, ZINC000000113398 |
| Dexindoprofen | ZINC000000000391, ZINC000000156823 |
| Dexketoprofen | ZINC000000002272, ZINC000000005560 |
| Dexlofexidine | ZINC000000001266, ZINC000000005626 |
| Dexloxiglumide | ZINC000003789440, ZINC000003801027 |
| Dexnafenodone | ZINC000001481882, ZINC000001482031 |
| Dexsecoverine | ZINC000004212898, ZINC000004214305 |
| Dextofisopam | ZINC000000608106, ZINC000003831552 |
| Dextrofemine | ZINC000000000692, ZINC000001842961, ZINC000001842963, ZINC000001842981 |
| Dextromoramide | ZINC000004215575, ZINC000205538069 |
| Dezaguanine | ZINC000017061339 |
| Dezinamide | ZINC000000012970 |
| Dezocine | ZINC000003830683 |
| Diacetazotol | ZINC000016362544, ZINC000100069197, ZINC000254869689 |
| Diacetolol | ZINC000000000211, ZINC000001573408 |
| Diacetylmorphine | ZINC000004097183 |
| Diamfenetide | ZINC000001565851 |
| Diamocaine | ZINC000004215585 |
| Diampromide | ZINC000001850015, ZINC000001850017 |
| Diamthazole | ZINC000000001271 |
| Dianicline | ZINC000003966685 |
| Diapamide | ZINC000000001272 |
| Diaplasinin | ZINC000001554077 |
| Diarbarone | ZINC000031484950 |
| Diaveridine | ZINC000000024946 |
| Diazepam | ZINC000000006427 |
| Diazolidinylurea | ZINC000001850155, ZINC000001850156 |
| Diazoxide | ZINC000003872277 |
| Dibekacin | ZINC000008214383 |
| Dibemethine | ZINC000001234761 |
| Dibenzepin | ZINC000000001275 |
| dibromol | ZINC000001764317 |

Table S1. (continued next page)

| Drug name | ZINC ID |
| --- | --- |
| Dibrompropamidine | ZINC000001665651 |
| Dibromsalan | ZINC000000608319 |
| Dibucaine | ZINC000001530939 |
| Dibunic-acid | ZINC000004214320 |
| Dibupyrone | ZINC000003623579 |
| Dibusadol | ZINC000004215615 |
| Dibutoline | ZINC000001675024 |
| Dibutyl-phthalate | ZINC000001693431 |
| Dicarbine | ZINC000000001161, ZINC000000518701, ZINC000001800183 |
| Dicarfen | ZINC000001692923 |
| Dichlormezanone | ZINC000000000212, ZINC000001999513 |
| Dichlorophen | ZINC000000056435 |
| Dichlorphenamide | ZINC000000896918 |
| Diclofenac | ZINC000000001281 |
| Diclofensine | ZINC000029230271, ZINC000029230278 |
| Diclofurime | ZINC000003623574 |
| Diclometide | ZINC000003623573 |
| Diclonixin | ZINC000000001282 |
| Dicolinium | ZINC000001999369, ZINC000001999370, ZINC000001999371, ZINC000001999372 |
| Dicyclomine | ZINC000001530613 |
| Didanosine | ZINC000013597823, ZINC000013827997, ZINC000016025885, ZINC000018068892 |
| Dienestrol | ZINC000000001283 |
| Diethazine | ZINC000000001286 |
| Diethylaminoethoxyhexestrol | ZINC000031291809, ZINC000031291811, ZINC000031291814 |
| Diethylaminoethyl-diphenylhydroxypropionate | ZINC000005847577 |
| Diethylcarbamazine | ZINC000000001288 |
| Diethylpropion | ZINC000000000215, ZINC000002847375 |
| Diethylstilbestrol-diphosphate | ZINC000001531011, ZINC000001531012 |
| Diethylstilbestrol-dipropionate | ZINC000000608323, ZINC000013704474 |
| Dietifen | ZINC000000001292 |
| Difebarbamate | ZINC000004212927, ZINC000040163079 |
| Difemerine | ZINC000044710394 |
| Difemetorex | ZINC000001481782, ZINC000002020022 |
| Difenamizole | ZINC000000000217, ZINC000002019965 |
| Difenpiramide | ZINC000000001293 |
| Difeterol | ZINC000003623570, ZINC000004212934, ZINC000005763217, ZINC000005763250 |
| Diflomotecan | ZINC000001489193 |
| Difloxacin | ZINC000004099034 |
| Difluanine | ZINC000019364169 |
| Diflumidone | ZINC000000001294 |
| Diflunisal | ZINC000000020243 |
| Diftalone | ZINC000000001296 |
| Dihydrocodeine | ZINC000004215736 |
| Dihydroergocristine | ZINC000003947494, ZINC000003947495, ZINC000003947496, ZINC000003947497, ZINC000003995616, ZINC000095862766 |
| Dihydroergocryptine | ZINC000003929793 |

Table S1. (continued next page)

| Drug name | ZINC ID |
| --- | --- |
| Dihydroergotamine | ZINC000003978005 |
| Diisopromine | ZINC000001481784 |
| Dilazep | ZINC000022454221 |
| Dilmapimod | ZINC000034997404 |
| Diltiazem | ZINC000000621893 |
| Dimabefylline | ZINC000000001301 |
| Dimecolonium | ZINC000001999354, ZINC000001999355, ZINC000001999356, ZINC000001999357 |
| Dimefadane | ZINC000002037189, ZINC000002037190, ZINC000002037191, ZINC000005689252 |
| Dimefline | ZINC000000000223 |
| Dimelazine | ZINC000000000224, ZINC000003623563 |
| Dimemorfan | ZINC000004215661 |
| Dimenoxadol | ZINC000001608965 |
| Dimepheptanol | ZINC000001587666, ZINC000001587667 |
| Dimetacrine | ZINC000001482035 |
| Dimethazan | ZINC000000089652 |
| Dimethindene | ZINC000001481789, ZINC000001482162 |
| Dimethisoquin | ZINC000001671507 |
| Dimetholizine | ZINC000001664858 |
| Dimethoxanate | ZINC000001678301 |
| Dimethylaminopropionylphenothiazine | ZINC000006020070, ZINC000006032472 |
| Dimetipirium | ZINC000000607825, ZINC000004212990 |
| Dimetofrine | ZINC000000000231, ZINC000002015427 |
| Dimetridazole | ZINC000000001307 |
| Diminazene | ZINC000003830706 |
| Dimiracetam | ZINC000000004308, ZINC000002015985 |
| Dimorpholamine | ZINC000003872313 |
| Dimoxyline | ZINC000000608327 |
| Dimpylate | ZINC000000001309 |
| Dinaciclib | ZINC000034894449 |
| Dinazafone | ZINC000001846096 |
| Diniprofylline | ZINC000001999280, ZINC000031298029 |
| Dinitolmide | ZINC000002040950 |
| Dinsed | ZINC000004215678 |
| Diosmetin | ZINC000005733652 |
| Diosmin | ZINC000004098512 |
| Dioxadilol | ZINC000003623549, ZINC000003623551, ZINC000003623553, ZINC000003623554 |
| Dioxadrol | ZINC000003080604, ZINC000003080605, ZINC000008143662, ZINC000008659933 |
| Dioxaphetyl-butyrate | ZINC000030612463 |
| Dipenine | ZINC000001843062 |
| Diperodon | ZINC000004213008, ZINC000004245652 |
| Diphemanil | ZINC000001482039 |
| Diphenadione | ZINC000100044649 |
| Diphenan | ZINC000000001314 |
| Diphenhydramine | ZINC000000020244 |
| Diphenidol | ZINC000000968266 |
| Diphenylpiperidinomethyldioxolan | ZINC000003620841, ZINC000003620843 |
| Diphenylpyraline | ZINC000000056643 |
| Dipiproverine | ZINC000000000233, ZINC000001842743 |

Table S1. (continued next page)

| Drug name | ZINC ID |
| --- | --- |
| Dipivefrin | ZINC000000896507, ZINC000003872317 |
| Diprafenone | ZINC000001999478, ZINC000001999479 |
| Diprofene | ZINC000002038310 |
| Diprogulic | ZINC000001825020 |
| Diproqualone | ZINC000000000234, ZINC000001995496 |
| Diproxadol | ZINC000000000235, ZINC000004213022, ZINC000005735517, ZINC000005751793 |
| Dipyridamole | ZINC000000643046 |
| Dipyrocetyl | ZINC000000001316 |
| Dipyrone | ZINC000001782155 |
| Dirlotapide | ZINC000003988502 |
| Disobutamide | ZINC000004213023, ZINC000005442039 |
| Disofenin | ZINC000000001317 |
| Disogluside | ZINC000008214547 |
| Disopyramide | ZINC000001530617, ZINC000001530618 |
| Disulergine | ZINC000004215704 |
| Disulfamide | ZINC000001482043 |
| Disuprazole | ZINC000004633595, ZINC000004633596 |
| Ditazole | ZINC000000001320 |
| Ditercalinium | ZINC000004215707 |
| Ditolamide | ZINC000000001323 |
| Divabuterol | ZINC000003623546, ZINC000004213026 |
| Divaplon | ZINC000000001325 |
| Dixyrazine | ZINC000022453350, ZINC000022593695 |
| Dizatrifone | ZINC000000607830 |
| Dizocilpine | ZINC000025757829 |
| Dobupride | ZINC000005514291 |
| Docetaxel | ZINC000085537053 |
| Doconazole | ZINC000001567316, ZINC000013207499 |
| Dofequidar | ZINC000013652236, ZINC000026180140 |
| Dofetilide | ZINC000000596731 |
| Doliracetam | ZINC000003623544, ZINC000100372235 |
| Dolutegravir | ZINC000058581064 |
| Domazoline | ZINC000000001327 |
| Domitroban | ZINC000013822279 |
| Domoxin | ZINC000019168753, ZINC000019168756 |
| Domperidone | ZINC000004175569 |
| Donepezil | ZINC000000597013, ZINC000000897251 |
| Doqualast | ZINC000000004454 |
| Doramapimod | ZINC000024044436 |
| Doranidazole | ZINC000001493422 |
| Dorastine | ZINC000030690895 |
| Doretinel | ZINC000003785287, ZINC000011679570 |
| Dorzolamide | ZINC000001530621 |
| Dotarizine | ZINC000022058279 |
| Dotefonium | ZINC000000607834, ZINC000001842929 |
| Dothiepin | ZINC000000001329, ZINC000000020249 |
| Dovitinib | ZINC000003816310 |
| Doxaminol | ZINC000031297689, ZINC000031297691 |
| Doxapram | ZINC000019632668, ZINC000019632670 |
| Doxazosin | ZINC000094566092, ZINC000094566093 |

Table S1. (continued next page)

| Drug name | ZINC ID |
| --- | --- |
| Doxefazepam | ZINC000000000240, ZINC000003830724 |
| Doxenitoin | ZINC000026660333 |
| Doxepin | ZINC000000001148, ZINC000000001331 |
| Doxifluridine | ZINC000001319177 |
| Doxofylline | ZINC000000003837 |
| Doxpicomine | ZINC000000000241, ZINC000001575235 |
| Doxylamine | ZINC000000000242, ZINC000007997952 |
| Draflazine | ZINC000022447482, ZINC000022851754 |
| Draquinolol | ZINC000004213051, ZINC000034167971 |
| Drazidox | ZINC000002570827 |
| Dribendazole | ZINC000005997168 |
| Drinabant | ZINC000059299699 |
| Drobuline | ZINC000000000243, ZINC000005442097 |
| Droclidinium | ZINC000000607839, ZINC000001842894, ZINC000001842896, ZINC000001842900 |
| Drofenine | ZINC000001675361, ZINC000002003702 |
| Droloxifene | ZINC000001585847 |
| Dronabinol | ZINC000001530625, ZINC000002034208, ZINC000002039624, ZINC000002039625 |
| Dronedarone | ZINC000049933061 |
| Droperidol | ZINC000019796080 |
| Droprenilamine | ZINC000008143686, ZINC000011679611 |
| Drotebanol | ZINC000004213060 |
| Droxacin | ZINC000000001337 |
| Droxicainide | ZINC000000000245, ZINC000004213063 |
| Droxicam | ZINC000000597502 |
| Droxinavir | ZINC000004213065 |
| Droxypropine | ZINC000003623455 |
| Dulofibrate | ZINC000000001338 |
| Duloxetine | ZINC000001536779, ZINC000001536780 |
| Dulozafone | ZINC000001999563 |
| Duometacin | ZINC000004213067 |
| Duoperone | ZINC000004215745 |
| Dusquetide | ZINC000205877056 |
| Duvelisib | ZINC000088346058 |
| Dyclonine | ZINC000001530940 |
| Dyphylline | ZINC000000057146, ZINC000000057147 |
| Ebalzotan | ZINC000003623452 |
| Ebastine | ZINC000003781952 |
| Eberconazole | ZINC000003783800, ZINC000005117478 |
| Ebrotidine | ZINC000003952167 |
| Ecenofloxacin | ZINC000031495028 |
| Ecipramidil | ZINC000004213069, ZINC000031561757 |
| Eclanamine | ZINC000000001339, ZINC000001842150, ZINC000001842151, ZINC000001842152 |
| Eclazolast | ZINC000001853408 |
| Ecopipam | ZINC000000003897 |
| Ecopladib | ZINC000085536974 |
| Edaglitazone | ZINC000001483899 |
| Edaravone | ZINC000100006441 |
| Edatrexate | ZINC000001618702, ZINC000001618703 |
| Edetol | ZINC000019363525, ZINC000019942898, ZINC000022583252 |
| Edifolone | ZINC000004215756 |

Table S1. (continued next page)

| Drug name | ZINC ID |
| --- | --- |
| Edivoxetine | ZINC000038342901 |
| Edonentan | ZINC000001481808 |
| Edotecarin | ZINC000003946372 |
| Edoxaban | ZINC000043200832 |
| Edoxudine | ZINC000003956771 |
| Edronocaine | ZINC000001545461 |
| Efaproxiral | ZINC000001481819 |
| Efatutazone | ZINC000033975064, ZINC000033975065 |
| Efetozole | ZINC000013281814, ZINC000017022433 |
| Efinaconazole | ZINC000000006251 |
| Efipladib | ZINC000085536943 |
| Efletirizine | ZINC000019367813 |
| Efloxate | ZINC000000001342 |
| Egualen | ZINC000002016044 |
| Elagolix | ZINC000049888891 |
| Elamipretide | ZINC000043130902 |
| Elantrine | ZINC000001482052 |
| Elarofiban | ZINC000003828539 |
| Elbanizine | ZINC000031561759 |
| Elbasvir | ZINC000150588351 |
| Eleclazine | ZINC000206191652 |
| Eletriptan | ZINC000003823475 |
| Elfazepam | ZINC000000608340 |
| Elinafide | ZINC000003811313 |
| Elinogrel | ZINC000043153259 |
| Eliprodil | ZINC000000004041, ZINC000001851251 |
| Elliptinium | ZINC000000001345 |
| Elobixibat | ZINC000003976764 |
| Elomotecan | ZINC000003921664 |
| Elopiprazole | ZINC000053098495 |
| Elpetrigine | ZINC000000009967 |
| Elsibucol | ZINC000003937468 |
| Eltenac | ZINC000000001346 |
| Eltoprazine | ZINC000000001347 |
| Eluxadoline | ZINC000014210876 |
| Elvitegravir | ZINC000013682481 |
| Elziverine | ZINC000072266916 |
| Emapunil | ZINC000000602486 |
| Embramine | ZINC000000000246, ZINC000002019398 |
| Embusartan | ZINC000001539013 |
| Embutramide | ZINC000002018676 |
| Emedastine | ZINC000001530912 |
| Emepronium | ZINC000001481836, ZINC000001843042 |
| Emiglitate | ZINC000004215778 |
| Emivirine | ZINC000001536588 |
| Emixustat | ZINC000059126886 |
| Emorfazone | ZINC000000001349 |
| Empagliflozin | ZINC000036520252 |
| Emtricitabine | ZINC000000005472, ZINC000003629271, ZINC000006524226, ZINC000011616147 |
| Enadoline | ZINC000001914437 |

Table S1. (continued next page)

| Drug name | ZINC ID |
| --- | --- |
| Enalaprilat | ZINC000003812851 |
| Enalapril | ZINC000003791297 |
| Enalkiren | ZINC000003920420 |
| Enasidenib | ZINC000222731806 |
| Encainide | ZINC000000607849, ZINC000004213101 |
| Encaleret | ZINC000066097848 |
| Encenicline | ZINC000095579362 |
| Enciprazine | ZINC000021982927, ZINC000021982930 |
| Encorafenib | ZINC000068249103 |
| Encyprate | ZINC000000056466 |
| Endixaprine | ZINC000000001351 |
| Endomide | ZINC000004215790 |
| Endralazine | ZINC000011679779 |
| Enfenamic-acid | ZINC000000001355 |
| Englitazone | ZINC000005933614, ZINC000005933617, ZINC000005933664, ZINC000005933666 |
| Eniclobrate | ZINC000001573374, ZINC000005316998 |
| Enilconazole | ZINC000001532199, ZINC000001532200 |
| Enilospirone | ZINC000004213116, ZINC000034150724 |
| Eniporide | ZINC000013555902 |
| Enmd-2076 | ZINC000034885047, ZINC000060326209 |
| Enofelast | ZINC000000010998 |
| Enoxacin | ZINC000019594549 |
| Enoxamast | ZINC000003623428 |
| Enoximone | ZINC000009225358 |
| Enpiprazole | ZINC000000001356 |
| Enpiroline | ZINC000001568034, ZINC000001568035, ZINC000001568036, ZINC000004628938 |
| Enprazepine | ZINC000001482055 |
| Enprofylline | ZINC000000403567 |
| Enrofloxacin | ZINC000000597112 |
| Ensaculin | ZINC000001540003 |
| Ensulizole | ZINC000006467621 |
| Entecavir | ZINC000000005235, ZINC000003802690, ZINC000005157450, ZINC000011679834, ZINC000011679840, ZINC000014768473, ZINC000028637466, ZINC000042689357 |
| Entinostat | ZINC000001488870 |
| Entospletinib | ZINC000098208742 |
| Entrectinib | ZINC000043204146 |
| Enviradene | ZINC000006485600 |
| Epacadostat | ZINC000113208009 |
| Epelsiban | ZINC000084727380 |
| Eperezolid | ZINC000003813328 |
| Eperisone | ZINC000000000250, ZINC000001846199 |
| Epervudine | ZINC000003795960 |
| Epetirimod | ZINC000030691782 |
| Epicainide | ZINC000000000251, ZINC000000900569 |
| Epicriptine | ZINC000004215812 |
| Epinastine | ZINC000000006157, ZINC000001999487 |
| Epirizole | ZINC000000057412 |
| Epiroprim | ZINC000000598590 |
| Epithiazide | ZINC000001700363, ZINC000002039262 |
| Eplivanserin | ZINC000001886642 |

Table S1. (continued next page)

| Drug name | ZINC ID |
| --- | --- |
| Eprazinone | ZINC000019362721, ZINC000019362722, ZINC000022033872, ZINC000022033882 |
| Eprobemide | ZINC000019593518 |
| Eprotirome | ZINC000001494227 |
| Eprovafen | ZINC000003623418 |
| Eproxindine | ZINC000002021768, ZINC000002021769 |
| Eprozinol | ZINC000019367106, ZINC000022027602, ZINC000022034312, ZINC000022462965 |
| Epsiprantel | ZINC000000000253, ZINC000001842759 |
| Eptapirone | ZINC000002003628 |
| Eptazocine | ZINC000001846076 |
| Equilin | ZINC000100031739 |
| Equol | ZINC000000388661 |
| Erbulozole | ZINC000004213144, ZINC000011616901 |
| Erdosteine | ZINC000003824065, ZINC000005191096 |
| Ergonovine | ZINC000053174604 |
| Ergotamine | ZINC000052955754 |
| Eribaxaban | ZINC000003816482 |
| Erismodegib | ZINC000068202099 |
| Erizepine | ZINC000001482057 |
| Erlotinib | ZINC000001546066 |
| Erocainide | ZINC000004213147 |
| Ersentilide | ZINC000002022743, ZINC000004633608 |
| Erteberel | ZINC000012353762 |
| Ertiprotafib | ZINC000001547338 |
| Ertugliflozin | ZINC000068197809 |
| Erythromycin | ZINC000085534336 |
| Esafloxacin | ZINC000029333288, ZINC000029333292 |
| Esaprazole | ZINC000019737386 |
| Esculamine | ZINC000000186268 |
| Esculin | ZINC000003860441 |
| Eseridine | ZINC000000898143 |
| Esflurbiprofen | ZINC000000000323, ZINC000000008667 |
| Eslicarbazepine-acetate | ZINC000000007295, ZINC000000023896 |
| Eslicarbazepine | ZINC000000896937, ZINC000000896938 |
| Esmolol | ZINC000000000257, ZINC000000896523 |
| Esonarimod | ZINC000001533116, ZINC000004654791 |
| Esproquin | ZINC000001752012, ZINC000004213151 |
| Estazolam | ZINC000000001370 |
| Estriol | ZINC000000008866, ZINC000003881360, ZINC000012402601 |
| Estrone | ZINC000013509425 |
| Esuprone | ZINC000001537679 |
| eszopiclone | ZINC000169686197 |
| Etabenzarone | ZINC000004215843 |
| Etacepride | ZINC000004213154, ZINC000004626639 |
| Etafedrine | ZINC000000000261, ZINC000011679991 |
| Etafenone | ZINC000000001376 |
| Etalocib | ZINC000003930629 |
| Etamiphyllin | ZINC000000001377 |
| Etanidazole | ZINC000001873938 |
| Etanterol | ZINC000000000263, ZINC000003623387, ZINC000003623390, ZINC000003623392 |
| Etaqualone | ZINC000000001378 |

Table S1. (continued next page)

| Drug name | ZINC ID |
| --- | --- |
| Etarotene | ZINC000084758242 |
| Etasuline | ZINC000000000264, ZINC000003623386 |
| Etazepine | ZINC000000000265, ZINC000002019984 |
| Etebenecid | ZINC000000001380 |
| Eterobarb | ZINC000002986592 |
| Etersalate | ZINC000002032151 |
| Ethacizine | ZINC000004182572 |
| Ethacridine | ZINC000018107429 |
| Ethaverine | ZINC000000608359 |
| Ethiazide | ZINC000001481839, ZINC000002039215 |
| Ethoheptazine | ZINC000000000268, ZINC000001593302 |
| Ethomoxane | ZINC000004213170, ZINC000005647473 |
| Ethonam | ZINC000000000270, ZINC000002018949 |
| Ethopabate | ZINC000000001387 |
| Ethopropazine | ZINC000000004227, ZINC000000056651 |
| Ethosuximide | ZINC000001530805, ZINC000001530806 |
| Ethotoin | ZINC000000000271, ZINC000000897291 |
| Ethoxazene | ZINC000004215852 |
| Ethoxzolamide | ZINC000000056721 |
| Ethybenztropine | ZINC000100203405 |
| Ethyl-biscoumacetate | ZINC000000608360 |
| Ethyl-carfluzepate | ZINC000000607856, ZINC000001846187 |
| Ethyl-dibunate | ZINC000002037216 |
| Ethyl-dirazepate | ZINC000000607858, ZINC000003623382 |
| Ethyl-loflazepate | ZINC000000601273, ZINC000002011625 |
| Ethyl-piperidinoacetylaminobenzoate | ZINC000000200489 |
| Etibendazole | ZINC000005386462 |
| Eticlopride | ZINC000000001395 |
| Eticyclidine | ZINC000001482061 |
| Etidocaine | ZINC000000000276, ZINC000000896998 |
| Etifelmine | ZINC000001482062 |
| Etifenin | ZINC000000001396 |
| Etifoxine | ZINC000000000277, ZINC000002016093 |
| Etilefrine-pivalate | ZINC000004213185, ZINC000005853120 |
| Etimicin | ZINC000100015274, ZINC000100735707, ZINC000103650285, ZINC000255991341, ZINC000255991342, ZINC000255991343, ZINC000255991344 |
| Etipirium | ZINC000000001399 |
| Etiproston | ZINC000030691625 |
| Etisazole | ZINC000000001401 |
| Etisulergine | ZINC000004215916 |
| Etizolam | ZINC000000001402 |
| Etofenamate | ZINC000002034516 |
| Etofenprox | ZINC000002558051 |
| Etofibrate | ZINC000002019929 |
| Etofuradine | ZINC000000001405 |
| Etofylline-nicotinate | ZINC000005425173 |
| Etolorex | ZINC000003623378 |
| Etoloxamine | ZINC000000001407 |
| Etomidate | ZINC000000001408, ZINC000017364045 |

Table S1. (continued next page)

| Drug name | ZINC ID |
| --- | --- |
| Etomidoline | ZINC000000607859, ZINC000003872532 |
| Etonitazene | ZINC000004215955 |
| Etoperidone | ZINC000003830815 |
| Etoposide-phosphate | ZINC000003920020, ZINC000072186775, ZINC000072186776, ZINC000072186777, ZINC000072186778, ZINC000245205034, ZINC000245205035, ZINC000245205036, ZINC000245205037, ZINC000299754547, ZINC000306146642, ZINC000306146643, ZINC000306146644, ZINC000306146645, ZINC000575418368, ZINC000575418369, ZINC000575418370, ZINC000575418371, ZINC000618072473, ZINC000618072474, ZINC000618072475 |
| Etoposide | ZINC000003830816, ZINC000003830817, ZINC000003830818, ZINC000003830819, ZINC000003925948, ZINC000003925951, ZINC000003938684, ZINC000003977873, ZINC000003977874, ZINC000003978071, ZINC000003978072, ZINC000003978073, ZINC000004245621, ZINC000011615742, ZINC000011615744, ZINC000011615745, ZINC000011615747, ZINC000015449127, ZINC000015449129, ZINC000043648995, ZINC000079216638, ZINC000079228615, ZINC000100023533, ZINC000100023538, ZINC000100042690, ZINC000100042693, ZINC000100042696, ZINC000103553199, ZINC000103553205, ZINC000103553211, ZINC000135672818, ZINC000143020573 |
| Etoprindole | ZINC000005421394 |
| Etoprine | ZINC000000001409 |
| Etoricoxib | ZINC000000579472 |
| Etosalamide | ZINC000003623372 |
| Etoxadrol | ZINC000004626640 |
| Etoxeridine | ZINC000004215962 |
| Etriciguat | ZINC000000603759 |
| Etymemazine | ZINC000000000282, ZINC000001678383 |
| Eucaine | ZINC000000319774 |
| Eucalyptol | ZINC000000967566 |
| Eucatropine | ZINC000007997682, ZINC000011680101 |
| Evacetrapib | ZINC000150338934 |
| Evandamine | ZINC000033754386, ZINC000033754387 |
| Evatanepag | ZINC000001494905 |
| Exaprolol | ZINC000000000285, ZINC000001869701 |
| Exatecan | ZINC000003800855 |
| Exepanol | ZINC000004215979, ZINC000004626743, ZINC000039648339, ZINC000039648340 |
| Exisulind | ZINC000012341529 |
| Ezatiostat | ZINC000056898832 |
| Ezlopitant | ZINC000040664618 |
| Ezogabine | ZINC000000016154 |
| Fabesetron | ZINC000011616570 |
| Fabomotizole | ZINC000023139484 |
| Fadolmidine | ZINC000000006860, ZINC000003822129 |
| Falecalcitriol | ZINC000004474583 |
| Falintolol | ZINC000004213222, ZINC000011524073 |
| Falnidamol | ZINC000001494443 |
| Famciclovir | ZINC000001530635 |
| Famotidine | ZINC000001530636 |
| Famotine | ZINC000000001416 |
| Famprofazone | ZINC000000607861, ZINC000000643070 |
| Fananserin | ZINC000000597400 |
| Fanapanel | ZINC000002004553 |
| Fanetizole | ZINC000000001417 |
| Fantridone | ZINC000000001418 |

Table S1. (continued next page)

| Drug name | ZINC ID |
| --- | --- |
| Farampator | ZINC000000007461 |
| Farglitazar | ZINC000049639808 |
| Fasidotril | ZINC000001545064 |
| Fasiglifam | ZINC000068208039 |
| Fasiplon | ZINC000000004270 |
| Fasitibant | ZINC000095606973 |
| Fasudil | ZINC000000006486 |
| Favipiravir | ZINC000013915654 |
| Faxeladol | ZINC000033966691 |
| Febarbamate | ZINC000004213231, ZINC000005514225, ZINC000005514227, ZINC000005514233 |
| Febuverine | ZINC000022943152, ZINC000022943158, ZINC000022943164 |
| Feclemine | ZINC000004213233, ZINC000033647139 |
| Feclobuzone | ZINC000004216002 |
| Fedotozine | ZINC000003777397, ZINC000004213236 |
| Fedratinib | ZINC000019862646 |
| Fedrilate | ZINC000030691519, ZINC000030691524 |
| Felbamate | ZINC000001530803 |
| Felbinac | ZINC000000002318 |
| Felipyrine | ZINC000000000287, ZINC000002003698 |
| Feloprentan | ZINC000001488192 |
| Femoxetine | ZINC000004216004 |
| Fenalamide | ZINC000002037735, ZINC000002037736 |
| Fenalcomine | ZINC000001842859, ZINC000001842862, ZINC000001842864, ZINC000001842868 |
| Fenamifuril | ZINC000000000288, ZINC000003604409 |
| Fenamole | ZINC000016970473 |
| Fenaperone | ZINC000031545115 |
| Fenbendazole | ZINC000000402911 |
| Fenbufen | ZINC000000001427 |
| Fenbutrazate | ZINC000031623378 |
| Fencamfamin | ZINC000005065159, ZINC000005065160 |
| Fencibutirol | ZINC000000000289, ZINC000001999545 |
| Fenclexonium | ZINC000001481843, ZINC000001842888 |
| Fenclofenac | ZINC000000001428 |
| Fenclonine | ZINC000000000290, ZINC000000156102 |
| Fenclozic_acid | ZINC000000001430 |
| Fendiline | ZINC000001481844, ZINC000002019529 |
| Fendizoate | ZINC000000001431 |
| Fendosal | ZINC000004216021 |
| Fenestrel | ZINC000001556130, ZINC000004626642, ZINC000004900435, ZINC000004900436 |
| Fenethazine | ZINC000000001432 |
| Fenethylline | ZINC000000000293, ZINC000002038451 |
| Fenetradil | ZINC000022463045, ZINC000022860096, ZINC000022860106, ZINC000022860107 |
| Fenflumizole | ZINC000000608375 |
| Fenfluramine | ZINC000000000294, ZINC000000001612 |
| Fenfluthrin | ZINC000001854362 |
| Fengabine | ZINC000004216034 |
| Fenharmane | ZINC000040163092, ZINC000040163093 |
| Fenirofibrate | ZINC000000000297, ZINC000001846331 |
| Fenitrothion | ZINC000000001434 |
| Fenmetozole | ZINC000000001435 |

Table S1. (continued next page)

| Drug name | ZINC ID |
| --- | --- |
| Fenobam | ZINC000000001436 |
| Fenocinol | ZINC000000000299, ZINC000001999304 |
| Fenofibrate | ZINC000000584092 |
| Fenofibric-acid | ZINC000000001984 |
| Fenoprofen | ZINC000000000301, ZINC000000402909 |
| Fenoverine | ZINC000019899628 |
| Fenoxazoline | ZINC000000001437 |
| Fenoxedil | ZINC000004216050 |
| Fenozolone | ZINC000000000302, ZINC000001999283 |
| Fenpentadiol | ZINC000000000303, ZINC000003830834 |
| Fenperate | ZINC000004216054 |
| Fenpipalone | ZINC000000000304, ZINC000002015819 |
| Fenpipramide | ZINC000000001438 |
| Fenpiprane | ZINC000001482064 |
| Fenpiverinium | ZINC000000001439 |
| Fenprinast | ZINC000028221219 |
| Fenquizone | ZINC000000000305, ZINC000001846476 |
| Fenspiride | ZINC000000001441 |
| Fenthion | ZINC000000001443 |
| Fentiazac | ZINC000000001444 |
| Fenticlor | ZINC000000136146 |
| Fenticonazole | ZINC000001542894, ZINC000011525628 |
| Fenyripol | ZINC000000000306, ZINC000002038619 |
| Fepitrizol | ZINC000000001445 |
| Feprazone | ZINC000100016068 |
| Fepromide | ZINC000004213295, ZINC000005639557 |
| Feprosidnine | ZINC000001516802, ZINC000020805626 |
| Fesoterodine | ZINC000001552908 |
| Fevipiprant | ZINC000043101772 |
| Fexicaine | ZINC000004216100 |
| Fexinidazole | ZINC000000001448 |
| Fexofenadine | ZINC000003824921, ZINC000003872566 |
| Fezolamine | ZINC000000001449 |
| Fiboflapon | ZINC000068247071 |
| Fidarestat | ZINC000000002486, ZINC000000005704, ZINC000003789766 |
| Fiduxosin | ZINC000029747110 |
| Filaminast | ZINC000000000308 |
| Filenadol | ZINC000031297962, ZINC000038192488 |
| Filgotinib | ZINC000096174616 |
| Filorexant | ZINC000043201232 |
| Fipamezole | ZINC000003612873, ZINC000005424460 |
| Fipexide | ZINC000019632822 |
| Firategrast | ZINC000030691369 |
| Flamenol | ZINC000000001451 |
| Flavamine | ZINC000000001452 |
| Flavodic_acid | ZINC000004073414 |
| Flavodilol | ZINC000001853520, ZINC000001853521 |
| Flavoxate | ZINC000000608382 |
| Flazalone | ZINC000004213312, ZINC000006002475 |
| Flecainide | ZINC000000896543, ZINC000003830842 |

Table S1. (continued next page)

| Drug name | ZINC ID |
| --- | --- |
| Flerobuterol | ZINC000000000311, ZINC000001999470 |
| Fleroxacin | ZINC000003786299 |
| Flesinoxan | ZINC000053257919 |
| Flestolol | ZINC000002017624, ZINC000002017625 |
| Fletazepam | ZINC000000608384 |
| Flezelastine | ZINC000001534968, ZINC000003785445 |
| Flibanserin | ZINC000052716421 |
| Flindokalner | ZINC000000839816 |
| Floctafenine | ZINC000000607872, ZINC000002014976 |
| Florantyrone | ZINC000002036732 |
| Floredil | ZINC000031597118 |
| Florifenine | ZINC000000537734 |
| Flortaucipir | ZINC000148048492 |
| Flosatidil | ZINC000003780173 |
| Flosequinan | ZINC000000005432, ZINC000004611821 |
| Flosulide | ZINC000000537736 |
| Flotrenizine | ZINC000022463051, ZINC000031597122 |
| Floxacrine | ZINC000000607874, ZINC000001846338 |
| Floxuridine | ZINC000000001457, ZINC000000388678, ZINC000000489135, ZINC000003813010, ZINC000003830846, ZINC000003977743, ZINC000003977744, ZINC000004045293 |
| Fluacizine | ZINC000004213324 |
| Flualamide | ZINC000001843033 |
| Fluanisone | ZINC000004213325 |
| Fluazuron | ZINC000002570819 |
| Flubanilate | ZINC000004216158 |
| Flubendazole | ZINC000003830847 |
| Flubepride | ZINC000000607878, ZINC000003604385 |
| Flucarbril | ZINC000000001459 |
| Flucetorex | ZINC000000607879, ZINC000072266847 |
| Flucindole | ZINC000000000312, ZINC000002007119 |
| Fluconazole | ZINC000000004009 |
| Flucytosine | ZINC000000896546 |
| Fludiazepam | ZINC000000001460 |
| Fludorex | ZINC000000000313, ZINC000002018873 |
| Fludoxopone | ZINC000000537737 |
| Flufenamic-acid | ZINC000000086535 |
| Flufenisal | ZINC000000001461 |
| Flufosal | ZINC000004216175 |
| Flufylline | ZINC000000537738 |
| Fluindarol | ZINC000100229169 |
| Fluindione | ZINC000100091724 |
| Flumatinib | ZINC000068244727 |
| Flumazenil | ZINC000000001464 |
| Flumecinol | ZINC000000000314, ZINC000002002424 |
| Flumequine | ZINC000000000315, ZINC000000156820 |
| Flumeridone | ZINC000000537741 |
| Flumethiazide | ZINC000002040949 |
| Flumetramide | ZINC000000000316, ZINC000002029585 |
| Flumexadol | ZINC000001571539, ZINC000004626645 |
| Flumezapine | ZINC000030690658 |

Table S1. (continued next page)

| Drug name | ZINC ID |
| --- | --- |
| Fluminorex | ZINC000000000318, ZINC000002036974 |
| Flumizole | ZINC000000001465 |
| Flunamine | ZINC000002005763 |
| Flunarizine | ZINC000019360739 |
| Flunidazole | ZINC000000001466 |
| Flunitrazepam | ZINC000003812994 |
| Flunixin | ZINC000000001467 |
| Flunoxaprofen | ZINC000000000319 |
| Fluorescein | ZINC000003872582 |
| Fluorosalan | ZINC000000537744 |
| Fluorouracil | ZINC000038212689 |
| Fluotracen | ZINC000000000320, ZINC000004213359, ZINC000005736145, ZINC000005752379 |
| Fluoxetine | ZINC000001530637, ZINC000001530638 |
| Flupentixol | ZINC000028643021, ZINC000029489118 |
| Fluperamide | ZINC000004216287 |
| Fluperlapine | ZINC000026247473 |
| Fluphenazine | ZINC000019203912 |
| Flupimazine | ZINC000003598320 |
| Flupirtine | ZINC000000001473 |
| Flupranone | ZINC000000537749 |
| Fluprazine | ZINC000072266898 |
| Fluprofen | ZINC000000000322, ZINC000003598315 |
| Fluprofylline | ZINC000000537750 |
| Fluproquazone | ZINC000000001475 |
| Fluquazone | ZINC000000001476 |
| Fluradoline | ZINC000004216304 |
| Fluralaner | ZINC000095598447, ZINC000095598448 |
| Flurantel | ZINC000000537751 |
| Flurazepam | ZINC000000537752 |
| Flurocitabine | ZINC000011612795 |
| Flurofamide | ZINC000008036004 |
| Flusoxolol | ZINC000004216310 |
| Fluspiperone | ZINC000000537753 |
| Fluspirilene | ZINC000000537755 |
| Flutamide | ZINC000003812944 |
| Flutazolam | ZINC000039959893, ZINC000100044499 |
| Flutemazepam | ZINC000002005015, ZINC000002005018 |
| Flutiazin | ZINC000000001478 |
| Flutizenol | ZINC000022463185 |
| Flutomidate | ZINC000000000324, ZINC000072266234 |
| Flutonidine | ZINC000000001480 |
| Flutoprazepam | ZINC000000001481 |
| Flutrimazole | ZINC000003799012, ZINC000003872602 |
| Flutroline | ZINC000049590110, ZINC000049590112 |
| Fluvastatin | ZINC000001530639, ZINC000003830863, ZINC000003830864 |
| Fluvoxamine | ZINC000003872605 |
| Fluzinamide | ZINC000000001482 |
| Fluzoperine | ZINC000031601239 |
| Fodipir | ZINC000058569264 |
| Folescutol | ZINC000033849477 |

Table S1. (continued next page)

| Drug name | ZINC ID |
| --- | --- |
| Folic-acid | ZINC000008577218, ZINC000008585850 |
| Folitixorin | ZINC000004228243, ZINC000004654260 |
| Fominoben | ZINC000000607900 |
| Fomocaine | ZINC000019865692 |
| Fonazine | ZINC000003830865, ZINC000003830866 |
| Fopirtoline | ZINC000031291716 |
| Forasartan | ZINC000005139136 |
| Foretinib | ZINC000043204048 |
| Forodesine | ZINC000013492899 |
| Foropafant | ZINC000001545003 |
| Fosaprepitant | ZINC000003939013 |
| Fosazepam | ZINC000000537760 |
| Fosbretabulin | ZINC000001543513 |
| Fosdagrocorat | ZINC000103298102 |
| Fosenazide | ZINC000000001489 |
| Fosfluconazole | ZINC000003609266 |
| Fosinopril | ZINC000004213382, ZINC000003977764, ZINC000004213382 |
| Fosphenytoin | ZINC000001530922 |
| Fospropofol | ZINC000002519740 |
| Fostamatinib | ZINC000043131420 |
| Fostedil | ZINC000001854337 |
| Fostemsavir | ZINC000014210883 |
| Frabuprofen | ZINC000004213393, ZINC000031555620 |
| Fradafiban | ZINC000003789797 |
| Frentizole | ZINC000008994439 |
| Freselestat | ZINC000000602904, ZINC000003604283 |
| Frovatriptan | ZINC000000018635 |
| Ftaxilide | ZINC000000001490 |
| Ftivazide | ZINC000004602397 |
| Ftormetazine | ZINC000019338440 |
| Ftorpropazine | ZINC000019338446 |
| Fubrogonium | ZINC000001999352, ZINC000001999353 |
| Funapide | ZINC000113473392, ZINC000113473394 |
| Fuprazole | ZINC000022463197 |
| Furafylline | ZINC000009227032 |
| Furalazine | ZINC000004216369 |
| Furaprofen | ZINC000000000331, ZINC000001846457 |
| Furazolidone | ZINC000000113418, ZINC000007997571, ZINC000250090469 |
| Furcloprofen | ZINC000004213406, ZINC000005751728 |
| Furegrelate | ZINC000000001495 |
| Furethidine | ZINC000004213409, ZINC000005650525 |
| Furfenorex | ZINC000031544969, ZINC000031544973 |
| Furmethoxadone | ZINC000000000334, ZINC000001693537 |
| Furobufen | ZINC000004216381 |
| Furodazole | ZINC000018180195 |
| Furofenac | ZINC000004213413, ZINC000005651200 |
| Furomazine | ZINC000000607904, ZINC000003598305 |
| Furonazide | ZINC000000001498, ZINC000012343267, ZINC000257351676 |
| Furosemide | ZINC000000035804 |
| Furostilbestrol | ZINC000000537772 |

Table S1. (continued next page)

| Drug name | ZINC ID |
| --- | --- |
| Fursalan | ZINC000000607905, ZINC000002018678 |
| Furterene | ZINC000000001499 |
| Furtrethonium | ZINC000000001500 |
| Gaboxadol | ZINC000019795995 |
| Gacyclidine | ZINC000004213419 |
| Galantamine | ZINC000000491073 |
| Galdansetron | ZINC000000003706 |
| Galocitabine | ZINC000003967771 |
| Galosemide | ZINC000000537777 |
| Galunisertib | ZINC000003959536 |
| Gamfexine | ZINC000001481846, ZINC000002015666 |
| Ganciclovir | ZINC000000001505 |
| Gandotinib | ZINC000068245097 |
| Ganetespib | ZINC000043130413 |
| Ganglefene | ZINC000002040876, ZINC000002040877, ZINC000002040878, ZINC000002040879 |
| Gantofiban | ZINC000022447381 |
| Gapromidine | ZINC000013816068 |
| Garenoxacin | ZINC000003585048 |
| Gatifloxacin | ZINC000003607120, ZINC000038197764 |
| Gdc-0994 | ZINC000144904353, ZINC000144904566 |
| Gedatolisib | ZINC000049757175 |
| Gedocarnil | ZINC000000537780 |
| Gefitinib | ZINC000019632614 |
| Gemazocine | ZINC000001481848 |
| Gemcitabine | ZINC000018279854 |
| Gemfibrozil | ZINC000001530641 |
| Gemifloxacin | ZINC000022059926, ZINC000022059930 |
| Gentamicin | ZINC000008143541, ZINC000008214392 |
| Gepirone | ZINC000002021499 |
| Gepotidacin | ZINC000138562667, ZINC000223227887 |
| Gevotroline | ZINC000029486404 |
| Gilteritinib | ZINC000113476229 |
| Gimatecan | ZINC000003993519 |
| Giripladib | ZINC000085537113 |
| Girisopam | ZINC000000000340 |
| Gisadenafil | ZINC000034016204 |
| Glafenine | ZINC000000607910, ZINC000003872687 |
| Glemanserin | ZINC000001481850, ZINC000001843091 |
| Glibornuride | ZINC000002012812 |
| Glibutimine | ZINC000031601255, ZINC000031601260 |
| Glicaramide | ZINC000004216401 |
| Glicetanile | ZINC000000607915 |
| Gliclazide | ZINC000000523925, ZINC000000523926, ZINC000012461841 |
| Glicondamide | ZINC000000537790 |
| Glidazamide | ZINC000000001509 |
| Gliflumide | ZINC000001481851 |
| Glipalamide | ZINC000005781566, ZINC000005781568 |
| Glipizide | ZINC000000537795 |
| Gliquidone | ZINC000001482077 |
| Glisamuride | ZINC000000537797 |

Table S1. (continued next page)

| Drug name | ZINC ID |
| --- | --- |
| Glisentide | ZINC000000537793 |
| Glisindamide | ZINC000000537799 |
| Glisolamide | ZINC000000537802 |
| Glisoxepide | ZINC000000537804 |
| Glucametacin | ZINC000077293064, ZINC000080060444, ZINC000080060453, ZINC000097974783, ZINC000097974785, ZINC000110343950, ZINC000110343953, ZINC000575346762 |
| Glucosamine-sulfate | ZINC000001532837, ZINC000002558712, ZINC000003869696, ZINC000003869698, ZINC000003869699, ZINC000004095529, ZINC000005830072, ZINC000043763830, ZINC000043763831, ZINC000141333481, ZINC000221554217, ZINC000221554276, ZINC000616583397 |
| Glunicate | ZINC000004216419 |
| Glyburide | ZINC000000537805 |
| Glybuzole | ZINC000000001512 |
| Glycerol-phenylbutyrate | ZINC000038945666 |
| Glyclopyramide | ZINC000000001513 |
| Glyconiazide | ZINC000004539118, ZINC000004539124, ZINC000017286321, ZINC000101921914, ZINC000114096862 |
| Glycopyrrolate | ZINC000000000346, ZINC000000896958, ZINC000000896968, ZINC000000968301 |
| Glycyclamide | ZINC000000001514 |
| Glyhexamide | ZINC000000001515 |
| Glymidine | ZINC000002040778 |
| Glyoctamide | ZINC000000001516 |
| Glyparamide | ZINC000000537807 |
| Glypinamide | ZINC000000001517 |
| Glysobuzole | ZINC000000001519 |
| Golotimod | ZINC000001549362, ZINC000002522604, ZINC000004544882, ZINC000004544883 |
| Golvatinib | ZINC000043195317 |
| Gosogliptin | ZINC000023247686 |
| Granisetron | ZINC000000000347, ZINC000100018852 |
| Grapiprant | ZINC000038228051 |
| Grazoprevir | ZINC000095551509 |
| Grepafloxacin | ZINC000000587541, ZINC000000967787 |
| Gs-6201 | ZINC000029055563 |
| Gs-9667 | ZINC000115619865, ZINC000256680330, ZINC000256680332, ZINC000256680333 |
| Gsk-1070916 | ZINC000043170519 |
| Gsk163090 | ZINC000034451922 |
| Gsk189254 | ZINC000003961799 |
| Gsk239512 | ZINC000003961802 |
| Gsk-2636771 | ZINC000077024226 |
| Gsk-461364 | ZINC000043154472, ZINC000059715991 |
| Guabenxan | ZINC000003647976 |
| Guacetisal | ZINC000000000348 |
| Guadecitabine | ZINC000043203165, ZINC000100285210, ZINC000100285212, ZINC000257351737, ZINC000257351738, ZINC000575627124 |
| Guafecainol | ZINC000002019914, ZINC000002019915 |
| Guaiacol_carbonate | ZINC000000000350 |
| Guaiacolsulfonic-acid | ZINC000002510314 |
| Guaiactamine | ZINC000000050098 |
| Guaiazulene-sulfonic-acid | ZINC000002034069 |
| Guaimesal | ZINC000000003877, ZINC000003780898 |

Table S1. (continued next page)

| Drug name | ZINC ID |
| --- | --- |
| Guaisteine | ZINC000002016028, ZINC000002016029 |
| Guanabenz | ZINC000000001522 |
| Guanacline | ZINC000000001523 |
| Guanadrel | ZINC000001530647, ZINC000004097338 |
| Guanclofine | ZINC000001846297 |
| Guancydine | ZINC000015848248 |
| Guanethidine | ZINC000001530648 |
| Guanfacine | ZINC000003872738 |
| Guanisoquin | ZINC000004216440 |
| Guanoxabenz | ZINC000005784328 |
| Guanoxan | ZINC000000000357, ZINC000004213470 |
| Gw590735 | ZINC000016052349 |
| Gw842166x | ZINC000003947932 |
| Halazepam | ZINC000000537811 |
| Halazone | ZINC000001482080 |
| Halethazole | ZINC000000537813 |
| Halofantrine | ZINC000001530866, ZINC000001542393 |
| Halonamine | ZINC000003609274, ZINC000003609275 |
| Halopemide | ZINC000000537818 |
| Halopenium | ZINC000003609272 |
| Haloperidol | ZINC000000537822 |
| Haloxazolam | ZINC000040017477, ZINC000040017478 |
| Heliomycin | ZINC000100150837 |
| Heptolamide | ZINC000000001529 |
| Hepzidine | ZINC000001482083 |
| Hesperetin | ZINC000000039091, ZINC000000039092 |
| Hesperidin | ZINC000008143568, ZINC000008214774, ZINC000008382286, ZINC000008382287, ZINC000008680006, ZINC000008680007, ZINC000038144568, ZINC000038144569, ZINC000038144570, ZINC000038144571, ZINC000049889104, ZINC000067902505, ZINC000085340597, ZINC000095568264, ZINC000097972618, ZINC000100204049, ZINC000100204053, ZINC000100204058, ZINC000100204061, ZINC000103938984, ZINC000103938990, ZINC000103938998, ZINC000103939005, ZINC000104889096, ZINC000238924078, ZINC000238924079, ZINC000238924080, ZINC000247658398, ZINC000248139797, ZINC000248139800, ZINC000252008992, ZINC000252008995, ZINC000252008997, ZINC000252009000, ZINC000253499896, ZINC000253499897, ZINC000253499898, ZINC000253499899 |
| Heteronium | ZINC000000000359, ZINC000001999536, ZINC000001999537, ZINC000001999538 |
| Hexacyprone | ZINC000000000360, ZINC000001846045 |
| Hexadiline | ZINC000003598256, ZINC000005413296 |
| Hexaprofen | ZINC000001999265, ZINC000028711044 |
| Hexasonium | ZINC000004213493, ZINC000005377707 |
| Hexedine | ZINC000019319125 |
| Hexetidine | ZINC000019318917, ZINC000019321450 |
| Hexocyclium | ZINC000000000363, ZINC000000897263 |
| Hexopyrronium | ZINC000000000364, ZINC000004213498, ZINC000031623233, ZINC000031623234 |
| Hexylcaine | ZINC000000000365, ZINC000002034892 |
| Histapyrrodine | ZINC000000001534 |
| Homatropine | ZINC000100016393, ZINC000100016396 |
| Homidium | ZINC000000119632 |
| Homochlorcyclizine | ZINC000019365270 |
| Homofenazine | ZINC000019366089 |

Table S1. (continued next page)

| Drug name | ZINC ID |
| --- | --- |
| Homopipramol | ZINC000022463208 |
| Homosalate | ZINC000000000370, ZINC000000393812, ZINC000000393813, ZINC000000393814 |
| Hopantenic-acid | ZINC000002017228, ZINC000002018156 |
| Hoquizil | ZINC000000537831 |
| Hycanthone | ZINC000003830916 |
| Hydracarbazine | ZINC000005842953 |
| Hydralazine | ZINC000012360535 |
| Hydrobentizide | ZINC000001842993, ZINC000001842995 |
| Hydrochlorothiazide | ZINC000000896569 |
| Hydroflumethiazide | ZINC000000897225 |
| Hydromorphinol | ZINC000004216508 |
| Hydromorphone_sulfate | ZINC000044963409, ZINC000097974884 |
| Hydromorphone | ZINC000000402954 |
| Hydroquinine | ZINC000000000219, ZINC000000000220, ZINC000000056607, ZINC000000156023, ZINC000000389626, ZINC000001317949, ZINC000003872852, ZINC000003977898, ZINC000003977899, ZINC000004000060, ZINC000005317082, ZINC000007998025 |
| Hydroxindasol | ZINC000000001542 |
| Hydroxychloroquine | ZINC000001530652, ZINC000001530654 |
| Hydroxyestrone_diacetate | ZINC000002557209, ZINC000005166858, ZINC000005228505, ZINC000005228508, ZINC000005228512, ZINC000005228515, ZINC000079199213, ZINC000139021488, ZINC000253492745, ZINC000254855482 |
| Hydroxyethylpromethazine | ZINC000002038911, ZINC000002038912 |
| Hydroxyhexamide | ZINC000000000374, ZINC000002032663 |
| Hydroxypethidine | ZINC000000001544 |
| Hydroxystenozole | ZINC000004430803 |
| Hydroxystilbamidine | ZINC000000001547 |
| Hydroxyzine | ZINC000019364222, ZINC000019364224 |
| Hyoscyamine | ZINC000100009280 |
| Ibafloxacin | ZINC000001999530, ZINC000004626650 |
| Ibipinabant | ZINC000003964747, ZINC000013559250 |
| Ibodutant | ZINC000028711707 |
| Ibudilast | ZINC000000004234 |
| Ibufenac | ZINC000000001549 |
| Ibuprofen_guaiacol_ester | ZINC000000363994, ZINC000000363995 |
| Ibuprofen-piconol | ZINC000000000380, ZINC000001851415 |
| Ibuproxam | ZINC000000000381, ZINC000005284667 |
| Ibutamoren | ZINC000001543181 |
| Ibuterol | ZINC000001846363, ZINC000003643738 |
| Ibuverine | ZINC000000000382, ZINC000005759351 |
| Icaridin | ZINC000005922357, ZINC000005922359, ZINC000005925029, ZINC000005925031 |
| Iclaprim | ZINC000001486728, ZINC000003612862 |
| Iclazepam | ZINC000001846268 |
| Icofungipen | ZINC000003801186 |
| Icopezil-maleate | ZINC000000537848 |
| Icotidine | ZINC000008144430 |
| Icotinib | ZINC000043207566 |
| Idalopirdine | ZINC000095936819 |
| Idanpramine | ZINC000005733284 |
| Idaverine | ZINC000004213537 |
| Idazoxan | ZINC000000013748, ZINC000000900630 |

Table S1. (continued next page)

| Drug name | ZINC ID |
| --- | --- |
| Idelalisib | ZINC000013986658 |
| Idenast | ZINC000033754357 |
| Idralfidine | ZINC000003604290, ZINC000038811444, ZINC000038811445, ZINC000038811446 |
| Idramantone | ZINC000012362955 |
| Idrapril | ZINC000000004294 |
| Idronoxil | ZINC000001491943 |
| Idropranolol | ZINC000000000383, ZINC000005760477 |
| Ifenprodil | ZINC000000014551, ZINC000000014669, ZINC000002559943, ZINC000003616630 |
| Ifetroban | ZINC000003793091 |
| Igmesine | ZINC000001481860, ZINC000005923781 |
| Ilaprazole | ZINC000003813639, ZINC000009084028 |
| Iliparcil | ZINC000003790919 |
| Ilomastat | ZINC000003780014, ZINC000003872718, ZINC000003872719, ZINC000003872720 |
| Iloperidone | ZINC000001548097 |
| Ilorasertib | ZINC000063298074 |
| Imafen | ZINC000000000384, ZINC000004213542 |
| Imagabalin | ZINC000038276539 |
| Imanixil | ZINC000000537853 |
| Imatinib | ZINC000019632618 |
| Imazodan | ZINC000000001553 |
| Imiclopazine | ZINC000022463214 |
| Imidafenacin | ZINC000000007368 |
| Imidaprilat | ZINC000004216543 |
| Imidapril | ZINC000003784427 |
| Imidocarb | ZINC000000073661 |
| Imidoline | ZINC000000001556 |
| Imidurea | ZINC000004213546, ZINC000004245708, ZINC000004245710 |
| Imiglitazar | ZINC000049756486 |
| Imiloxan | ZINC000000000385, ZINC000003995466 |
| Imipramine | ZINC000000020245 |
| Imipraminoxide | ZINC000000001557 |
| Imiquimod | ZINC000019632912 |
| Imirestat | ZINC000000003724 |
| Imolamine | ZINC000000001560 |
| Imoxiterol | ZINC000002019981, ZINC000002019982, ZINC000002019983, ZINC000003785475 |
| Impromidine | ZINC000004216553 |
| Imuracetam | ZINC000004216556 |
| Inaperisone | ZINC000000000387, ZINC000002016043 |
| Incb-9471 | ZINC000043171152 |
| Indacaterol | ZINC000035801098 |
| Indacrinone | ZINC000000538561, ZINC000000538575 |
| Indalpine | ZINC000004674528 |
| Indanazoline | ZINC000000001563 |
| Indanidine | ZINC000000001564 |
| Indapamide | ZINC000000601305, ZINC000000643114 |
| Indatraline | ZINC000000001565, ZINC000003872904, ZINC000003872906, ZINC000003872907 |
| Indecainide | ZINC000001855421 |
| Indeglitazar | ZINC000039202807 |
| Indeloxazine | ZINC000000009710, ZINC000000900543 |
| Indenolol | ZINC000000000389, ZINC000001842844 |

Table S1. (continued next page)

| Drug name | ZINC ID |
| --- | --- |
| Indibulin | ZINC000037866151 |
| Indinavir | ZINC000022448696 |
| Indiplon | ZINC000000538650 |
| Indisetron | ZINC000100102461 |
| Indisulam | ZINC000000600748 |
| Indobufen | ZINC000000003955, ZINC000003187624 |
| Indocate | ZINC000000537860 |
| Indolidan | ZINC000013544987 |
| Indomethacin | ZINC000000601283 |
| Indopanolol | ZINC000004213558, ZINC000039375686 |
| Indopine | ZINC000001482088 |
| Indoramin | ZINC000000001567 |
| Indorenate | ZINC000000000392, ZINC000001842823 |
| Indoxole | ZINC000000001568 |
| Indriline | ZINC000001678256, ZINC000005062865 |
| Infigratinib | ZINC000072105034 |
| Inicarone | ZINC000000001569 |
| Inogatran | ZINC000003794149 |
| Inosine | ZINC000000895160, ZINC000002573068, ZINC000004015531, ZINC000005127789, ZINC000008613160, ZINC000008613161, ZINC000008614390, ZINC000008652273, ZINC000008652274, ZINC000008855117, ZINC000012358753, ZINC000016951611, ZINC000018123143 |
| Int131 | ZINC000006485945 |
| Intoplicine | ZINC000001629400 |
| Intrazole | ZINC000000001573 |
| Ipamorelin | ZINC000029562299 |
| Ipatasertib | ZINC000068250459 |
| Ipazilide | ZINC000001536782 |
| Ipenoxazone | ZINC000003779985 |
| ipragliflozin | ZINC000038897728, ZINC000138361877, ZINC000165806177, ZINC000165806297, ZINC000205605627, ZINC000306122897, ZINC000306122898, ZINC000306122899, ZINC000605694508, ZINC000669678948 |
| Ipramidil | ZINC000000000394 |
| Ipratropium | ZINC000100015775, ZINC000100015780 |
| Ipravacaine | ZINC000003806302, ZINC000013905232 |
| Ipriflavone | ZINC000000004016 |
| Iprindole | ZINC000000001576 |
| Iprocrolol | ZINC000000000397, ZINC000033650026 |
| Iprofenin | ZINC000000001578 |
| Ipronidazole | ZINC000000001580 |
| Iproxamine | ZINC000000001581 |
| Iprozilamine | ZINC000000001582 |
| Ipsalazide | ZINC000004216628 |
| Ipsapirone | ZINC000001999529 |
| Iquindamine | ZINC000000001583 |
| Irampanel | ZINC000000007363 |
| Irbesartan | ZINC000003872931 |
| Irindalone | ZINC000022463226 |
| Irinotecan | ZINC000001612996 |
| Irloxacin | ZINC000000001584 |
| Irolapride | ZINC000000000398, ZINC000004213597 |

Table S1. (continued next page)

| Drug name | ZINC ID |
| --- | --- |
| Irosustat | ZINC000001549366 |
| Iroxanadine | ZINC000001481826, ZINC000013916716 |
| Irsogladine | ZINC000000002645 |
| Irtemazole | ZINC000005424882, ZINC000013884467 |
| Isaglidole | ZINC000000010612 |
| Isalmadol | ZINC000000588684 |
| Isalsteine | ZINC000003780182, ZINC000004632733, ZINC000013889553, ZINC000013889554 |
| Isamfazone | ZINC000000607932, ZINC000001846300 |
| Isamoltan | ZINC000000002322, ZINC000003995330 |
| Isamoxole | ZINC000004216638 |
| Isatoribine | ZINC000008214713 |
| Isbogrel | ZINC000002019975, ZINC000013437583 |
| Isbufylline | ZINC000000004317 |
| Isobromindione | ZINC000100369297, ZINC000100369302 |
| Isobucaine | ZINC000002019366 |
| Isoconazole | ZINC000000607934, ZINC000003872945 |
| Isocromil | ZINC000000001588 |
| Isoetharine | ZINC000000000401, ZINC000000402979, ZINC000000402980, ZINC000000402981 |
| Isofezolac | ZINC000000537869 |
| Isomazole | ZINC000005319251, ZINC000005319254 |
| Isometamidium | ZINC000004216649 |
| Isometheptene | ZINC000001683250, ZINC000002036972 |
| Isomolpan | ZINC000002016012 |
| Isomylamine | ZINC000001719281 |
| Isoniazid | ZINC000000001590 |
| Isonixin | ZINC000000001591 |
| Isoprazone | ZINC000000001593 |
| Isoprofen | ZINC000000000403, ZINC000001846286, ZINC000001846287, ZINC000001846288 |
| Isopromethazine | ZINC000005141517, ZINC000005141705 |
| Isopropamide | ZINC000001530668 |
| Isosorbide-dinitrate | ZINC000003872966, ZINC000005157275, ZINC000009212416, ZINC000017920779, ZINC000018084626, ZINC000018089317, ZINC000018089664, ZINC000086860211 |
| Isospaglumic-acid | ZINC000001532510, ZINC000001532511, ZINC000001532512, ZINC000001532513 |
| Isosulpride | ZINC000004213610, ZINC000005518903 |
| Isothipendyl | ZINC000000000404, ZINC000001678303 |
| Isoxaprolol | ZINC000000000406, ZINC000003647660 |
| Isoxepac | ZINC000000001595 |
| Isoxsuprine | ZINC000000000407, ZINC000001319967, ZINC000009302317, ZINC000011616526 |
| Ispronicline | ZINC000003961864 |
| Israpafant | ZINC000001533241, ZINC000003776441 |
| Istaroxime | ZINC000004392967 |
| Istradefylline | ZINC000003803921 |
| Itameline | ZINC000001889902 |
| Itasetron | ZINC000100090217 |
| Itazigrel | ZINC000000607940 |
| Iti-007 | ZINC000116262036, ZINC000137099035, ZINC000141600395, ZINC000143595671 |
| Itopride | ZINC000000537874 |
| Itraconazole | ZINC000003830973, ZINC000003830974, ZINC000003830975, ZINC000003830976, ZINC000004097343, ZINC000004097344, ZINC000028007115, ZINC000028007119 |

Table S1. (continued next page)

| Drug name | ZINC ID |
| --- | --- |
| Itriglumide | ZINC000001544617 |
| Itrocainide | ZINC000004216669 |
| Ivarimod | ZINC000004216671 |
| Ivoqualine | ZINC000001841488 |
| Izonsteride | ZINC000001544066 |
| Ji-101 | ZINC000038255464 |
| Jnj-38877605 | ZINC000043170515 |
| Jnj-42756493 | ZINC000168520308 |
| Kainic-acid | ZINC000001481864, ZINC000001555758, ZINC000002008786, ZINC000003872987, ZINC000003872989, ZINC000003995575, ZINC000005195168, ZINC000005382902 |
| Ketanserin | ZINC000000537877 |
| Ketazocine | ZINC000001999400 |
| Ketipramine | ZINC000000001599 |
| Ketobemidone | ZINC000000001600 |
| Ketocaine | ZINC000000000412 |
| Ketocainol | ZINC000000000413, ZINC000002029398 |
| Ketoconazole | ZINC000000643138, ZINC000000643143, ZINC000000643153, ZINC000003872994 |
| Ketorfanol | ZINC000004213629 |
| Ketorolac | ZINC000000002279, ZINC000000011012 |
| Ketotifen | ZINC000000004351 |
| Ketotrexate | ZINC000008628600, ZINC000008628601 |
| Khellin | ZINC000000056654 |
| Kinetin | ZINC000000001601 |
| Kojic_acid | ZINC000013831818 |
| Krn-633 | ZINC000003966300 |
| Kw-2449 | ZINC000043201999, ZINC000095911083 |
| Kx2-391 | ZINC000043152787 |
| Labetalol | ZINC000000000416, ZINC000000403010, ZINC000000403011 |
| Lachesine | ZINC000002002895 |
| Lacosamide | ZINC000000007673, ZINC000029562604 |
| Ladarixin | ZINC000014211055, ZINC000084596756 |
| Lafutidine | ZINC000003781682, ZINC000004654795 |
| Lamifiban | ZINC000003782835 |
| Lamivudine | ZINC000000012346, ZINC000001616230, ZINC000016952920, ZINC000017187710 |
| Lamotrigine | ZINC000000013156 |
| Lamtidine | ZINC000001532376 |
| Lanperisone | ZINC000000002655 |
| Lapatinib | ZINC000001550477 |
| Laprafylline | ZINC000022463231 |
| Laquinimod | ZINC000100004621 |
| Laropiprant | ZINC000003961849 |
| Las101057 | ZINC000035000463 |
| Lasmiditan | ZINC000003818355 |
| Lasofoxifene | ZINC000003918428 |
| Latrepirdine | ZINC000008144259 |
| Lavoltidine | ZINC000001532377 |
| Lazabemide | ZINC000001542229 |
| Ledazerol | ZINC000004674529 |
| Ledipasvir | ZINC000150338819 |

Table S1. (continued next page)

| Drug name | ZINC ID |
| --- | --- |
| Ledoxantrone | ZINC000029334414 |
| Leflunomide | ZINC000000004840 |
| Lefradafiban | ZINC000027093989 |
| Leiopyrrole | ZINC000000001603 |
| Lemborexant | ZINC000118073503, ZINC000209650589, ZINC000209650622, ZINC000209650650 |
| Lemidosul | ZINC000033754369 |
| Leminoprazole | ZINC000000005076, ZINC000004626804 |
| Lenalidomide | ZINC000001997127, ZINC000003604264 |
| Lenperone | ZINC000000537887 |
| Lenvatinib | ZINC000003816292 |
| Leq506 | ZINC000103298500 |
| Lerisetron | ZINC000000007943 |
| Lesinurad | ZINC000084757007 |
| Lesogaberan | ZINC000040829484, ZINC000040934568 |
| Lesopitron | ZINC000001547604 |
| Lestaurtinib | ZINC000003781738 |
| Letaxaban | ZINC000013986542 |
| Leteprinim | ZINC000005117168 |
| Letermovir | ZINC000100369359 |
| Letosteine | ZINC000001846340, ZINC000001846342, ZINC000001846343, ZINC000001846344 |
| Leucocianidol | ZINC000000000419, ZINC000000968223, ZINC000003591028, ZINC000004096940, ZINC000004213653, ZINC000013508443 |
| Levallorphan | ZINC000003875992 |
| Levobetaxolol | ZINC000001530567, ZINC000001530568 |
| Levodropropizine | ZINC000019594545, ZINC000019594547 |
| Levofacetoperane | ZINC000002019952, ZINC000004626654, ZINC000032228596, ZINC000032228598 |
| Levofuraltadone | ZINC000031261645, ZINC000031261650 |
| Levomefolic-acid | ZINC000002005305, ZINC000002572666, ZINC000004228266, ZINC000004228267 |
| Levomenol | ZINC000001609418, ZINC000001849758 |
| Levomepromazine | ZINC000000020246 |
| Levometiomeprazine | ZINC000000000483, ZINC000000001615 |
| Levomoramide | ZINC000029402221 |
| Levophenacylmorphan | ZINC000004216728 |
| Levopropoxyphene | ZINC000001530767 |
| Levoprotiline | ZINC000001481890 |
| Levosemotiadil | ZINC000001552136 |
| Levosulpiride | ZINC000000057008 |
| Lexacalcitol | ZINC000004474609 |
| Lexibulin | ZINC000043024000 |
| Lexipafant | ZINC000001851122 |
| Liafensine | ZINC000095598450 |
| Liarozole | ZINC000005425356, ZINC000011687637 |
| Libenzapril | ZINC000004216734 |
| Licofelone | ZINC000003805769 |
| Licostinel | ZINC000001483505 |
| Lidamidine | ZINC000013115357 |
| Lidanserin | ZINC000003775733, ZINC000013883416 |
| Lidocaine | ZINC000000020237 |
| Lidofenin | ZINC000000001618 |
| Lidoflazine | ZINC000022034381 |

Table S1. (continued next page)

| Drug name | ZINC ID |
| --- | --- |
| Lidorestat | ZINC000000538652 |
| Lifarizine | ZINC000019368434 |
| Lifibrol | ZINC000000000424, ZINC000001997397 |
| Lifitegrast | ZINC000084668739 |
| Limazocic | ZINC000003803665 |
| Linaprazan | ZINC000001547156 |
| Linarotene | ZINC000003927341 |
| Linezolid | ZINC000000001622, ZINC000002008866 |
| Linifanib | ZINC000006718813 |
| Linogliride | ZINC000100371465 |
| Linopirdine | ZINC000000537908 |
| Linotroban | ZINC000000004390 |
| Linsitinib | ZINC000100071817 |
| Lintitript | ZINC000000537914 |
| Lintopride | ZINC000000004725 |
| Lipoic-acid-alpha | ZINC000001532729, ZINC000001544807 |
| Lirequinil | ZINC000003784128 |
| Lirimilast | ZINC000001547848 |
| Liroldine | ZINC000000537922 |
| Lisdexamfetamine | ZINC000011680943 |
| Lisinopril | ZINC000003812863 |
| Lisofylline | ZINC000001887263 |
| Lisuride | ZINC000003831001 |
| Litomeglovir | ZINC000084759446 |
| Litoxetine | ZINC000000003647 |
| Litracen | ZINC000004216753 |
| Litronesib | ZINC000056898863 |
| Lividomycin | ZINC000253673980, ZINC000257402766, ZINC000257402767, ZINC000257402768, ZINC000257402769 |
| Lixazinone | ZINC000005318265 |
| Lixivaptan | ZINC000000600399 |
| Lobeglitazone | ZINC000033972992, ZINC000033972993 |
| Lobeline | ZINC000000001624, ZINC000000156831 |
| Lobendazole | ZINC000000049910 |
| Lobenzarit | ZINC000000001625 |
| Lobucavir | ZINC000003781393 |
| Lobuprofen | ZINC000004213673, ZINC000005138307 |
| Lodaxaprine | ZINC000002016056 |
| Lodazecar | ZINC000004216756 |
| Lodenosine | ZINC000003783669 |
| Lodinixil | ZINC000000001626 |
| Lodiperone | ZINC000053149862 |
| Lofemizole | ZINC000000001627 |
| Lofendazam | ZINC000000001628 |
| Lofentanil | ZINC000004216763 |
| Lofepramine | ZINC000001542929 |
| Lombazole | ZINC000004213681, ZINC000005425510 |
| Lomefloxacin | ZINC000003873156, ZINC000003873157 |
| Lomeguatrib | ZINC000013470184 |
| Lomerizine | ZINC000019362693 |

Table S1. (continued next page)

| Drug name | ZINC ID |
| --- | --- |
| Lometraline | ZINC000000000426, ZINC000002009618 |
| Lometrexol | ZINC000008577213 |
| Lomevactone | ZINC000005706462, ZINC000006441776 |
| Lomitapide | ZINC000027990463 |
| Lonafarnib | ZINC000003950115 |
| Lonapalene | ZINC000000001630 |
| Lonaprofen | ZINC000000000428, ZINC000001846409 |
| Lonazolac | ZINC000000001631 |
| Lonidamine | ZINC000000001632 |
| Loperamide-oxide | ZINC000000537930, ZINC000261506627 |
| Loperamide | ZINC000000537928 |
| Lopinavir | ZINC000003951740, ZINC000004172317, ZINC000011616285, ZINC000011616286, ZINC000011616287, ZINC000011616288, ZINC000028525391, ZINC000043773445, ZINC000043773446, ZINC000043773447, ZINC000097975231, ZINC000100028551, ZINC000101007591, ZINC000110906785 |
| Lopirazepam | ZINC000000000429, ZINC000001846399 |
| Lorapride | ZINC000000000430, ZINC000001846131 |
| Loratadine | ZINC000000537931 |
| Lorazepam | ZINC000000000431, ZINC000000896595 |
| Lorbamate | ZINC000001722646, ZINC000002014729 |
| Lorcainide | ZINC000000537935 |
| Lorcinadol | ZINC000000001634 |
| Loreclezole | ZINC000003785289 |
| Lorglumide | ZINC000003782183, ZINC000003789437 |
| Lormetazepam | ZINC000000000432, ZINC000002040287 |
| Lorpiprazole | ZINC000033827894 |
| Lortalamine | ZINC000000001635 |
| Lorzafone | ZINC000004216774 |
| Losartan | ZINC000003873160 |
| Losindole | ZINC000004216775 |
| Losmapimod | ZINC000035793138 |
| Losmiprofen | ZINC000000000433, ZINC000001846060 |
| Losoxantrone | ZINC000001583637 |
| Losulazine | ZINC000004216779 |
| Lotrafiban | ZINC000003812598 |
| Lotrifen | ZINC000000001637 |
| Lotucaine | ZINC000000000434, ZINC000003608747 |
| Lovastatin | ZINC000003812841, ZINC000003831008, ZINC000003831009, ZINC000003831010, ZINC000003831011, ZINC000004245664, ZINC000008681777, ZINC000008681778, ZINC000008681779, ZINC000014984346, ZINC000027646885, ZINC000027899841, ZINC000027899846, ZINC000039341568, ZINC000043772719, ZINC000043772720, ZINC000043772721, ZINC000043772723, ZINC000043772724, ZINC000043772726 |
| Loviride | ZINC000000598073, ZINC000002020240 |
| Loxapine | ZINC000019796158 |
| Loxoprofen | ZINC000000000435, ZINC000001846128, ZINC000001846129, ZINC000001846130 |
| Loxoribine | ZINC000004674533 |
| Lubazodone | ZINC000003809198 |
| Lubeluzole | ZINC000000537944 |
| Lucanthone | ZINC000003831012 |
| Lucitanib | ZINC000077024213 |
| Lufironil | ZINC000001541061 |

Table S1. (continued next page)

| Drug name | ZINC ID |
| --- | --- |
| Lufuradom | ZINC000000607947, ZINC000004213704 |
| Lumacaftor | ZINC000064033452 |
| Lumefantrine | ZINC000013831150, ZINC000013831151 |
| Lumiracoxib | ZINC000000007563 |
| Lupitidine | ZINC000005634902 |
| Luprostiol | ZINC000004474615 |
| Lurasidone | ZINC000003927822 |
| Lurosetron-mesylate | ZINC000000011481 |
| Lurtotecan | ZINC000022010625 |
| Lusaperidone | ZINC000001903948 |
| luseogliflozin | ZINC000049087932 |
| Luxabendazole | ZINC000001842766 |
| Lxr-623 | ZINC000040379749 |
| Ly-2584702 | ZINC000043204100 |
| Ly-2874455 | ZINC000073069242, ZINC000097758759, ZINC000113514933, ZINC000113514935 |
| Ly-518674 | ZINC000000595076 |
| Lysergide | ZINC000001320003, ZINC000039569537, ZINC000071754993, ZINC000096903803 |
| Mabuprofen | ZINC000001999468, ZINC000001999469 |
| Mabuterol | ZINC000000000438, ZINC000001846294 |
| Macitentan | ZINC000043202140 |
| Mafoprazine | ZINC000004213714 |
| Malathion | ZINC000001530799, ZINC000001530800 |
| Mantabegron | ZINC000031572981, ZINC000031572985 |
| Mapinastine | ZINC000022441789 |
| Mapracorat | ZINC000035952535 |
| Maprotiline | ZINC000001530688 |
| Maralixibat | ZINC000003923919 |
| Maraviroc | ZINC000100003902 |
| Marbofloxacin | ZINC000000537947 |
| Maribavir | ZINC000003824412 |
| Marimastat | ZINC000001544157 |
| Mariptiline | ZINC000002032191, ZINC000002032192, ZINC000002032195 |
| Maropitant | ZINC000040664622 |
| Maroxepin | ZINC000000001647 |
| Masitinib | ZINC000034177219 |
| Mavacoxib | ZINC000003814698 |
| Mavatrep | ZINC000043175494 |
| Maxacalcitol | ZINC000004474617 |
| Mazapertine | ZINC000052956332 |
| Mazindol | ZINC000000000440, ZINC000000896621 |
| Mazokalim | ZINC000003916953 |
| Mebendazole | ZINC000000121541 |
| Mebenoside | ZINC000004213726, ZINC000033827949 |
| Mebeverine | ZINC000003813087, ZINC000003831024 |
| Mebezonium | ZINC000000001650 |
| Mebhydrolin | ZINC000000001651 |
| Mebrofenin | ZINC000000537957 |
| Mebutamate | ZINC000001640015, ZINC000002041322 |
| Mebutizide | ZINC000000607950, ZINC000005651103, ZINC000005651424, ZINC000005651432 |
| Mecarbinate | ZINC000000001652 |

Table S1. (continued next page)

| Drug name | ZINC ID |
| --- | --- |
| Meciadanol | ZINC000001845185 |
| Meclizine | ZINC000019361042, ZINC000019594557 |
| Meclofenamic-acid | ZINC000000001655 |
| Meclofenoxate | ZINC000001531029 |
| Meclonazepam | ZINC000004216812 |
| Mecloqualone | ZINC000000001656 |
| Mecloxamine | ZINC000000000443, ZINC000033827951, ZINC000033827953, ZINC000033827955 |
| Medazepam | ZINC000000001659 |
| Medibazine | ZINC000022463243 |
| Medorinone | ZINC000005765028 |
| Medroxalol | ZINC000001570643, ZINC000001570644, ZINC000001570645, ZINC000004213738 |
| Medrylamine | ZINC000000000444, ZINC000005764844 |
| Mefenamic-acid | ZINC000000020241 |
| Mefenidramium | ZINC000000001666 |
| Mefeserpine | ZINC000004213752 |
| Mefexamide | ZINC000002024936 |
| Mefloquine | ZINC000000537964, ZINC000000897085, ZINC000000897089, ZINC000003874185 |
| Mefruside | ZINC000000601302, ZINC000002029526 |
| Meglitinide | ZINC000000001668 |
| Meladrazine | ZINC000005650290 |
| Melagatran | ZINC000003809827 |
| Melatonin | ZINC000000057060 |
| Melitracen | ZINC000001482101 |
| Melizame | ZINC000030691055 |
| Meloxicam | ZINC000000537969, ZINC000003825290 |
| Melperone | ZINC000000001672 |
| Melquinast | ZINC000000004467 |
| Meluadrine | ZINC000000004904 |
| Memantine | ZINC000003812933 |
| Memotine | ZINC000000001674 |
| Menadiol-sulfuric-acid | ZINC000001562166 |
| Menadione-sulfurous-acid | ZINC000001999508, ZINC000001999509 |
| Menbutone | ZINC000000000446 |
| Menglytate | ZINC000038653048, ZINC000038653057 |
| Menitrazepam | ZINC000004216842 |
| Menthyl_salicylate | ZINC000000002060, ZINC000002584369, ZINC000003875616, ZINC000003875617, ZINC000008602756, ZINC000013831815 |
| Mepazine | ZINC000003683831, ZINC000003683833 |
| Mepenzolate | ZINC000000000449, ZINC000003813083 |
| Meperidine | ZINC000000001681 |
| Mephenesin-carbamate | ZINC000000000451, ZINC000001678403 |
| Mephentermine | ZINC000008132748 |
| Mephobarbital | ZINC000095660919, ZINC000095671365 |
| Mepindolol | ZINC000000004354, ZINC000001999267 |
| Mepiperphenidol | ZINC000000000455, ZINC000001747999, ZINC000001748000, ZINC000001748002 |
| Mepiprazole | ZINC000000001682 |
| Mepivacaine | ZINC000000000456, ZINC000000154964 |
| Mepixanox | ZINC000004216861 |
| Mepramidil | ZINC000004213776 |

Table S1. (continued next page)

| Drug name | ZINC ID |
| --- | --- |
| Meprotixol | ZINC000001999517, ZINC000004626657 |
| Meprylcaine | ZINC000002040457 |
| Meptazinol | ZINC000000000460, ZINC000000000854 |
| Mequidox | ZINC000001582261 |
| Mequitamium | ZINC000000005659, ZINC000001842757 |
| Meradimate | ZINC000000056987, ZINC000001162003, ZINC000003999653, ZINC000004213771 |
| Merafloxacin | ZINC000004213781, ZINC000005139696 |
| Merestinib | ZINC000095926668 |
| Mergocriptine | ZINC000072266905 |
| Meribendan | ZINC000100378338, ZINC000100378342 |
| Mericitabine | ZINC000035307932 |
| Merimepodib | ZINC000003975663 |
| Meseclazone | ZINC000000000463, ZINC000001869524, ZINC000004213787, ZINC000005573063 |
| Mesocarb | ZINC000005735333, ZINC000005751608 |
| Meso-hexestrol | ZINC000000056546, ZINC000000056547, ZINC000000056549 |
| Mesoridazine | ZINC000003831041, ZINC000003831042 |
| Mespiperone | ZINC000002572278 |
| Mesulergine | ZINC000004216874 |
| Mesulfamide | ZINC000002156420 |
| Mesulfen | ZINC000000001690 |
| Metabromsalan | ZINC000000537985 |
| Metabutethamine | ZINC000001672766 |
| Metaclazepam | ZINC000000601234, ZINC000001999451 |
| Metahexamide | ZINC000003831047 |
| Metalol | ZINC000000000465, ZINC000056897632, ZINC000056897633, ZINC000056897635 |
| Metamfazone | ZINC000000001693 |
| Metanixin | ZINC000000001694 |
| Metapramine | ZINC000002019958, ZINC000004626658 |
| Metaproterenol | ZINC000000000469, ZINC000000002273 |
| Metaxalone | ZINC000000000471, ZINC000000897322 |
| Metazamide | ZINC000000000472 |
| Metbufen | ZINC000000000474, ZINC000001846221 |
| Metcaraphen | ZINC000004216881 |
| Metergoline | ZINC000003812975 |
| Metergotamine | ZINC000072266819 |
| Metesculetol | ZINC000000001696 |
| Metesind | ZINC000000537989 |
| Metethoheptazine | ZINC000004626659, ZINC000005857177 |
| Metformin | ZINC000012859773 |
| Methadyl-acetate | ZINC000001530967, ZINC000002007678, ZINC000002007680, ZINC000002007682 |
| Methallenestril | ZINC000000000478, ZINC000000897074 |
| Methalthiazide | ZINC000001582262, ZINC000004213810 |
| Methantheline | ZINC000001530932 |
| Methaphenilene | ZINC000000001699 |
| Methaqualone | ZINC000002568176 |
| Methastyridone | ZINC000000000479, ZINC000001678603 |
| Methazolamide | ZINC000100019188 |
| Methdilazine | ZINC000000000480, ZINC000000896683 |
| Methenamine | ZINC000086040406 |
| Metheptazine | ZINC000004213813, ZINC000033852228 |

Table S1. (continued next page)

| Drug name | ZINC ID |
| --- | --- |
| Methestrol_dipropionate | ZINC000002565817, ZINC000003875568, ZINC000003875569 |
| Methestrol | ZINC000001481875, ZINC000001999322, ZINC000001999323 |
| Methixene | ZINC000001481877, ZINC000001713960 |
| Methocarbamol | ZINC000000057340, ZINC000000057341 |
| Methoserpidine | ZINC000004213820 |
| Methotrexate | ZINC000001529323 |
| Methoxamine | ZINC000000057406, ZINC000000057407, ZINC000000057408, ZINC000000057409 |
| Methoxypromazine | ZINC000000000488 |
| Methylatropine | ZINC000100371013, ZINC000100371018 |
| Methylbenactyzium | ZINC000001718373 |
| Methylbenzethonium | ZINC000002167075 |
| Methyldesorphine | ZINC000004216904 |
| Methyldihydromorphine | ZINC000004216906 |
| Methylene-blue | ZINC000012414057 |
| Methylergonovine | ZINC000095619105 |
| Methylhomatropine | ZINC000100012628, ZINC000100012630 |
| Methylnaltrexone | ZINC000005764759 |
| Methylphenidate | ZINC000000001267, ZINC000000896709, ZINC000002522648 |
| Methylpromazine | ZINC000000000808, ZINC000000896681 |
| Methylrosaniline | ZINC000003878154 |
| Methysergide | ZINC000053151228 |
| Metiapine | ZINC000056897544 |
| Metiazinic-acid | ZINC000000001717 |
| Metibride | ZINC000004216912 |
| Meticrane | ZINC000000001718 |
| Metindizate | ZINC000169292417 |
| Metioprim | ZINC000000001719 |
| Metioxate | ZINC000000537994 |
| Metipranolol | ZINC000000000494, ZINC000000389149 |
| Metiprenaline | ZINC000000000495, ZINC000001846037 |
| Metitepine | ZINC000019362650, ZINC000019362651 |
| Metizoline | ZINC000000001721 |
| Metkephamid | ZINC000056897636 |
| Metocinium | ZINC000001999358 |
| Metoclopramide | ZINC000001530716 |
| Metofenazate | ZINC000022463067 |
| Metolazone | ZINC000000601254, ZINC000000896755 |
| Metomidate | ZINC000000000496 |
| Metopimazine | ZINC000000537996 |
| Metopon | ZINC000003875958 |
| Metoprine | ZINC000000001723 |
| Metoprolol | ZINC000001530717, ZINC000001530718 |
| Metoquizine | ZINC000000607966, ZINC000038424542 |
| Metoserpate | ZINC000004213849 |
| Metostilenol | ZINC000033956908, ZINC000033956909 |
| Metoxepin | ZINC000031771497 |
| Metrafazoline | ZINC000000001725 |
| Metralindole | ZINC000100011469 |
| Metrazifone | ZINC000000001726 |
| Metrenperone | ZINC000000537997 |

Table S1. (continued next page)

| Drug name | ZINC ID |
| --- | --- |
| Metrifudil | ZINC000004216929 |
| Metronidazole_phosphate | ZINC000001855987 |
| Mevastatin | ZINC000003833876 |
| Mexafylline | ZINC000000000499, ZINC000004213852 |
| Mexazolam | ZINC000040163146, ZINC000040163147, ZINC000040163148, ZINC000040163149 |
| Mexenone | ZINC000000000500 |
| Mexoprofen | ZINC000004213855, ZINC000038418475 |
| Mezacopride | ZINC000003781049, ZINC000004626661 |
| Mezepine | ZINC000004216933 |
| Mezilamine | ZINC000000001729 |
| Mianserin | ZINC000000000504 |
| Mibampator | ZINC000000593414 |
| Mibefradil | ZINC000003782486 |
| Micinicate | ZINC000013884306, ZINC000013884308 |
| Midafotel | ZINC000022441635 |
| Midaglizole | ZINC000000000505, ZINC000001846167 |
| Midamaline | ZINC000005957780 |
| Midaxifylline | ZINC000000006513 |
| Midazogrel | ZINC000001999481, ZINC000013209374 |
| Midazolam | ZINC000095626706 |
| Midodrine | ZINC000000000507, ZINC000000403079 |
| Midostaurin | ZINC000100013130 |
| Mifentidine | ZINC000001999466 |
| Miglitol | ZINC000000897171, ZINC000003831131, ZINC000003831132, ZINC000003831133, ZINC000003831134, ZINC000004097426, ZINC000004165550, ZINC000004165583, ZINC000004165606, ZINC000011592756, ZINC000033961778, ZINC000039937314, ZINC000044021593, ZINC000044168948, ZINC000141935332 |
| Milacainide | ZINC000000004776 |
| Milacemide | ZINC000001854088 |
| Milameline | ZINC000027638049 |
| Milciclib | ZINC000053119602 |
| Milenperone | ZINC000000538006 |
| Milfasartan | ZINC000001539347 |
| Milipertine | ZINC000004213864 |
| Milnacipran | ZINC000000000506, ZINC000000014037, ZINC000001846944, ZINC000005649654 |
| Miloxacin | ZINC000000001734 |
| Milverine | ZINC000001482110 |
| Mimbane | ZINC000004213866 |
| Minalrestat | ZINC000000597385, ZINC000001492564 |
| Minaprine | ZINC000019796082 |
| Mindodilol | ZINC000001846111, ZINC000004213867 |
| Mindoperone | ZINC000004213869 |
| Minepentate | ZINC000001842997 |
| Minocromil | ZINC000000538010 |
| Minoxidil | ZINC000000001735 |
| Mioflazine | ZINC000022463073, ZINC000022851772 |
| Mipitroban | ZINC000000538012 |
| Miproxifene | ZINC000001890143 |
| Mirabegron | ZINC000001996784, ZINC000044136504 |
| Mirfentanil | ZINC000000538015 |

Table S1. (continued next page)

| Drug name | ZINC ID |
| --- | --- |
| Mirodenafil | ZINC000575440622, ZINC000575440623, ZINC000575440624, ZINC000575440625 |
| Mirogabalin | ZINC000113907768, ZINC000113907772, ZINC000113907773, ZINC000113907790, ZINC000117200300, ZINC000203566954 |
| Miroprofen | ZINC000000000508, ZINC000001846301 |
| Mirtazapine | ZINC000000000509, ZINC000000968310 |
| Mitiglinide | ZINC000001482913 |
| Mitoflaxone | ZINC000000001453 |
| Mitonafide | ZINC000003798955 |
| Mitratapide | ZINC000003946578 |
| Mivazerol | ZINC000004674535 |
| Mivobulin | ZINC000000004088 |
| Mizolastine | ZINC000013831810 |
| Mizoribine | ZINC000003812887 |
| Mk-0249 | ZINC000038269036 |
| Mk-0533 | ZINC000049878561 |
| Mk-0893 | ZINC000068250425, ZINC000095574316 |
| Mk-2206 | ZINC000036382821 |
| Mk-2461 | ZINC000034842284, ZINC000034842286 |
| Mk3207 | ZINC000043203371, ZINC000103760978, ZINC000103760981, ZINC000103760984 |
| Mk-5108 | ZINC000073069245, ZINC000100015580, ZINC000252670601 |
| Mk-6592 | ZINC000043153387 |
| Mk-7246 | ZINC000034804182 |
| Mk-8033 | ZINC000068203670 |
| Mln-0128 | ZINC000073069271 |
| Mln-2480 | ZINC000043202463, ZINC000043202464 |
| Mln-8054 | ZINC000018825334 |
| Mobecarb | ZINC000031771029 |
| Mobenzoxamine | ZINC000019361072, ZINC000022942298 |
| Mocetinostat | ZINC000013986811 |
| Moclobemide | ZINC000019606670 |
| Modecainide | ZINC000000597290, ZINC000003802769 |
| Moexiprilat | ZINC000003789196 |
| Moexipril | ZINC000003812306 |
| Mofarotene | ZINC000027558006 |
| Mofezolac | ZINC000000003767 |
| Mofloverine | ZINC000031490525 |
| Mofoxime | ZINC000005179070 |
| Moguisteine | ZINC000000003986, ZINC000002015997 |
| Molinazone | ZINC000000001746 |
| Molindone | ZINC000022002214, ZINC000022002218 |
| Molracetam | ZINC000036046324 |
| Molsidomine | ZINC000018117797 |
| Monalazone | ZINC000008576128 |
| Monatepil | ZINC000000607979, ZINC000001851142 |
| Monometacrine | ZINC000001662270 |
| Monophosphothiamine | ZINC000001532839 |
| Montelukast | ZINC000003831151 |
| Moperone | ZINC000000538026 |
| Mopidamol | ZINC000000538030 |
| Mopidralazine | ZINC000000001749 |

Table S1. (continued next page)

| Drug name | ZINC ID |
| --- | --- |
| Moprolol | ZINC000000000513, ZINC000001999512 |
| Moquizone | ZINC000031290966 |
| Morantel | ZINC000000001750 |
| Morazone | ZINC000017106663, ZINC000031982610, ZINC000031982612, ZINC000031982616 |
| Morclofone | ZINC000030691542 |
| Morforex | ZINC000022463079, ZINC000033852250 |
| Moricizine | ZINC000019340795 |
| Morinamide | ZINC000021673450 |
| Morniflumate | ZINC000021999791 |
| Morocromen | ZINC000072266844 |
| Morpheridine | ZINC000022463259 |
| Morphine | ZINC000000403034, ZINC000003628643, ZINC000003812983 |
| Morsuximide | ZINC000031297920, ZINC000031297922 |
| Mosapramine | ZINC000000597691, ZINC000003873789 |
| Mosapride | ZINC000020621755, ZINC000020621758 |
| Motapizone | ZINC000000000517, ZINC000004213902 |
| Motesanib | ZINC000018710082 |
| Motrazepam | ZINC000004216987 |
| Moveltipril | ZINC000004216989 |
| Moxaprindine | ZINC000001846361, ZINC000005316022 |
| Moxastine | ZINC000000001753 |
| Moxaverine | ZINC000000001754 |
| Moxazocine | ZINC000004213908 |
| Moxicoumone | ZINC000031291708 |
| Moxifloxacin | ZINC000003826253 |
| Moxiraprine | ZINC000000001756 |
| Moxisylyte | ZINC000000057401 |
| Moxnidazole | ZINC000017108685, ZINC000031291669 |
| Moxonidine | ZINC000001854466 |
| Mozavaptan | ZINC000003804565, ZINC000006071859 |
| Msx-122 | ZINC000038247771 |
| Mubritinib | ZINC000011679877 |
| Muraglitazar | ZINC000049650290 |
| Muzolimine | ZINC000000000521, ZINC000002002765 |
| Mycophenolate-mofetil | ZINC000021297660, ZINC000022064264 |
| Mycophenolic-acid | ZINC000000001758, ZINC000002014826 |
| Myfadol | ZINC000001999515, ZINC000005736133 |
| Myrtecaine | ZINC000001843013 |
| Nabazenil | ZINC000031475739, ZINC000031475748, ZINC000040163159, ZINC000040163161 |
| Nadifloxacin | ZINC000000603195, ZINC000003794622 |
| Nadolol | ZINC000001530731, ZINC000003812974, ZINC000003831165, ZINC000003831166, ZINC000011592715 |
| Nadoxolol | ZINC000004213923, ZINC000027644510 |
| Nafagrel | ZINC000028762844, ZINC000028762846 |
| Nafamostat | ZINC000003874467 |
| Nafazatrom | ZINC000000001764 |
| Nafcaproic-acid | ZINC000000001765 |
| Nafenopin | ZINC000000000526, ZINC000000967790 |
| Nafetolol | ZINC000000000527, ZINC000001846404 |
| Nafimidone | ZINC000000001766 |

Table S1. (continued next page)

| Drug name | ZINC ID |
| --- | --- |
| Nafiverine | ZINC000022463090, ZINC000072266771, ZINC000072266772 |
| Nafomine | ZINC000000001767 |
| Nafoxadol | ZINC000000001768 |
| Nafoxidine | ZINC000000538045 |
| Nafronyl | ZINC000003831171, ZINC000003831172, ZINC000003831173, ZINC000003831174 |
| Naftalofos | ZINC000001759150 |
| Naftifine | ZINC000001530977 |
| Naftopidil | ZINC000019632696, ZINC000019632700 |
| Naftypramide | ZINC000001846033, ZINC000004213933 |
| Nalbuphine | ZINC000003812989 |
| Nalidixic-acid | ZINC000000057421 |
| Nalmefene | ZINC000000403529 |
| Nalmexone | ZINC000004217034 |
| Nalorphine | ZINC000003875420 |
| Naloxone | ZINC000000389747 |
| Naltrexone | ZINC000000001773 |
| Naluzotan | ZINC000003963096 |
| Naminterol | ZINC000000000529, ZINC000005141553, ZINC000005141554, ZINC000005141742 |
| Namirotene | ZINC000000001774 |
| Nanterinone | ZINC000000001778 |
| Nantradol | ZINC000004213937, ZINC000030691106 |
| Napamezole | ZINC000000001780 |
| Naphazoline | ZINC000000119717 |
| Naphthonone | ZINC000000000530, ZINC000001999296 |
| Napirimus | ZINC000000001783 |
| Napitane | ZINC000000005009, ZINC000003794361, ZINC000005934263 |
| Naprodoxime | ZINC000004213940, ZINC000006019931 |
| Naproxcinod | ZINC000001541804 |
| Naproxen | ZINC000000105216 |
| Naproxol | ZINC000002013137 |
| Napsagatran | ZINC000003807298 |
| Naranol | ZINC000011616846 |
| Naratriptan | ZINC000000004076 |
| Narcobarbital | ZINC000002027185, ZINC000004213943 |
| Nardeterol | ZINC000001846070, ZINC000003782856 |
| Nateglinide | ZINC000100015346, ZINC000100067350 |
| Naveglitazar | ZINC000003950327 |
| Navitoclax | ZINC000150338726 |
| Naxagolide | ZINC000022064446 |
| Naxaprostene | ZINC000004217054 |
| Nebidrazine | ZINC000004217062 |
| Nebivolol | ZINC000004213946, ZINC000005844792, ZINC000011681534 |
| Neboglamine | ZINC000000006138 |
| Nebracetam | ZINC000002016002, ZINC000004636519 |
| Necopidem | ZINC000000538062 |
| Nedocromil | ZINC000003782807 |
| Nefazodone | ZINC000000538065 |
| Nefiracetam | ZINC000000003788 |
| Neflumozide | ZINC000000538067 |

Table S1. (continued next page)

| Drug name | ZINC ID |
| --- | --- |
| Nefopam | ZINC000000120181, ZINC000000120186 |
| Nelarabine | ZINC000000006508, ZINC000001693850, ZINC000003823492, ZINC000004758477, ZINC000004758478, ZINC000004758479, ZINC000004758480, ZINC000005998706, ZINC000022063177, ZINC000097975521 |
| Neldazosin | ZINC000000607988, ZINC000002016010 |
| Nelezaprine | ZINC000000001789 |
| Nelfinavir | ZINC000003833846 |
| Nelivaptan | ZINC000042833251 |
| Nelotanserin | ZINC000038239930 |
| Nemazoline | ZINC000000001790 |
| Nemonapride | ZINC000000538069, ZINC000002559936 |
| Nemonoxacin | ZINC000040435195 |
| Neocinchophen | ZINC000000001791 |
| Neomycin | ZINC000071928291 |
| Neostigmine | ZINC000000001792 |
| Nepafenac | ZINC000005162311 |
| Nepaprazole | ZINC000001545074, ZINC000004626669, ZINC000004626670, ZINC000008463351 |
| Nepinalone | ZINC000003775017, ZINC000013888770 |
| Nequinate | ZINC000100033029 |
| Neramexane | ZINC000004217734 |
| Neraminol | ZINC000033852252, ZINC000033852253 |
| Nerispirdine | ZINC000000005498 |
| Nesapidil | ZINC000026892447, ZINC000026892453 |
| Nesbuvir | ZINC000030691787 |
| Nesosteine | ZINC000000001794 |
| Nestifylline | ZINC000000003885 |
| Neticonazole | ZINC000013889772 |
| Netilmicin | ZINC000052981502 |
| Netoglitazone | ZINC000001481955, ZINC000003633833 |
| Netupitant | ZINC000011681563 |
| Nevirapine | ZINC000000004778 |
| Nexeridine | ZINC000004213958, ZINC000038223828 |
| Niaprazine | ZINC000004213960, ZINC000005141698 |
| Nicafenine | ZINC000001846209 |
| Nicainoprol | ZINC000001999558, ZINC000004213962 |
| Nicametate | ZINC000001678387 |
| Nicanartine | ZINC000001536279 |
| Nicaraven | ZINC000001817747, ZINC000001817748 |
| Nicergoline | ZINC000003873817 |
| Niceritrol | ZINC000003831199 |
| Niceverine | ZINC000001481885 |
| Niclosamide | ZINC000003874496 |
| Nicoboxil | ZINC000002020020 |
| Nicoclonate | ZINC000000000538, ZINC000001843012 |
| Nicodicodine | ZINC000004217090 |
| Nicofetamide | ZINC000014684144, ZINC000014684145 |
| Nicofibrate | ZINC000000001796 |
| Nicofuranose | ZINC000004217093 |
| Nicofurate | ZINC000072266767, ZINC000072266769 |
| Nicogrelate | ZINC000004213973, ZINC000005114640 |

Table S1. (continued next page)

| Drug name | ZINC ID |
| --- | --- |
| Nicomol | ZINC000004213975 |
| Nicoracetam | ZINC000033852254 |
| Nicorandil | ZINC000001533102 |
| Nicotine | ZINC000000391812 |
| Nicotredole | ZINC000000004164 |
| Nictiazem | ZINC000004217098 |
| Nictindole | ZINC000000001801 |
| Nidroxyzone | ZINC000001482117 |
| Nifekalant | ZINC000000538083 |
| Nifenalol | ZINC000000000539, ZINC000000283862 |
| Nifenazone | ZINC000000057417 |
| Niflumic-acid | ZINC000000125031 |
| Nifluridide | ZINC000003809180 |
| Nifuradene | ZINC000000001803 |
| Nifuraldezone | ZINC000001666628 |
| Nifuralide | ZINC000004217102 |
| Nifuratel | ZINC000000000540, ZINC000002037496 |
| Nifuratrone | ZINC000001567412 |
| Nifurdazil | ZINC000000001804 |
| Nifurethazone | ZINC000000001805 |
| Nifurfoline | ZINC000038609700 |
| Nifurimide | ZINC000000000541, ZINC000002016125 |
| Nifurmazole | ZINC000001842732 |
| Nifuroquine | ZINC000001842848 |
| Nifuroxazide | ZINC000000001808 |
| Nifurpipone | ZINC000000001809 |
| Nifurpirinol | ZINC000004217117 |
| Nifurprazine | ZINC000004217120 |
| Nifurquinazol | ZINC000000001810 |
| Nifursemizone | ZINC000000001811 |
| Nifurtimox | ZINC000000000542, ZINC000000968362 |
| Nifurtoinol | ZINC000001482118 |
| Nifurvidine | ZINC000008144574 |
| Nifurzide | ZINC000001542910 |
| Nikethamide | ZINC000000001814 |
| Nilotinib | ZINC000006716957 |
| Nilprazole | ZINC000022463265 |
| Nilutamide | ZINC000003874498 |
| Nimesulide | ZINC000004617749 |
| Nimetazepam | ZINC000003831205 |
| Nimidane | ZINC000000001816 |
| Nimorazole | ZINC000026167988 |
| Niometacin | ZINC000000000544 |
| Niperotidine | ZINC000030691406 |
| Nipradilol | ZINC000003812911, ZINC000005442134, ZINC000005442135, ZINC000005442195 |
| Niprofazone | ZINC000000538089 |
| Niraparib | ZINC000043206370 |
| Niravoline | ZINC000000607401 |
| Niridazole | ZINC000003875416 |
| Nirogacestat | ZINC000038217835, ZINC000038217836, ZINC000038217837, ZINC000038217838 |

Table S1. (continued next page)

| Drug name | ZINC ID |
| --- | --- |
| Nisbuterol | ZINC000004213979, ZINC000011681591 |
| Nisoxetine | ZINC000001849595, ZINC000001849597 |
| Nitazoxanide | ZINC000003956788 |
| Nitisinone | ZINC000100014475 |
| Nitracrine | ZINC000003812939 |
| Nitrafudam | ZINC000000001818 |
| Nitralamine | ZINC000001583514, ZINC000002039570 |
| Nitramisole | ZINC000000000547, ZINC000004213983 |
| Nitraquazone | ZINC000000001819 |
| Nitrazepam | ZINC000004311748 |
| Nitrazepate | ZINC000004214169, ZINC000005850731 |
| Nitrefazole | ZINC000001842965 |
| Nitroblue-tetrazolium | ZINC000008214433 |
| Nitromide | ZINC000001690424 |
| Nitroxazepine | ZINC000002018449 |
| Nixylic-acid | ZINC000000001824 |
| Nizofenone | ZINC000000538096 |
| Nms-1286937 | ZINC000049113058 |
| Noberastine | ZINC000000003719 |
| Nocloprost | ZINC000038192483 |
| Nocodazole | ZINC000000056509 |
| Nofecainide | ZINC000001846387, ZINC000001846388, ZINC000001846389, ZINC000004626672 |
| Nolatrexed | ZINC000000008107 |
| Nolinium | ZINC000000001826 |
| Nolomirole | ZINC000003598367, ZINC000004634613 |
| Nolpitantium | ZINC000003922699 |
| Nomelidine | ZINC000001846248 |
| Nomifensine | ZINC000019796033, ZINC000019796035 |
| Nonabine | ZINC000004213992, ZINC000005315476, ZINC000005315486, ZINC000005315490 |
| Nonaperone | ZINC000000001828 |
| Noracymethadol | ZINC000002575722, ZINC000005651045, ZINC000005651368, ZINC000005651370 |
| Norbudrine | ZINC000000000551, ZINC000006092147 |
| Norcodeine | ZINC000004217170 |
| Nordazepam | ZINC000001249069 |
| Norfloxacin-succinil | ZINC000000538102 |
| Norfloxacin | ZINC000000003742 |
| Norletimol | ZINC000100091581 |
| Norlevorphanol | ZINC000001482126 |
| Normorphine | ZINC000004102208 |
| Nortetrazepam | ZINC000000001838 |
| Nortopixantrone | ZINC000001538986 |
| Novobiocin | ZINC000076945632 |
| Noxiptiline | ZINC000001999308 |
| Nrx195183 | ZINC000001909562 |
| Nuclomedone | ZINC000000000555, ZINC000004214008 |
| Nuclotixene | ZINC000031477134, ZINC000031477144 |
| Nufenoxole | ZINC000004217195 |
| Nupafant | ZINC000006145655 |
| Nuvenzepine | ZINC000000001845 |
| Nvp-auy922 | ZINC000100015656 |

Table S1. (continued next page)

| Drug name | ZINC ID |
| --- | --- |
| Nylidrin | ZINC000000057422, ZINC000008575811, ZINC000009212278, ZINC000011616368 |
| Obatoclax | ZINC000029052268 |
| Oc000459 | ZINC000043120334 |
| Ocaperidone | ZINC000000538119 |
| Ocfentanil | ZINC000000538120 |
| Ocinaplon | ZINC000000005014 |
| Octafonium | ZINC000004214019 |
| Octapinol | ZINC000001846093 |
| Octastine | ZINC000004214026, ZINC000006067238 |
| Octaverine | ZINC000001574926 |
| Octazamide | ZINC000000000557, ZINC000002002432, ZINC000002002433 |
| Octicizer | ZINC000002039679, ZINC000002039680 |
| Octisalate | ZINC000001677818, ZINC000002041028 |
| Octodrine | ZINC000001481887, ZINC000001634312 |
| Octriptyline | ZINC000002005382, ZINC000002005385, ZINC000002005386 |
| Octrizole | ZINC000001849911 |
| Odalprofen | ZINC000003777867, ZINC000013898175, ZINC000013898178, ZINC000013898181 |
| Odapipam | ZINC000003781602 |
| Odiparcil | ZINC000031597280 |
| Ofloxacin | ZINC000000537891, ZINC000000538273 |
| Ofornine | ZINC000000001848 |
| Oglufanide | ZINC000001576164, ZINC000001576165, ZINC000001576166, ZINC000001576167 |
| Olamufloxacin | ZINC000003811114 |
| Olanzapine | ZINC000052957434 |
| Olaparib | ZINC000040430143 |
| Olaquindox | ZINC000001624227 |
| Olcegepant | ZINC000098052868 |
| Oliceridine | ZINC000096940334, ZINC000145060717 |
| Olmesartan_medoxomil | ZINC000004149248 |
| Olmesartan | ZINC000000538621 |
| Olmidine | ZINC000000000559, ZINC000001842959 |
| Olodaterol | ZINC000034636383 |
| Olopatadine | ZINC000000001850 |
| Olsalazine | ZINC000003812865 |
| Omaciclovir | ZINC000000005233 |
| Omarigliptin | ZINC000084758480 |
| Ombitasvir | ZINC000150601177 |
| Omecamtiv-mecarbil | ZINC000038253214 |
| Omidoline | ZINC000002019959, ZINC000005318621 |
| Omipalisib | ZINC000043208634 |
| Omoconazole | ZINC000001532917 |
| Onalespib | ZINC000043208226 |
| Ondansetron | ZINC000000004448, ZINC000000075126 |
| Ontazolast | ZINC000000005761 |
| Opanixil | ZINC000084758648 |
| Opaviraline | ZINC000003916138 |
| Opiniazide | ZINC000000001855 |
| Opipramol | ZINC000021981303 |
| Orbifloxacin | ZINC000003882891 |
| Orbofiban | ZINC000003951736 |

Table S1. (continued next page)

| Drug name | ZINC ID |
| --- | --- |
| Orbutopril | ZINC000038174251 |
| Orconazole | ZINC000000608006, ZINC000005117064 |
| Ordopidine | ZINC000038277193 |
| Ormeloxifene | ZINC000001730387, ZINC000001730388, ZINC000001730389, ZINC000005104028 |
| Ormetoprim | ZINC000000000562 |
| Orotirelin | ZINC000004217208 |
| Orpanoxin | ZINC000000000564, ZINC000002001091 |
| Orphenadrine | ZINC000000000565, ZINC000000155269 |
| Orteronel | ZINC000003943521 |
| Orvepitant | ZINC000056898864 |
| Osanetant | ZINC000003935475 |
| Osemozotan | ZINC000001552292, ZINC000003799319 |
| Osi-027 | ZINC000068203220, ZINC000252286769 |
| Osi-632 | ZINC000003834191 |
| Osi-930 | ZINC000003962535 |
| Osutidine | ZINC000011616140, ZINC000011616142 |
| Otamixaban | ZINC000001908051 |
| Otenabant | ZINC000003948997 |
| Otenzepad | ZINC000022454198, ZINC000022454203 |
| Oxabrexine | ZINC000000538132 |
| Oxadimedine | ZINC000000001857 |
| Oxaflozane | ZINC000030691499, ZINC000030691504 |
| Oxaflumazine | ZINC000022463271 |
| Oxagrelate | ZINC000017109538 |
| Oxalinast | ZINC000004214052, ZINC000006092280 |
| Oxamarin | ZINC000000538134 |
| Oxametacin | ZINC000000608008 |
| Oxamisole | ZINC000000000569, ZINC000002021122 |
| Oxamniquine | ZINC000000000570, ZINC000000896836 |
| Oxantel | ZINC000100025503 |
| Oxapadol | ZINC000000001862, ZINC000001846290 |
| Oxapium | ZINC000000000572, ZINC000003874575, ZINC000003874576, ZINC000003874577 |
| Oxaprozin | ZINC000049643479 |
| Oxarbazole | ZINC000000000574, ZINC000002008674 |
| Oxatomide | ZINC000019632896 |
| Oxazafone | ZINC000001846108 |
| Oxazepam | ZINC000000000575, ZINC000000509440 |
| Oxazidione | ZINC000031391374 |
| Oxazolam | ZINC000077312063, ZINC000077312066 |
| Oxazorone | ZINC000024177975 |
| Oxcarbazepine | ZINC000000004724 |
| Oxdralazine | ZINC000000001866 |
| Oxeladin | ZINC000003874580 |
| Oxepinac | ZINC000000001867 |
| Oxethazaine | ZINC000003874585 |
| Oxetorone | ZINC000000001868 |
| Oxfendazole | ZINC000008584337, ZINC000008584338 |
| Oxibendazole | ZINC000004685859 |
| Oxiconazole | ZINC000003873295 |
| Oxifungin | ZINC000000001870 |

Table S1. (continued next page)

| Drug name | ZINC ID |
| --- | --- |
| Oxilorphan | ZINC000004217226 |
| Oximonam | ZINC000004217227 |
| Oxindanac | ZINC000000000578, ZINC000001846144 |
| Oxiperomide | ZINC000000001873 |
| Oxiramide | ZINC000004214064, ZINC000011681740 |
| Oxitefonium | ZINC000001842969, ZINC000001842971 |
| Oxitriptan | ZINC000000895330, ZINC000000895459 |
| Oxitriptyline | ZINC000000000580 |
| Oxmetidine | ZINC000008144601 |
| Oxolamine | ZINC000000001874 |
| Oxolinic-acid | ZINC000000001875 |
| Oxomemazine | ZINC000000000582, ZINC000002038507 |
| Oxonazine | ZINC000000001876 |
| Oxpheneridine | ZINC000004214073, ZINC000005650539 |
| Oxprenolol | ZINC000001542924, ZINC000001999544 |
| Oxycinchophen | ZINC000000001877 |
| Oxycodone | ZINC000000403533 |
| Oxyfedrine | ZINC000002018339 |
| Oxymetazoline | ZINC000000057435 |
| Oxymorphone | ZINC000003875483 |
| Oxypendyl | ZINC000022463283 |
| Oxypertine | ZINC000000538144 |
| Oxyphenbutazone | ZINC000001606505, ZINC000002041017 |
| Oxyphencyclimine | ZINC000000020260, ZINC000000057439 |
| Oxyphenisatin_acetate | ZINC000003831258 |
| Oxyphenisatine | ZINC000004217234 |
| Oxyphenonium | ZINC000001812122, ZINC000001812123 |
| Oxypurinol | ZINC000084462581 |
| Oxypyrronium | ZINC000000000585, ZINC000001999362, ZINC000001999363, ZINC000001999364 |
| Oxyridazine | ZINC000000608012, ZINC000002020011 |
| Oxysonium | ZINC000004214079, ZINC000005764906 |
| Ozenoxacin | ZINC000001483896 |
| Pa-799 | ZINC000066074200 |
| Padimate-o | ZINC000002016257, ZINC000002016258 |
| Pafenolol | ZINC000000004779, ZINC000001999561 |
| Palatrigine | ZINC000004217236 |
| Palbociclib | ZINC000003938686 |
| Palinavir | ZINC000003936474 |
| Paliperidone | ZINC000001481956, ZINC000004214700 |
| Paliroden | ZINC000035826853 |
| Palomid-529 | ZINC000043195311, ZINC000043195312 |
| Palonosetron | ZINC000003795819 |
| Palosuran | ZINC000034375693 |
| Palovarotene | ZINC000038467831 |
| Pamabrom | ZINC000100005670 |
| Pamapimod | ZINC000030691792 |
| Pamaqueside | ZINC000257972190 |
| Pamaquine-naphthoate | ZINC000001655461, ZINC000001996070 |
| Pamatolol | ZINC000000000586, ZINC000001842716 |
| Pamicogrel | ZINC000000596743 |

Table S1. (continued next page)

| Drug name | ZINC ID |
| --- | --- |
| Panadiplon | ZINC000000004220 |
| Panamesine | ZINC000003801505 |
| Pancopride | ZINC000000000587, ZINC000001999426 |
| Panidazole | ZINC000000001882 |
| Panomifene | ZINC000001853830 |
| Papaverine | ZINC000000056555 |
| Paraflutizide | ZINC000000608014, ZINC000001843075 |
| Para-nitrosulfathiazole | ZINC000000198656 |
| Paranyline | ZINC000001482136 |
| Paraoxon | ZINC000001530487 |
| Parapenzolate | ZINC000000001887 |
| Parbendazole | ZINC000005424253 |
| Parcetasal | ZINC000000014940, ZINC000002019985 |
| Pardoprunox | ZINC000000008736 |
| Parecoxib | ZINC000005761797 |
| Pareptide | ZINC000004217244 |
| Parethoxycaine | ZINC000001833029 |
| Paridocaine | ZINC000002020819 |
| Parodilol | ZINC000003779694, ZINC000013884221 |
| Paromomycin | ZINC000060183170 |
| Paroxetine | ZINC000000527386 |
| Paroxypropione | ZINC000000001890 |
| Patamostat | ZINC000005840106 |
| Paxamate | ZINC000000001892 |
| Pazelliptine | ZINC000001551834 |
| Pazopanib | ZINC000011617039 |
| Pazoxide | ZINC000001846472 |
| Pazufloxacin | ZINC000003779726 |
| P-butylaminobenzoyldiethylaminoethyl | ZINC000002018421 |
| Pd-0166285 | ZINC000001486219 |
| Pefloxacin | ZINC000000001894 |
| Pelanserin | ZINC000000538156 |
| Peldesine | ZINC000005420970 |
| Peliglitazar | ZINC000003935841 |
| Pelitrexol | ZINC000004846117 |
| Pemafibrate | ZINC000028900759, ZINC000028900764 |
| Pemaglitazar | ZINC000034228435 |
| Pemedolac | ZINC000000000590, ZINC000000005544, ZINC000001851370, ZINC000001851372 |
| Pemerid | ZINC000004217247 |
| Pemetrexed | ZINC000001851132 |
| Pemirolast | ZINC000005783214 |
| Penbutolol | ZINC000000001898 |
| Penciclovir | ZINC000000001899 |
| Penfluridol | ZINC000004217252 |
| Penflutizide | ZINC000001843070, ZINC000001843072 |
| Pentafluranol | ZINC000000000592, ZINC000033852263, ZINC000033852264, ZINC000033852265 |
| Pentalamide | ZINC000001999301 |
| Pentamoxane | ZINC000004214115, ZINC000020137760 |

Table S1. (continued next page)

| Drug name | ZINC ID |
| --- | --- |
| Pentapiperide | ZINC000000000594, ZINC000000155894, ZINC000000155896, ZINC000000155897 |
| Pentapiperium | ZINC000000000595, ZINC000000394464, ZINC000000394465, ZINC000000394466 |
| Pentazocine | ZINC000000000596, ZINC000000020242 |
| Penthienate | ZINC000001690860, ZINC000002041344 |
| Pentiapine-maleate | ZINC000030690661 |
| Pentisomide | ZINC000002016047, ZINC000002016048 |
| Pentopril | ZINC000002021801 |
| Pentosalen | ZINC000000001904 |
| Pentostatin | ZINC000003806262, ZINC000004771863, ZINC000004771864, ZINC000005975216, ZINC000008643194, ZINC000012503109, ZINC000012503112, ZINC000014649463, ZINC000026000412 |
| Peraclopone | ZINC000038811045, ZINC000038811048 |
| Peradoxime | ZINC000026892437, ZINC000026892442 |
| Perafensine | ZINC000033852266 |
| Peralopride | ZINC000026892472 |
| Peramivir | ZINC000003981610, ZINC000005819214, ZINC000006655356, ZINC000006655360, ZINC000034809511, ZINC000034809512, ZINC000059085003, ZINC000096030382, ZINC000253952094, ZINC000253952096 |
| Peraquinsin | ZINC000004214134 |
| Perastine | ZINC000001482139 |
| Peratizole | ZINC000004217268 |
| Perazine | ZINC000019362664 |
| Perbufylline | ZINC000033852267 |
| Perfomedil | ZINC000000000599, ZINC000002016061 |
| Pergolide | ZINC000003786466 |
| Perhexiline | ZINC000001999542, ZINC000001999543 |
| Perimetazine | ZINC000001849669, ZINC000004214135 |
| Perindoprilat | ZINC000004217270 |
| Perindopril | ZINC000003812867 |
| Perisoxal | ZINC000000000601, ZINC000002003697 |
| Perlapine | ZINC000027303430 |
| Permethrin | ZINC000001850374, ZINC000001850376, ZINC000001850377, ZINC000002032615 |
| Perospirone | ZINC000013828184 |
| Perphenazine | ZINC000019228902 |
| Pevonedistat | ZINC000058660702 |
| Pexacerfont | ZINC000034007762 |
| Pf-00446687 | ZINC000013983541, ZINC000095938270, ZINC000101338048, ZINC000117532710, ZINC000144234053, ZINC000147639440 |
| Pf-00477736 | ZINC000019862643, ZINC000137899389 |
| Pf-03654746 | ZINC000035285834, ZINC000100001877 |
| Pf-03654764 | ZINC000149665509 |
| Pf-03715455 | ZINC000082138051 |
| Pf-03758309 | ZINC000043203531, ZINC000222637955 |
| Pf-03893787 | ZINC000073196039 |
| Pf-04217903 | ZINC000043195316 |
| Pf-04691502 | ZINC000058660483, ZINC000100015733, ZINC000117704832 |
| Pf-562271 | ZINC000034638188 |
| Ph-797804 | ZINC000013980453 |
| Pha-543613 | ZINC000034034921, ZINC000038921400 |
| Pha-793887 | ZINC000052509437 |
| Phenacaine | ZINC000017921108 |

Table S1. (continued next page)

| Drug name | ZINC ID |
| --- | --- |
| Phenacemide | ZINC000000001916 |
| Phenallymal | ZINC000005194015 |
| Phenamazoline | ZINC000000001918 |
| Phenampromide | ZINC000000000604, ZINC000002041016 |
| Phenazocine | ZINC000014952534 |
| Phenazopyridine | ZINC000095483532 |
| phenbenzamine | ZINC000000001425 |
| Phenbutamide | ZINC000004217278 |
| Phenbutazone | ZINC000001999261 |
| Phencarbamide | ZINC000001841829 |
| Phencyclidine | ZINC000000968311 |
| Phenelzine | ZINC000019166991 |
| Pheneridine | ZINC000000001921 |
| Pheneturide | ZINC000000000274, ZINC000001842753 |
| Phenglutarimide | ZINC000005848170, ZINC000005848216 |
| Phenindamine | ZINC000000968338, ZINC000000968340 |
| Pheniprazine | ZINC000019166939, ZINC000019167667 |
| Pheniramine | ZINC000000000607, ZINC000000508068 |
| Phenolphthalol | ZINC000004217282 |
| Phenolsulfonphthalein | ZINC000003860918 |
| Phenomorphan | ZINC000004217283 |
| Phenoperidine | ZINC000004214141, ZINC000011616841 |
| Phenosulfazole | ZINC000001641309 |
| Phenothrin | ZINC000000608024, ZINC000002005534, ZINC000002013188, ZINC000002013189 |
| Phenprocoumon | ZINC000100030674, ZINC000100030679 |
| phenyl_acetylsalicylate | ZINC000000351310 |
| Phenylbutanoic_acid | ZINC000000056568 |
| Phenylbutazone | ZINC000100004227 |
| Phenyltoloxamine | ZINC000000001931 |
| Phenyramidol | ZINC000022910917, ZINC000022910921 |
| Phenytoin | ZINC000002510358 |
| Phethenylate | ZINC000005934359, ZINC000005934489 |
| Pholcodine | ZINC000004217287, ZINC000033839044, ZINC000059867903 |
| Phthalylsulfacetamide | ZINC000000538163 |
| Phthalylsulfamethizole | ZINC000033852268 |
| Phthalylsulfathiazole | ZINC000001530877 |
| Physostigmine | ZINC000053022903, ZINC000053151197, ZINC000091689892 |
| Phytonadiol-diphosphoric-acid | ZINC000004214146, ZINC000725426644 |
| Pibaxizine | ZINC000003804921 |
| Pibecarb | ZINC000000001939 |
| Piberaline | ZINC000026892463 |
| Piboserod | ZINC000001537633 |
| Picafibrate | ZINC000001846274 |
| Picenadol | ZINC000004217292, ZINC000011681924 |
| Picilorex | ZINC000031519734, ZINC000031519739 |
| Piclamilast | ZINC000000598965 |
| Piclonidine | ZINC000031297748, ZINC000031297751 |
| Piclopastine | ZINC000022463097, ZINC000033505730 |
| Picloxydine | ZINC000030691566 |

Table S1. (continued next page)

| Drug name | ZINC ID |
| --- | --- |
| Piclozotan | ZINC000001892462 |
| Picobenzide | ZINC000000001942 |
| Picodralazine | ZINC000000001943 |
| Picoperine | ZINC000000001945 |
| Picophosphoric-acid | ZINC000004217453 |
| Picoprazole | ZINC000011726795, ZINC000011726796 |
| Picosulfuric-acid | ZINC000003873921 |
| Picotamide | ZINC000000538174 |
| Picotrin | ZINC000004217296 |
| Pictilisib | ZINC000016052714 |
| Picumast | ZINC000019367677 |
| Pidolacetamol | ZINC000003797451 |
| Pidotimod | ZINC000003781245 |
| Pifenate | ZINC000000000622, ZINC000004214151 |
| Pifexole | ZINC000000001947 |
| Piflutixol | ZINC000031297681 |
| Pifoxime | ZINC000005179068 |
| Piketoprofen | ZINC000000000623, ZINC000001846243 |
| Pilaralisib | ZINC000100472223 |
| Pildralazine | ZINC000000000624, ZINC000001846213 |
| Pilocarpine | ZINC000000075008 |
| Pilsicainide | ZINC000049925459 |
| Pimavanserin | ZINC000016159083 |
| Pimefylline | ZINC000002020030 |
| Pimetacin | ZINC000000608025 |
| Pimethixene | ZINC000000000627 |
| Pimetine | ZINC000022463308 |
| Pimetremide | ZINC000000000628, ZINC000033852269 |
| Piminodine | ZINC000004217303 |
| Pimobendan | ZINC000004170129, ZINC000005071680 |
| Pimonidazole | ZINC000000000630, ZINC000001571940 |
| Pimozide | ZINC000004175630 |
| Pinafide | ZINC000004217305 |
| Pinaverium | ZINC000004214152, ZINC000022065909 |
| Pincainide | ZINC000000214987 |
| Pindolol | ZINC000000056645, ZINC000000056646 |
| Pinolcaine | ZINC000000608026, ZINC000004214153 |
| Pinoxepin | ZINC000023477086 |
| Pioglitazone | ZINC000000968326, ZINC000000968327 |
| Pipamazine | ZINC000000538183 |
| Pipamperone | ZINC000021297287 |
| Pipazethate | ZINC000001698105 |
| Pipecuronium | ZINC000003938681 |
| Pipemidic-acid | ZINC000000057466 |
| Pipendoxifene | ZINC000000602799 |
| Pipenzolate | ZINC000000601314, ZINC000003813082 |
| Pipequaline | ZINC000001482144 |
| Piperacetazine | ZINC000000538186 |
| Piperamide | ZINC000000001951 |
| Piperaquine | ZINC000026251015 |

Table S1. (continued next page)

| Drug name | ZINC ID |
| --- | --- |
| Piperidolate | ZINC000000057474, ZINC000000057476 |
| Piperilate | ZINC000000188746 |
| Piperocaine | ZINC000002034887, ZINC000002034888 |
| Piperoxan | ZINC000000000631, ZINC000001019594 |
| Piperphenidol | ZINC000000000632, ZINC000033903718, ZINC000033903719, ZINC000033903720 |
| Piperylone | ZINC000000001954 |
| Pipofezine | ZINC000000050247 |
| Pipotiazine | ZINC000003813024 |
| Pipoxizine | ZINC000004217313 |
| Pipoxolan | ZINC000000608027, ZINC000002017397 |
| Pipradimadol | ZINC000000538190 |
| Pipradrol | ZINC000000000633, ZINC000000403682 |
| Pipramadol | ZINC000004214155, ZINC000027556351 |
| Pipratecol | ZINC000031298024, ZINC000031298027 |
| Piprocurarium | ZINC000004214157, ZINC000005651730 |
| Piprofurol | ZINC000002007020, ZINC000004214158 |
| Piquindone | ZINC000000001955, ZINC000011616848, ZINC000033801265 |
| Piquizil | ZINC000000538192 |
| Piracetam | ZINC000003812874 |
| Piragliatin | ZINC000030691803 |
| Pirandamine | ZINC000000000635, ZINC000001566183 |
| Piraxelate | ZINC000000000636, ZINC000004214159, ZINC000033852271, ZINC000033852272 |
| Pirazolac | ZINC000000001957 |
| Pirbuterol | ZINC000000000637, ZINC000000897261 |
| Pirdonium | ZINC000001481899, ZINC000004214160, ZINC000033852273, ZINC000033852274 |
| Pirenoxine | ZINC000011616009 |
| Pirenperone | ZINC000000538194 |
| Pirenzepine | ZINC000019632927 |
| Piretanide | ZINC000003812930 |
| Piribedil | ZINC000019537374 |
| Piridocaine | ZINC000000000638, ZINC000001996127 |
| Pirifibrate | ZINC000000001960 |
| Pirinidazole | ZINC000000001962 |
| Pirinixic-acid | ZINC000000001963 |
| Pirinixil | ZINC000001846197 |
| Piriqualone | ZINC000004217322 |
| Pirisudanol | ZINC000002019924 |
| Piritrexim | ZINC000000000640 |
| Pirlindole | ZINC000000000641, ZINC000000347886 |
| Pirmenol | ZINC000000000642, ZINC000003873867 |
| Pirnabine | ZINC000000000644, ZINC000001843612 |
| Piroctone | ZINC000000000645, ZINC000002020088 |
| Pirodavir | ZINC000000538202 |
| Pirodomast | ZINC000100369897 |
| Pirogliride | ZINC000016155816 |
| Piroheptine | ZINC000001481900, ZINC000002020004 |
| Pirolate | ZINC000016940528 |
| Pirolazamide | ZINC000019368432 |
| Piromidic-acid | ZINC000000057461 |
| Piroximone | ZINC000000001965 |

Table S1. (continued next page)

| Drug name | ZINC ID |
| --- | --- |
| Pirozadil | ZINC000003938698 |
| Pirprofen | ZINC000000000646, ZINC000002010790 |
| Pirquinozol | ZINC000005935070 |
| Pirsidomine | ZINC000005735688 |
| Pitavastatin | ZINC000001534965 |
| Pitenodil | ZINC000022463320 |
| Pitofenone | ZINC000000538207 |
| Pituxate | ZINC000000000647, ZINC000002019903 |
| Pivagabine | ZINC000001545856 |
| Pivenfrine | ZINC000000000648, ZINC000005351265 |
| Pivopril | ZINC000004217336 |
| Pivoxazepam | ZINC000000608036, ZINC000001846318 |
| Pizotyline | ZINC000000001968 |
| Pki-166 | ZINC000000023255, ZINC000034032906 |
| Plafibride | ZINC000031297717 |
| Plazomicin | ZINC000068150640 |
| Pleconaril | ZINC000001537619 |
| Plinabulin | ZINC000003819466 |
| Podilfen | ZINC000026892432, ZINC000059299137 |
| Podofilox | ZINC000003861806 |
| Poldine | ZINC000000000651, ZINC000002034814 |
| Polydatin | ZINC000000899166, ZINC000003881959, ZINC000003881960, ZINC000003881961, ZINC000003881962, ZINC000004098633, ZINC000005923201, ZINC000005923204, ZINC000005923206, ZINC000005923208, ZINC000012888715, ZINC000012888720, ZINC000012888727, ZINC000012888734, ZINC000015919781, ZINC000021983181, ZINC000039205864, ZINC000065748638, ZINC000096032072, ZINC000096032073, ZINC000101954257, ZINC000106385665, ZINC000108555676, ZINC000142857948, ZINC000238785594, ZINC000253527835 |
| Polythiazide | ZINC000001530906, ZINC000001530907 |
| Pomaglumetad-methionil | ZINC000013983832 |
| Pomalidomide | ZINC000001997125, ZINC000003940470 |
| Pomisartan | ZINC000150340377 |
| Ponalrestat | ZINC000000538211 |
| Ponazuril | ZINC000033854754 |
| Posaconazole | ZINC000003938482, ZINC000011616816, ZINC000028639340, ZINC000060392777, ZINC000060392778, ZINC000060392779, ZINC000060392780, ZINC000072190231, ZINC000164119553, ZINC000220105129 |
| Posatirelin | ZINC000004217342 |
| Posizolid | ZINC000003982517 |
| Pozanicline | ZINC000000006562 |
| Practolol | ZINC000000000652, ZINC000000967917 |
| Pradefovir | ZINC000003939598, ZINC000011617052 |
| Pralidoxime | ZINC000004577910 |
| Pralnacasan | ZINC000003994903 |
| Pramipexole | ZINC000003781664, ZINC000003831352 |
| Pramiracetam | ZINC000001856108 |
| Pramiverine | ZINC000001482146 |
| Pramoxine | ZINC000019594594 |
| Prampine | ZINC000100369361, ZINC000100369364 |
| Pranazepide | ZINC000003798924 |
| Pranlukast | ZINC000001542146 |

Table S1. (continued next page)

| Drug name | ZINC ID |
| --- | --- |
| Pranolium | ZINC000000000653, ZINC000005003998 |
| Pranoprofen | ZINC000000000654, ZINC000003831354 |
| Pranosal | ZINC000001999275, ZINC000001999276, ZINC000001999277 |
| Prasterone_acetate | ZINC000001057676, ZINC000003881408, ZINC000004063548, ZINC000004113800, ZINC000004543799, ZINC000011996575, ZINC000039944338, ZINC000039944340, ZINC000043759612, ZINC000043759614, ZINC000078807626, ZINC000239164897, ZINC000253498200, ZINC000253498201, ZINC000253498202, ZINC000253500719, ZINC000253500722, ZINC000253608411, ZINC000253608420 |
| Prasugrel | ZINC000035653007, ZINC000035653009 |
| Pratosartan | ZINC000003786304 |
| Pravadoline | ZINC000030691168 |
| Pravastatin | ZINC000003798763 |
| Prazepam | ZINC000000001971 |
| Prazepine | ZINC000000001972 |
| Praziquantel | ZINC000000000655, ZINC000000403566 |
| Prazitone | ZINC000033854762, ZINC000033854764 |
| Prazosin | ZINC000095616601 |
| Prefenamate | ZINC000000001973 |
| Preladenant | ZINC000053006885 |
| Premazepam | ZINC000000001975 |
| Prenalterol | ZINC000000897007 |
| Prenoverine | ZINC000004214178, ZINC000033854766 |
| Prenoxdiazine | ZINC000000538227 |
| Prenylamine | ZINC000001532185, ZINC000001532186 |
| Presatovir | ZINC000147513579, ZINC000147519584, ZINC000205657764 |
| Pretamazium | ZINC000004214179 |
| Pretiadil | ZINC000004214180, ZINC000033854782 |
| Pretomanid | ZINC000000601826, ZINC000003821675 |
| Prezatide | ZINC000005605736, ZINC000013507674, ZINC000039817543, ZINC000097730338 |
| Pribecaine | ZINC000004214184 |
| Pridefine | ZINC000001482148 |
| Pridinol | ZINC000001482149 |
| Pridopidine | ZINC000022063703 |
| Prifelone | ZINC000000001977 |
| Prifinium | ZINC000001481906, ZINC000002037707 |
| Prifuroline | ZINC000000000657, ZINC000001846101 |
| Prilocaine | ZINC000001530816, ZINC000001530817 |
| Primaperone | ZINC000000001978 |
| Primaquine | ZINC000001530862, ZINC000001530863 |
| Primidolol | ZINC000001532362, ZINC000001532363 |
| Prinaberel | ZINC000003817763 |
| Prinomastat | ZINC000000580328 |
| Prinoxodan | ZINC000005700104 |
| Prizidilol | ZINC000000000658, ZINC000001842846 |
| Proadifen | ZINC000001671505 |
| Probenecid | ZINC000000001982 |
| Probicromil | ZINC000000001983 |
| Probucol | ZINC000001530755 |
| Procaterol | ZINC000019632678, ZINC000019632683, ZINC000019632688, ZINC000019632692 |
| Prochlorperazine | ZINC000019796018 |
| Procinolol | ZINC000000000659, ZINC000001567464 |

Table S1. (continued next page)

| Drug name | ZINC ID |
| --- | --- |
| Proclonol | ZINC000000001985 |
| Procromil | ZINC000000001986 |
| Procyclidine | ZINC000000004760, ZINC000000057455 |
| Prodilidine | ZINC000004626682, ZINC000005065030 |
| Prodipine | ZINC000001482151 |
| Prodolic-acid | ZINC000000000662, ZINC000002008408 |
| Profexalone | ZINC000002019921, ZINC000002019922 |
| Proflavine | ZINC000003775644 |
| Proflazepam | ZINC000000608045, ZINC000033854789 |
| Progabide | ZINC000013519914 |
| Proglumide | ZINC000002033840, ZINC000002033841 |
| Proheptazine | ZINC000004626683, ZINC000005459569 |
| Prolintane | ZINC000001481908, ZINC000001678599 |
| Promazine | ZINC000000010402 |
| Promethazine | ZINC000000020250, ZINC000000056647 |
| Promolate | ZINC000031297906 |
| Pronetalol | ZINC000000000666, ZINC000000967921 |
| Pronilide | ZINC000001846541 |
| Propacetamol | ZINC000055161176 |
| Propafenone | ZINC000001530759, ZINC000001530760 |
| Propamidine | ZINC000001665564 |
| Propanidid | ZINC000002039588 |
| Propantheline | ZINC000001530761 |
| Proparacaine | ZINC000001530762 |
| Propatyl-nitrate | ZINC000008214664 |
| Propazolamide | ZINC000004217364 |
| Propenzolate | ZINC000000000667, ZINC000004214202, ZINC000011682209, ZINC000011682210 |
| Properidine | ZINC000000001991 |
| Propetamide | ZINC000001846047, ZINC000001846050 |
| Propiomazine | ZINC000000000669, ZINC000000896849 |
| Propipocaine | ZINC000001999568 |
| Propiram | ZINC000000013347, ZINC000001850621 |
| Propisergide | ZINC000033858308, ZINC000059868006 |
| Propiverine | ZINC000001530934 |
| Propizepine | ZINC000000000671, ZINC000005141713 |
| Propoxate | ZINC000001664579, ZINC000013209330 |
| Propoxur | ZINC000001590885 |
| Propoxycaine | ZINC000001530942 |
| Propranolol | ZINC000000020240, ZINC000000056556 |
| Propyperone | ZINC000000538240 |
| Propyromazine | ZINC000001678354, ZINC000001999373 |
| Proquazone | ZINC000000001995 |
| Proquinolate | ZINC000000000672 |
| Proroxan | ZINC000000340372, ZINC000003087481 |
| Proscillaridin | ZINC000008143879, ZINC000008214665, ZINC000038139432, ZINC000056897526, ZINC000118915483, ZINC000118915484, ZINC000118917543, ZINC000118917544, ZINC000257355131, ZINC000257355132, ZINC000257355133, ZINC000257488916, ZINC000257488917, ZINC000257488918, ZINC000257488919, ZINC000306121901, ZINC000306121902, ZINC000306121903 |
| Prosulpride | ZINC000000608049, ZINC000001846137 |
| Proterguride | ZINC000003798387 |

Table S1. (continued next page)

| Drug name | ZINC ID |
| --- | --- |
| Protheobromine | ZINC000000000675, ZINC000013209481 |
| Prothipendyl | ZINC000000001996 |
| Prothixene | ZINC000000001997 |
| Protiofate | ZINC000001846265 |
| Protirelin | ZINC000004096261 |
| Protizinic-acid | ZINC000000000676, ZINC000001842999 |
| Protokylol | ZINC000000000677, ZINC000001704226, ZINC000001704227, ZINC000001704228 |
| Protriptyline | ZINC000001530764 |
| Proxazole | ZINC000000000678, ZINC000001567482 |
| Proxibutene | ZINC000003623586, ZINC000003623588 |
| Proxicromil | ZINC000000001999 |
| Proxyphylline | ZINC000000057347, ZINC000000057348 |
| Prozapine | ZINC000001482157 |
| Prucalopride | ZINC000001891034 |
| Prulifloxacin | ZINC000000596006, ZINC000001269201 |
| Psilocybine | ZINC000001530830 |
| Pumaprazole | ZINC000000006152 |
| Pumosetrag | ZINC000000016869 |
| Puromycin | ZINC000053147179 |
| Pyrantel | ZINC000000097996 |
| Pyrathiazine | ZINC000000002004 |
| Pyrazofurin | ZINC000004217381 |
| Pyricarbate | ZINC000000057462 |
| Pyrilamine | ZINC000019144216 |
| Pyrimethamine | ZINC000000057464 |
| Pyrinoline | ZINC000000538243 |
| Pyronaridine | ZINC000003882897 |
| Pyrophenindane | ZINC000031574997 |
| Pyrovalerone | ZINC000000000681, ZINC000001849892 |
| Pyroxamine | ZINC000004744324, ZINC000004744326 |
| Pyrrobutamine | ZINC000015848271 |
| Pyrrocaine | ZINC000000002011 |
| Pyrroliphene | ZINC000002037161, ZINC000002037162, ZINC000002037163, ZINC000004214232 |
| Pyrrolnitrin | ZINC000000002012 |
| Pyrvinium | ZINC000003831401 |
| Pytamine | ZINC000000000683, ZINC000033858310 |
| Qav680 | ZINC000034600373 |
| Quatacaine | ZINC000002019972 |
| Quazinone | ZINC000003874826 |
| Quazodine | ZINC000000002014 |
| Quazolast | ZINC000000002015 |
| Quetiapine | ZINC000019632628 |
| Quifenadine | ZINC000001298962, ZINC000001298963 |
| Quiflapon | ZINC000001914818 |
| Quilostigmine | ZINC000056897587 |
| Quinacainol | ZINC000000000684, ZINC000002021299 |
| Quinacrine | ZINC000018847040, ZINC000018847041 |
| Quinagolide | ZINC000031274831, ZINC000031274834 |
| Quinaldine-blue | ZINC000001687632 |
| Quinaprilat | ZINC000004217387 |

Table S1. (continued next page)

| Drug name | ZINC ID |
| --- | --- |
| Quinapril | ZINC000003801163 |
| Quinazosin | ZINC000000002016 |
| Quincarbate | ZINC000001866283, ZINC000005385675 |
| Quindonium | ZINC000000000685, ZINC000001718902, ZINC000001718903, ZINC000001718904 |
| Quinelorane | ZINC000000005041 |
| Quinethazone | ZINC000000000686, ZINC000000896838 |
| Quinezamide | ZINC000000002017 |
| Quinine-ethylcarbonate | ZINC000001024784, ZINC000002041226, ZINC000003873877, ZINC000003873878, ZINC000003873879, ZINC000003873880, ZINC000003873993, ZINC000004618164, ZINC000043721833, ZINC000195800671 |
| Quinine | ZINC000006484901 |
| Quinocide | ZINC000001682298, ZINC000001814403 |
| Quinotolast | ZINC000000002018 |
| Quinpirole | ZINC000004629192 |
| Quinterenol | ZINC000000000690, ZINC000001582760 |
| Quinuclium | ZINC000002020496 |
| Quinupramine | ZINC000004214242, ZINC000005162687 |
| Quipazine | ZINC000000002020 |
| Quisinostat | ZINC000035836133 |
| Quisultazine | ZINC000004214243, ZINC000005317815 |
| Quizartinib | ZINC000043204002 |
| R-1487 | ZINC000058633224 |
| R428 | ZINC000051951668, ZINC000051951669 |
| Rabacfosadine | ZINC000034893919 |
| Rabeprazole | ZINC000103593842 |
| Rabusertib | ZINC000070466463 |
| Racecadotril | ZINC000001546843, ZINC000001546844 |
| Racemethorphan | ZINC000000001614, ZINC000000003649 |
| Raclopride | ZINC000025757754 |
| Ractopamine | ZINC000000000694, ZINC000000001078, ZINC000002014184, ZINC000002014185 |
| Radafaxine | ZINC000005377133 |
| Radezolid | ZINC000040379938 |
| Radiprodil | ZINC000028363953 |
| Radotinib | ZINC000059749972 |
| Rafabegron | ZINC000003952725 |
| Ragaglitazar | ZINC000001481830 |
| Ralfinamide | ZINC000072190123 |
| Ralimetinib | ZINC000034630490 |
| Raloxifene | ZINC000000538275 |
| Raltegravir | ZINC000013831130 |
| Raltitrexed | ZINC000001625751, ZINC000003832372 |
| Raluridine | ZINC000001851238 |
| Ramatroban | ZINC000003798772 |
| Ramelteon | ZINC000000007031 |
| Ramifenazone | ZINC000000057367 |
| Ramiprilat | ZINC000004217399 |
| Ramipril | ZINC000003798757, ZINC000003831408, ZINC000003831409, ZINC000004245622, ZINC000005650658, ZINC000007997924, ZINC000009212658, ZINC000015973889, ZINC000033359806, ZINC000038342089, ZINC000059191484, ZINC000059870001, ZINC000146927171, ZINC000253951174 |
| Ramixotidine | ZINC000004217400 |

Table S1. (continued next page)

| Drug name | ZINC ID |
| --- | --- |
| Ramosetron | ZINC000003873275, ZINC000005116719 |
| Ranirestat | ZINC000000598588 |
| Ranolazine | ZINC000095619100, ZINC000095619101 |
| Rapastinel | ZINC000071773625, ZINC000196963536, ZINC000255974006, ZINC000255974007, ZINC000255974008 |
| Ravidasvir | ZINC000150607150 |
| Raxatrigine | ZINC000034892269, ZINC000113543588, ZINC000113543589, ZINC000113543591 |
| Raxofelast | ZINC000003784912, ZINC000005764976 |
| Razaxaban | ZINC000003633839 |
| Razinodil | ZINC000072266825, ZINC000072266826 |
| Razobazam | ZINC000000002024 |
| Razoxane | ZINC000087515509, ZINC000087515510 |
| Rebamipide | ZINC000009330879, ZINC000009330880 |
| Rebastinib | ZINC000063933734 |
| Reboxetine | ZINC000000002275, ZINC000000002284, ZINC000000006923, ZINC000003996032 |
| Recainam-tosylate | ZINC000001854708 |
| Reclazepam | ZINC000000538279 |
| Regadenoson | ZINC000003598383, ZINC000011617063, ZINC000013818943, ZINC000065748602, ZINC000090697750 |
| Reglitazar | ZINC000006069082, ZINC000084462508 |
| Regorafenib | ZINC000006745272 |
| Relcovaptan | ZINC000003931527 |
| Relebactam | ZINC000043206319 |
| Relenopride | ZINC000118666544 |
| Remacemide | ZINC000000004153, ZINC000000228297 |
| Remifentanil | ZINC000000538283 |
| Remikiren | ZINC000004217406 |
| Reminertant | ZINC000001482927 |
| Remogliflozin-etabonate | ZINC000003979756, ZINC000146654099 |
| Remoxipride | ZINC000002021799 |
| Repaglinide | ZINC000003798537, ZINC000003831411 |
| Reparixin | ZINC000000008717 |
| Repinotan | ZINC000001552489 |
| Repirinast | ZINC000000538285 |
| Reproterol | ZINC000001542931, ZINC000002019839 |
| Reserpine | ZINC000003938746, ZINC000003978051 |
| Resiquimod | ZINC000028572103 |
| Resorantel | ZINC000000002027 |
| Retelliptine | ZINC000001846081 |
| Retinol_acetate | ZINC000003874857, ZINC000005456980, ZINC000013516720, ZINC000013516722, ZINC000014685555, ZINC000026892410, ZINC000040454325, ZINC000040454328, ZINC000087492913, ZINC000888085225 |
| Retosiban | ZINC000006718496 |
| Revaprazan | ZINC000003633835, ZINC000003633836 |
| Revatropate | ZINC000004214265 |
| Revefenacin | ZINC000113648937 |
| Revenast | ZINC000000538286 |
| Revexepride | ZINC000003916772 |
| Revizinone | ZINC000000538290 |
| Revospirone | ZINC000056134943 |
| Rg-1530 | ZINC000035930738 |

Table S1. (continued next page)

| Drug name | ZINC ID |
| --- | --- |
| Rg-547 | ZINC000013983251 |
| Rg-7603 | ZINC000095587536, ZINC000117249555 |
| Rgb-286638 | ZINC000043128366 |
| Rhodoquine | ZINC000001683561 |
| Ribavirin | ZINC000000896749, ZINC000001035331, ZINC000001091444, ZINC000003831418, ZINC000003831419, ZINC000003831420, ZINC000003831421, ZINC000006603353, ZINC000011592714, ZINC000013473647, ZINC000021981353, ZINC000021981355, ZINC000025975767, ZINC000028463692, ZINC000040479339 |
| Ribociclib | ZINC000072316335 |
| Riboprine | ZINC000000057125 |
| Ribostamycin | ZINC000053255716 |
| Ricasetron | ZINC000100368206 |
| Ridazolol | ZINC000001999464, ZINC000001999465 |
| Ridogrel | ZINC000001536934 |
| Rilapladib | ZINC000003973276 |
| Rilmakalim | ZINC000003801510 |
| Rilmazafone | ZINC000000538301 |
| Rilmenidine | ZINC000000009708 |
| Rilopirox | ZINC000000538303 |
| Rilozarone | ZINC000084758639 |
| Riluzole | ZINC000000006481 |
| Rimacalib | ZINC000004423231 |
| Rimantadine | ZINC000003831429, ZINC000003831430 |
| Rimazolium | ZINC000000000699, ZINC000001842857 |
| Rimcazole | ZINC000022920326 |
| Rimegepant | ZINC000068267814 |
| Rimeporide | ZINC000003951217 |
| Rimiterol | ZINC000001565247 |
| Rimonabant | ZINC000001540228 |
| Riociguat | ZINC000003819392 |
| Ripasudil | ZINC000003940873, ZINC000005983647 |
| Ripazepam | ZINC000000002033 |
| Ripisartan | ZINC000000538309 |
| Risarestat | ZINC000005820738, ZINC000005820739 |
| Risotilide | ZINC000001552231 |
| Rispenzepine | ZINC000000004265, ZINC000003795847 |
| Risperidone | ZINC000000538312 |
| Ritanserin | ZINC000000538314 |
| Ritiometan | ZINC000002019920 |
| Ritobegron | ZINC000072266295 |
| Ritodrine | ZINC000000057480, ZINC000000057483 |
| Ritolukast | ZINC000000538316 |
| Ritonavir | ZINC000003944422, ZINC000011615852, ZINC000026658154, ZINC000027719492, ZINC000029416472, ZINC000040163372, ZINC000040163373, ZINC000040163374, ZINC000097975875, ZINC000150342340, ZINC000206511622 |
| Rivanicline | ZINC000001543478 |
| Rivaroxaban | ZINC000001494180, ZINC000003964126 |
| Rivastigmine | ZINC000000004413 |
| Riviciclib | ZINC000003937395, ZINC000066032179, ZINC000113496418, ZINC000113498699 |
| Rivoglitazone | ZINC000001481958, ZINC000004214702 |
| Rizatriptan | ZINC000000005895 |

Table S1. (continued next page)

| Drug name | ZINC ID |
| --- | --- |
| Ro-3201195 | ZINC000013683103, ZINC000013683106 |
| Robalzotan | ZINC000003811952 |
| Robenacoxib | ZINC000000007562 |
| Robenidine | ZINC000002013227 |
| Rociclovir | ZINC000001537487 |
| Rociverine | ZINC000001846348, ZINC000001846351 |
| Rocuronium | ZINC000053229445 |
| Rodocaine | ZINC000000002034 |
| Rofelodine | ZINC000031297912, ZINC000031297915 |
| Roflumilast | ZINC000000592419 |
| Rogletimide | ZINC000011535892, ZINC000013232857 |
| Rolafagrel | ZINC000043006881 |
| Rolapitant | ZINC000003816514 |
| Rolgamidine | ZINC000029390095 |
| Rolicyprine | ZINC000072266300 |
| Rolipram | ZINC000000004982, ZINC000002000919 |
| Rolodine | ZINC000000002036 |
| Rolofylline | ZINC000018207215, ZINC000100377625 |
| Rolziracetam | ZINC000000002037 |
| Romazarit | ZINC000049925478 |
| Romergoline | ZINC000003782563 |
| Romifenone | ZINC000033872441 |
| Romifidine | ZINC000056898821 |
| Ronacaleret | ZINC000030691809 |
| Ronactolol | ZINC000002019895, ZINC000002019896 |
| Ronidazole | ZINC000001482167 |
| Ronifibrate | ZINC000001846400 |
| Ropinirole | ZINC000000002041 |
| Ropitoin | ZINC000004214281, ZINC000011682492 |
| Ropivacaine | ZINC000000897002 |
| Ropizine | ZINC000031291823 |
| Roquinimex | ZINC000100001952 |
| Rosiglitazone | ZINC000000968328, ZINC000000968330 |
| Rosoxacin | ZINC000000002042 |
| Rosuvastatin | ZINC000001535101 |
| Rotigotine | ZINC000000004028, ZINC000000005151 |
| Rotoxamine | ZINC000000002043 |
| Roxadimate | ZINC000000000704, ZINC000002032871, ZINC000002032875 |
| Roxadustat | ZINC000071257465 |
| Roxatidine-acetate | ZINC000003812908 |
| Roxifiban | ZINC000003948981 |
| Roxindole | ZINC000001548439 |
| Roxoperone | ZINC000000002045 |
| Rubitecan | ZINC000003827362 |
| Rucaparib | ZINC000000025958 |
| Rufinamide | ZINC000000007782 |
| Rufloxacin | ZINC000000538328 |
| Rupatadine | ZINC000000598829 |
| Ruzadolane | ZINC000067839144 |
| Sabeluzole | ZINC000001999437, ZINC000004214288 |

| Drug name | ZINC ID |
| --- | --- |
| Sabiporide | ZINC000033999019 |
| Sacubitril | ZINC000003792417 |
| S-adenosyl-methionine | ZINC000004214738, ZINC000004228231, ZINC000012371977, ZINC000012371978, ZINC000012371979, ZINC000012371980, ZINC000013522357, ZINC000013522362, ZINC000033821030, ZINC000033821031, ZINC000071755544, ZINC000071755545, ZINC000071755557, ZINC000071755558, ZINC000071755565, ZINC000095644663, ZINC000095644664, ZINC000139339614, ZINC000254297245, ZINC000254297254, ZINC000254297257 |
| Safinamide | ZINC000053084692 |
| Safironil | ZINC000001541080 |
| Safotibant | ZINC000003928767 |
| Salafibrate | ZINC000004217430 |
| Salazodine | ZINC000003917759 |
| Salazosulfadimidine | ZINC000004194759 |
| Salazosulfamide | ZINC000003953923 |
| Salazosulfathiazole | ZINC000004194760 |
| Salclobuzic-acid | ZINC000019502565 |
| Salethamide | ZINC000004217432 |
| Salfluverine | ZINC000000002052 |
| Salicin | ZINC000003847505 |
| Salicylamide | ZINC000000002055 |
| Salicylanilide | ZINC000000002057 |
| Salinazid | ZINC000017289849 |
| Salmefamol | ZINC000000000705, ZINC000000967940, ZINC000005735278, ZINC000005735280 |
| Salmisteine | ZINC000001905201 |
| Salnacedin | ZINC000004217434 |
| Salprotoside | ZINC000004214292, ZINC000084759448, ZINC000084759449, ZINC000084759450 |
| Salsalate | ZINC000000002062 |
| Salverine | ZINC000000002064 |
| Samatasvir | ZINC000150588806 |
| Samixogrel | ZINC000001538600 |
| Sampirtine | ZINC000053277475 |
| Sanguinarium | ZINC000000000706 |
| Saperconazole | ZINC000003913989, ZINC000004214295, ZINC000026168046, ZINC000028712570, ZINC000028712571, ZINC000163773658 |
| Sapitinib | ZINC000034587071 |
| Saprisartan-potassium | ZINC000003919581 |
| Sapropterin | ZINC000013585233 |
| Saquinavir | ZINC000003914596 |
| Sar-407899 | ZINC000034603838 |
| Saracatinib | ZINC000024811973 |
| Sarafloxacin | ZINC000000538330 |
| Sarakalim | ZINC000000598479 |
| Sardomozide | ZINC000100023874 |
| Saredutant | ZINC000003927605 |
| Saripidem | ZINC000000004507 |
| Sarizotan | ZINC000000021067 |
| Sarmazenil | ZINC000000002065 |
| saroglitazar | ZINC000146274983 |
| Sarolaner | ZINC000103297729 |
| Sarpogrelate | ZINC000003798720, ZINC000003874934 |

Table S1. (continued next page)

| Drug name | ZINC ID |
| --- | --- |
| Satranidazole | ZINC000004217442 |
| Saviprazole | ZINC000001552562, ZINC000011687804 |
| Sb-649868 | ZINC000043207236 |
| Sb-705498 | ZINC000013985502, ZINC000034297799 |
| Sb-742457 | ZINC000043199965 |
| Sch-900776 | ZINC000060328032, ZINC000060328033 |
| Scopinast | ZINC000003944646 |
| Sd-0006 | ZINC000006718469 |
| Sdx-101 | ZINC000000003642, ZINC000000057313 |
| Seclazone | ZINC000000000710, ZINC000002011672 |
| Secnidazole | ZINC000000000711, ZINC000001843047 |
| Secobarbital | ZINC000002005550, ZINC000003874950 |
| Seganserin | ZINC000000538333 |
| Seletracetam | ZINC000011726772, ZINC000011726774 |
| Selexipag | ZINC000003990451 |
| Seliciclib | ZINC000001649340 |
| Selodenoson | ZINC000003989599 |
| Selprazine | ZINC000033872449 |
| Semagacestat | ZINC000003989041 |
| Sematilide | ZINC000004194761 |
| Sembragiline | ZINC000013975983 |
| Semorphone | ZINC000003793062 |
| Senazodan | ZINC000005161845 |
| Senicapoc | ZINC000003816408 |
| Seperidol | ZINC000000538335 |
| Sepimostat | ZINC000003778392 |
| Seproxetine | ZINC000000004531 |
| Sequifenadine | ZINC000001846272, ZINC000005782839 |
| Serazapine | ZINC000000596677, ZINC000003775351 |
| Serfibrate | ZINC000004214313, ZINC000038825822 |
| Sergliflozin_etabonate | ZINC000003974200, ZINC000114243644 |
| Seridopidine | ZINC000035838156 |
| Sermetacin | ZINC000000608072 |
| Sertaconazole | ZINC000000606383, ZINC000002016037 |
| Sertindole | ZINC000000538337 |
| Sertraline | ZINC000001853550 |
| Setastine | ZINC000000601258, ZINC000002001312 |
| Setazindol | ZINC000000014718, ZINC000001692728 |
| Setileuton | ZINC000036487556 |
| Setipafant | ZINC000001481922 |
| Setipiprant | ZINC000091291806 |
| Setiptiline | ZINC000001482169 |
| Setoperone | ZINC000000538339 |
| Sevopramide | ZINC000031570061, ZINC000031570064 |
| Sezolamide | ZINC000001536771 |
| Sgi-1776 | ZINC000068205235 |
| Sgx-523 | ZINC000039129916 |
| Sibopirdine | ZINC000000538342 |
| Sibrafiban | ZINC000011726787 |
| Sibutramine | ZINC000000004759, ZINC000000531272 |

Table S1. (continued next page)

| Drug name | ZINC ID |
| --- | --- |
| Sifaprazine | ZINC000000002068 |
| Siguazodan | ZINC000003995872, ZINC000003995874 |
| Sildenafil | ZINC000019796168 |
| Silmitasertib | ZINC000058638454 |
| Silodosin | ZINC000003806063, ZINC000003806069 |
| Siltenzepine | ZINC000000538351 |
| Simenepag | ZINC000084758357 |
| Simetride | ZINC000004217449 |
| Simfibrate | ZINC000003874963 |
| Simvastatin | ZINC000003780893 |
| Sinefungin | ZINC000004217451 |
| Sinitrodil | ZINC000003777871 |
| Sipatrigine | ZINC000000538354 |
| Siponimod | ZINC000006717453, ZINC000299848296 |
| Siramesine | ZINC000001533883 |
| Siratiazem | ZINC000003802090 |
| Sitafloxacin | ZINC000003795983 |
| Sitagliptin | ZINC000001489478 |
| Sitaxentan | ZINC000001481831 |
| Sitogluside | ZINC000049888788, ZINC000085532584, ZINC000085645655, ZINC000118913171, ZINC000118913172, ZINC000118922613, ZINC000118922614, ZINC000118930262, ZINC000118930263, ZINC000169313894, ZINC000245190527, ZINC000245190528, ZINC000248264078, ZINC000248264080, ZINC000248264082, ZINC000248264084, ZINC000253476255, ZINC000253534731, ZINC000253534732, ZINC000253534733, ZINC000253558544, ZINC000253558545, ZINC000253558546, ZINC000253609312, ZINC000253609313, ZINC000253609314, ZINC000253609315 |
| Sivelestat | ZINC000021298097 |
| Sivifene | ZINC000003914981 |
| Sns-314 | ZINC000040393428 |
| Sobetirome | ZINC000013475083 |
| Sobuzoxane | ZINC000022032147 |
| Sodelglitazar | ZINC000001553281 |
| Sofinicline | ZINC000028866069 |
| Sofosbuvir | ZINC000100074252 |
| Solabegron | ZINC000001547346 |
| Solcitinib | ZINC000118401631 |
| Solifenacin | ZINC000003936683 |
| Solpecainol | ZINC000000004753 |
| Solypertine | ZINC000004214326 |
| Somantadine | ZINC000004217454 |
| Soneclosan | ZINC000002027016 |
| Sonedenoson | ZINC000003966398 |
| Sonepiprazole | ZINC000003812548 |
| Sopitazine | ZINC000000538361 |
| Sopromidine | ZINC000003620813 |
| Sorafenib | ZINC000001493878 |
| Soraprazan | ZINC000003924220 |
| Sorbinil | ZINC000000002070 |
| Soretolide | ZINC000000002071 |
| Sorivudine | ZINC000003653374 |
| Sotalol | ZINC000000004166, ZINC000000896819 |

Table S1. (continued next page)

| Drug name | ZINC ID |
| --- | --- |
| Sotirimod | ZINC000030691235 |
| Sovaprevir | ZINC000085537149 |
| Spaglumic-acid | ZINC000002504638, ZINC000004534089, ZINC000004534090, ZINC000004534091 |
| Sparfloxacin | ZINC000000538362 |
| Sparfosate | ZINC000001563934, ZINC000001756826 |
| Sparsentan | ZINC000006716826 |
| Sparteine | ZINC000000156956 |
| Spectinomycin | ZINC000053006806 |
| Spiclomazine | ZINC000000538363 |
| Spiperone | ZINC000000643233 |
| Spiradoline | ZINC000002530707, ZINC000003875228, ZINC000003875229 |
| Spiramide | ZINC000001846593 |
| Spiraprilat | ZINC000004217460 |
| Spirapril | ZINC000004217459 |
| Spirendolol | ZINC000000000722, ZINC000001846186 |
| Spirilene | ZINC000000538370 |
| Spirofylline | ZINC000084758966 |
| Spiroglumide | ZINC000001554851 |
| Spirotriazine | ZINC000000002074 |
| Spiroxatrine | ZINC000053113560, ZINC000053113561 |
| Spiroxepin | ZINC000001846391, ZINC000001846392, ZINC000001846394, ZINC000004626693 |
| Spizofurone | ZINC000000002075 |
| St1535 | ZINC000001554291 |
| Stacofylline | ZINC000000597379 |
| Stavudine | ZINC000000137884 |
| Stepronin | ZINC000001846079, ZINC000001846080 |
| Stilbamidine | ZINC000000002079 |
| Stilbazium | ZINC000004217469 |
| Stilonium | ZINC000002036061 |
| Stirimazole | ZINC000004217471 |
| Stiripentol | ZINC000000000724, ZINC000002005958 |
| Stirocainide | ZINC000004214343 |
| Streptozotocin | ZINC000003831470, ZINC000003831471, ZINC000003977737, ZINC000003995968, ZINC000004245682, ZINC000013783417, ZINC000021985248, ZINC000033943585, ZINC000033943586, ZINC000044608463, ZINC000201026331, ZINC000253714309 |
| Strychnine | ZINC000000119434 |
| Subendazole | ZINC000005424997 |
| Succinobucol | ZINC000003937467 |
| Succinylsulfathiazole | ZINC000001532343 |
| Suclofenide | ZINC000001842729, ZINC000100368602 |
| Sufentanil | ZINC000000538386 |
| Sufotidine | ZINC000001843102 |
| Sulamserod | ZINC000026263147 |
| Sulazuril | ZINC000000538388 |
| Sulbenox | ZINC000004214350, ZINC000011726813 |
| Sulclamide | ZINC000000002088 |
| Sulfacecole | ZINC000004194762 |
| Sulfachrysoidine | ZINC000004217481 |
| Sulfaloxic-acid | ZINC000002020014 |
| Sulfamazone | ZINC000004194719, ZINC000005502399 |

Table S1. (continued next page)

| Drug name | ZINC ID |
| --- | --- |
| Sulfamethoxypyridazine-acetyl | ZINC000000196692 |
| sulfamidochrysoidine | ZINC000004097405, ZINC000103620160, ZINC000256164152 |
| Sulfanitran | ZINC000003873374 |
| Sulfasalazine | ZINC000003831490, ZINC000085550135, ZINC000100031653 |
| Sulfasuccinamide | ZINC000000125445 |
| Sulfinalol | ZINC000005735012, ZINC000005735014 |
| Sulfonterol | ZINC000000000729, ZINC000005353095 |
| Sulforidazine | ZINC000000608085, ZINC000002015244 |
| Sulfoxone | ZINC000018268128 |
| Sulicrinat | ZINC000000538394 |
| Sulindac | ZINC000003786192, ZINC000004475353, ZINC000012404515, ZINC000012503133, ZINC000013531944, ZINC000031356637 |
| Sulisatin | ZINC000004217484 |
| Sulisobenzone | ZINC000001690324 |
| Sulmarin | ZINC000001846413 |
| Sulmazole | ZINC000007997905, ZINC000017835656 |
| Sulmepride | ZINC000000000731, ZINC000004214355 |
| Sulocarbilate | ZINC000004217485 |
| Sulofenur | ZINC000000538395 |
| Sulosemide | ZINC000003612885 |
| Sulotroban | ZINC000005934092 |
| Suloxifen | ZINC000004217487 |
| Sulprosal | ZINC000004217488 |
| Sulprostone | ZINC000004474665 |
| Sulthiame | ZINC000000002119 |
| Sultopride | ZINC000000601298, ZINC000000967922 |
| Sulverapride | ZINC000000608086, ZINC000001846065 |
| Sumacetamol | ZINC000033650038 |
| Sumanirole | ZINC000003960633 |
| Sumarotene | ZINC000001541060 |
| Sumatriptan | ZINC000000014360 |
| Sumetizide | ZINC000004214359, ZINC000072266836 |
| Sunepitron | ZINC000003780500 |
| Supidimide | ZINC000000000732, ZINC000002005956 |
| Suplatast | ZINC000003935481, ZINC000005650754 |
| Suprofen | ZINC000000057503, ZINC000000057504 |
| Suricainide | ZINC000002021380 |
| Surinabant | ZINC000001549068 |
| Suronacrine | ZINC000000004267, ZINC000001851379 |
| Sutezolid | ZINC000003810825 |
| Suvorexant | ZINC000049036447 |
| Suxamethonium | ZINC000001530820 |
| Suxethonium | ZINC000004217493 |
| Suxibuzone | ZINC000003875039 |
| Symclosene | ZINC000003861288 |
| Syrosingopine | ZINC000004214363 |
| Tacedinaline | ZINC000000003803 |
| Taclamine | ZINC000001481926, ZINC000011616858 |
| Tacrolimus | ZINC000085537027, ZINC000169289411 |

Table S1. (continued next page)

| Drug name | ZINC ID |
| --- | --- |
| Tadalafil | ZINC000000538404, ZINC000003993855, ZINC000008204637, ZINC000008204642 |
| Tafamidis | ZINC000043206271 |
| Tafenoquine | ZINC000001888486, ZINC000009294040 |
| Tafluprost | ZINC000013912394, ZINC000043772559, ZINC000064858680, ZINC000098092131, ZINC000098092132, ZINC000098092133, ZINC000115749357, ZINC000115749359, ZINC000115749361, ZINC000165928027, ZINC000256341464, ZINC000256341468, ZINC000584578785 |
| Taglutimide | ZINC000017161193, ZINC000017161200 |
| Tak-285 | ZINC000064746555 |
| Tak-441 | ZINC000043207688 |
| Tak-593 | ZINC000095579919 |
| Tak-715 | ZINC000006717791 |
| Tak-901 | ZINC000035310420 |
| Tak-960 | ZINC000043203898 |
| Taladegib | ZINC000068247898 |
| Talarozole | ZINC000001488080, ZINC000013675264 |
| Talastine | ZINC000000002122 |
| Talazoparib | ZINC000072318110 |
| Talibegron | ZINC000001534825 |
| Talinolol | ZINC000000538582, ZINC000001846283 |
| Talipexole | ZINC000000006644 |
| Talmapimod | ZINC000034001955 |
| Talmetacin | ZINC000000608088, ZINC000001846459 |
| Talmetoprim | ZINC000000538405 |
| Talnetant | ZINC000001543566 |
| Talniflumate | ZINC000000601275, ZINC000001844627 |
| Talopram | ZINC000001686103, ZINC000001999541 |
| Talotrexin | ZINC000003807186 |
| Taloximine | ZINC000005689032 |
| Talsupram | ZINC000001999271, ZINC000001999272 |
| Taltirelin | ZINC000004217500 |
| Taltrimide | ZINC000000002124 |
| Tamatinib | ZINC000006745792 |
| Tameridone | ZINC000000538412 |
| Tametraline | ZINC000004217502 |
| Tamibarotene | ZINC000000538415 |
| Tamitinol | ZINC000038615568 |
| Tamolarizine | ZINC000022440925, ZINC000022942024 |
| Tamoxifen | ZINC000001530689 |
| Tampramine | ZINC000000538416 |
| Tamsulosin | ZINC000001530694 |
| Tandamine | ZINC000002020075, ZINC000004626695 |
| Tandospirone | ZINC000001545034 |
| Tandutinib | ZINC000003966243 |
| Taniplon | ZINC000000010613 |
| Tanogitran | ZINC000064527030 |
| Tanomastat | ZINC000000538656 |
| Tanzisertib | ZINC000100285204 |
| Tapentadol | ZINC000000020783 |
| Taprenepag | ZINC000072266311 |
| Taprizosin | ZINC000029042296 |

Table S1. (continued next page)

| Drug name | ZINC ID |
| --- | --- |
| Taprostene | ZINC000004217507 |
| Tarafenacin | ZINC000000593626, ZINC000003965738 |
| Tarazepide | ZINC000002015955 |
| Taribavirin | ZINC000003781686 |
| Taselisib | ZINC000068267049 |
| Tasidotin | ZINC000003952747 |
| Tasimelteon | ZINC000004392649 |
| Tasisulam | ZINC000003816311 |
| Tasosartan | ZINC000013444037 |
| Tasquinimod | ZINC000000602397 |
| Tasuldine | ZINC000000002126 |
| Taurolidine | ZINC000019322537 |
| Tavilermide | ZINC000136639443, ZINC000202208406 |
| Tazadolene | ZINC000001481927, ZINC000002017729 |
| Tazanolast | ZINC000001548562 |
| Tazasubrate | ZINC000000608089, ZINC000005863617 |
| Tazeprofen | ZINC000000000737, ZINC000072266911 |
| Tazifylline | ZINC000019801839, ZINC000019801842 |
| Taziprinone | ZINC000022463338 |
| Tazofelone | ZINC000000002274, ZINC000000005019 |
| Tazolol | ZINC000000000738, ZINC000001999252 |
| Tc-2216 | ZINC000003937363, ZINC000013975455 |
| Tebatizole | ZINC000000002127 |
| Tebuquine | ZINC000001585700 |
| Tecadenoson | ZINC000003985981 |
| Tecalcet | ZINC000001538900 |
| Tecarfarin | ZINC000013985547 |
| Tecastemizole | ZINC000000002303 |
| Tecovirimat | ZINC000035323125 |
| Tedalinab | ZINC000072266313 |
| Tedatioxetine | ZINC000034003654 |
| Tedisamil | ZINC000000003721 |
| Tedizolid-phosphate | ZINC000043100953, ZINC000117720221 |
| Tedizolid | ZINC000043100956 |
| Tefazoline | ZINC000000002128 |
| Tefenperate | ZINC000004214387 |
| Tefludazine | ZINC000022463345 |
| Teflutixol | ZINC000019367492, ZINC000023358283 |
| Tegafur | ZINC000000016956, ZINC000000119895 |
| Tegaserod | ZINC000001545565 |
| Tegobuvir | ZINC000100057121 |
| Telatinib | ZINC000000590964 |
| Telbivudine | ZINC000000002159, ZINC000000025672, ZINC000001842580, ZINC000002545102, ZINC000002572653, ZINC000003831529, ZINC000005765078, ZINC000006072455 |
| Telcagepant | ZINC000028827350 |
| Telenzepine | ZINC000019796161 |
| Telinavir | ZINC000003915259 |
| Telithromycin | ZINC000009574770 |
| Telmisartan | ZINC000001530886 |
| Telotristat | ZINC000084758235 |

Table S1. (continued next page)

| Drug name | ZINC ID |
| --- | --- |
| Temafloxacin | ZINC000002004103, ZINC000009133461 |
| Temanogrel | ZINC000035920376 |
| Temazepam | ZINC000000000740, ZINC000000896634 |
| Temelastine | ZINC000008214687 |
| Temocaprilat | ZINC000004099102 |
| Temocapril | ZINC000003808778 |
| Temozolomide | ZINC000001482184 |
| Temsavir | ZINC000034815611 |
| Tenatoprazole | ZINC000003791775, ZINC000009060429 |
| Teneligliptin | ZINC000036520254 |
| Teniloxazine | ZINC000004214395, ZINC000004626696 |
| Tenilsetam | ZINC000058438005, ZINC000058438006 |
| Teniposide | ZINC000003831506, ZINC000003831507, ZINC000003831508, ZINC000003831509, ZINC000003984009, ZINC000004099008, ZINC000004099009, ZINC000004166028, ZINC000028226553, ZINC000028226559, ZINC000028226562, ZINC000049605896, ZINC000049605897, ZINC000049605898, ZINC000067957213, ZINC000067957215, ZINC000077313306, ZINC000077313309, ZINC000095628281, ZINC000095628282, ZINC000150652465, ZINC000150652469, ZINC000150652472, ZINC000150674146, ZINC000201698152, ZINC000202773821, ZINC000202773837, ZINC000203773037, ZINC000204007294, ZINC000245190530, ZINC000245190531, ZINC000253928517, ZINC000253928518, ZINC000254077833 |
| Tenivastatin | ZINC000003833877 |
| Tenocyclidine | ZINC000000002131 |
| Tenofovir-disoproxil | ZINC000003929022, ZINC000011616672 |
| Tenofovir | ZINC000001543475, ZINC000002020246 |
| Tenonitrozole | ZINC000004217523 |
| Tenosal | ZINC000000012508 |
| Tenosiprol | ZINC000013896524 |
| Teopranitol | ZINC000004217526 |
| Teoprolol | ZINC000001846190, ZINC000001846193, ZINC000001846194, ZINC000001846195 |
| Tepirindole | ZINC000001846072 |
| Tepoxalin | ZINC000000607214 |
| Terameprocol | ZINC000016953163 |
| Terazosin | ZINC000095616599, ZINC000095616600 |
| Terbequinil | ZINC000001548093 |
| Terbucromil | ZINC000000002134 |
| Terbufibrol | ZINC000000000745, ZINC000031983330 |
| Terbuficin | ZINC000004217528 |
| Terbutaline | ZINC000000000746, ZINC000000002281 |
| Terconazole | ZINC000000897382, ZINC000003873934, ZINC000003873935, ZINC000003873936 |
| Terfenadine | ZINC000003812892, ZINC000003831511 |
| Terflavoxate | ZINC000000538430 |
| Terfluranol | ZINC000000002135 |
| Terguride | ZINC000003811327 |
| Terikalant | ZINC000003784096 |
| Terizidone | ZINC000000000747, ZINC000005785445, ZINC000030691445 |
| Terlakiren | ZINC000003927469 |
| Terodiline | ZINC000001481797, ZINC000001481798 |
| Tertatolol | ZINC000000000748, ZINC000005352943 |
| Terutroban | ZINC000003811419 |
| Tesaglitazar | ZINC000001550769 |

Table S1. (continued next page)

| Drug name | ZINC ID |
| --- | --- |
| Tesevatinib | ZINC000038912363, ZINC000101781468, ZINC000114456300, ZINC000117147301, ZINC000117147304 |
| Tesicam | ZINC000000000749, ZINC000002015796 |
| Tesmilifene | ZINC000000002139 |
| Tesofensine | ZINC000003953158 |
| Tetomilast | ZINC000000600360 |
| Tetracaine | ZINC000001530811 |
| Tetrachlorosalicylanilide | ZINC000001081469 |
| tetraethylphthalamide | ZINC000000001935 |
| Tetragastrin | ZINC000004217536, ZINC000004256786, ZINC000004256788, ZINC000058522901, ZINC000100057712 |
| Tetraxetan | ZINC000022059268 |
| Tetrazepam | ZINC000000002144 |
| Tetrazolast | ZINC000000004014 |
| Tetriprofen | ZINC000000000756, ZINC000006037100 |
| Tetroxoprim | ZINC000002020087 |
| Texacromil | ZINC000084757309, ZINC000084757311 |
| Tezacaftor | ZINC000068206930, ZINC000113568483 |
| Tezacitabine | ZINC000003777826 |
| Tezampanel | ZINC000005134714 |
| Tezosentan | ZINC000003954692 |
| Tg-02 | ZINC000068251500, ZINC000095938323 |
| Tg100-115 | ZINC000006718666 |
| Tg100-801 | ZINC000029136020 |
| Thalidomide | ZINC000001530947, ZINC000001530948 |
| Thebacon | ZINC000004217541 |
| Thenalidine | ZINC000000002146 |
| Thenium | ZINC000000002148 |
| Thenyldiamine | ZINC000000002149 |
| Theodrenaline | ZINC000001566349, ZINC000001843003 |
| Theofibrate | ZINC000000538438 |
| Thiabendazole | ZINC000000073711 |
| Thiamine | ZINC000000049153 |
| Thiamiprine | ZINC000001670996 |
| Thiazesim | ZINC000002015879, ZINC000004626698 |
| Thiazinamium | ZINC000000000761, ZINC000001705062 |
| Thiethylperazine | ZINC000022446674 |
| Thiocolchicoside | ZINC000004214436, ZINC000004245665 |
| Thioctic-acid-amide | ZINC000001532582, ZINC000001532583 |
| Thiohexamide | ZINC000000002157 |
| Thiopropazate | ZINC000022446680 |
| Thioproperazine | ZINC000022446685 |
| Thioridazine | ZINC000001530695, ZINC000001530697 |
| Thiothixene | ZINC000030690433 |
| Thiphenamil | ZINC000001957899 |
| Thonzylamine | ZINC000000057513 |
| Thozalinone | ZINC000000000764, ZINC000002031526 |
| Thurfyl-nicotinate | ZINC000000000765, ZINC000002041299 |
| Thymocartin | ZINC000004217550 |
| Thymotrinan | ZINC000004217552 |

Table S1. (continued next page)

| Drug name | ZINC ID |
| --- | --- |
| Tiamenidine | ZINC000000002646 |
| Tiamulin | ZINC000031983173 |
| Tianafac | ZINC000000002161 |
| Tiapirinol | ZINC000000000766, ZINC000004214443, ZINC000072266790, ZINC000072266792 |
| Tiapride | ZINC000001542927 |
| Tiaprofenic-acid | ZINC000000000767, ZINC000000002282 |
| Tiaprost | ZINC000004214444, ZINC000033955230 |
| Tiaramide | ZINC000027849754 |
| Tiazofurin | ZINC000004217560 |
| Tiazuril | ZINC000000538453 |
| Tibalosin | ZINC000001846215, ZINC000001846218, ZINC000006021137, ZINC000006036932 |
| Tibenelast | ZINC000000004417 |
| Tibenzate | ZINC000000002162 |
| Tibezonium | ZINC000111364754 |
| Tibric-acid | ZINC000000002163, ZINC000003307207, ZINC000003307209 |
| Tibrofan | ZINC000000538456 |
| Ticagrelor | ZINC000028957444, ZINC000077319512, ZINC000095626794, ZINC000143240186, ZINC000143240396, ZINC000143240622, ZINC000150295519, ZINC000150295908, ZINC000204935758, ZINC000204935778, ZINC000204935796, ZINC000214488649, ZINC000306122927, ZINC000306122928, ZINC000584578805, ZINC000605696436, ZINC000605696437, ZINC000605696438 |
| Ticalopride | ZINC000000404160 |
| Ticlopidine | ZINC000019594599 |
| Ticrynafen | ZINC000000002166 |
| Tideglusib | ZINC000013985228 |
| Tidembersat | ZINC000003823817 |
| Tiemonium | ZINC000000000769, ZINC000001842660 |
| Tienocarbine | ZINC000000000770 |
| Tienopramine | ZINC000000002167 |
| Tienoxolol | ZINC000002019973, ZINC000003778847 |
| Tiflamizole | ZINC000000538458 |
| Tiflorex | ZINC000000000772, ZINC000005853378 |
| Tifluadom | ZINC000000967961, ZINC000001532369 |
| Tiflucarbine | ZINC000000000773 |
| Tifurac | ZINC000000002168 |
| Tigemonam | ZINC000004217565, ZINC000261505098 |
| Tilidine | ZINC000000001268, ZINC000002014721, ZINC000002014722, ZINC000002014723 |
| Tilisolol | ZINC000000004698, ZINC000002019998 |
| Tilmacoxib | ZINC000000006662 |
| Tilnoprofen-arbamel | ZINC000001537480, ZINC000005161047 |
| Tilomisole | ZINC000000002172 |
| Tilorone | ZINC000000538461 |
| Tilozepine | ZINC000031490523 |
| Timcodar | ZINC000008214468 |
| Timefurone | ZINC000000002174 |
| Timelotem | ZINC000000000775, ZINC000004214455 |
| Timirdine | ZINC000003775160 |
| Timofibrate | ZINC000000000777, ZINC000001846208 |
| Timolol | ZINC000000002176 |
| Timoprazole | ZINC000000000778, ZINC000004626699 |
| Tinabinol | ZINC000004214460, ZINC000005513241, ZINC000005513401, ZINC000005513402 |

Table S1. (continued next page)

| Drug name | ZINC ID |
| --- | --- |
| Tinazoline | ZINC000000002177 |
| Tinidazole | ZINC000000113446 |
| Tinoridine | ZINC000019313075 |
| Tioclomarol | ZINC000004214466, ZINC000030691425, ZINC000030691430, ZINC000030691434 |
| Tioconazole | ZINC000000608101, ZINC000000897385 |
| Tiodazosin | ZINC000001846466 |
| Tiomergine | ZINC000004217573 |
| Tioperidone | ZINC000004217575 |
| Tiopinac | ZINC000000002179 |
| Tiopropamine | ZINC000004217577 |
| Tiospirone | ZINC000001915508 |
| Tioxacin | ZINC000000002180 |
| Tioxamast | ZINC000001846054 |
| Tioxaprofen | ZINC000001842648, ZINC000001842649 |
| Tioxidazole | ZINC000001842639 |
| Tioxolone | ZINC000000002181 |
| Tipelukast | ZINC000003796820 |
| Tipentosin | ZINC000002014159, ZINC000002014160 |
| Tipifarnib | ZINC000028473960 |
| Tipindole | ZINC000000190266 |
| Tipiracil | ZINC000100032379 |
| Tiplasinin | ZINC000003938676 |
| Tiprenolol | ZINC000000000781, ZINC000002007408 |
| Tiprinast | ZINC000000002182 |
| Tiprolisant | ZINC000034045468 |
| Tiprotimod | ZINC000002016020 |
| Tiracizine | ZINC000000538471 |
| Tirapazamine | ZINC000001607808 |
| Tirasemtiv | ZINC000095627912 |
| Tirofiban | ZINC000003806104 |
| Tiropramide | ZINC000003812925, ZINC000013907153 |
| Tisocromide | ZINC000002019916, ZINC000002019917, ZINC000002019918, ZINC000002019919 |
| Tivanidazole | ZINC000004217582 |
| Tivantinib | ZINC000100016063 |
| Tiviciclovir | ZINC000003600430 |
| Tivozanib | ZINC000001489430 |
| Tixadil | ZINC000006021047, ZINC000006036855 |
| Tixanox | ZINC000004214483, ZINC000005764912 |
| Tizanidine | ZINC000019702309 |
| Tizolemide | ZINC000000000785, ZINC000001842644 |
| Toborinone | ZINC000002015953, ZINC000003803111 |
| Tobuterol | ZINC000004214489, ZINC000031495045 |
| Tocainide | ZINC000000000786, ZINC000000002283 |
| Tocamphyl | ZINC000011616427, ZINC000011616429 |
| Tocladesine | ZINC000004214493 |
| Tofenacin | ZINC000001704297, ZINC000002018845 |
| Tofetridine | ZINC000003650805, ZINC000004626701, ZINC000031538739, ZINC000031538745 |
| Tofisoline | ZINC000031298137 |
| Tofogliflozin | ZINC000035826342 |
| Tolafentrine | ZINC000001493454 |

Table S1. (continued next page)

| Drug name | ZINC ID |
| --- | --- |
| Tolamolol | ZINC000002007883, ZINC000002007885 |
| Tolazamide | ZINC000000057512 |
| Tolazoline | ZINC000000125006 |
| Tolbutamide | ZINC000001530703 |
| Tolfamide | ZINC000008214694 |
| Tolfenamic-acid | ZINC000000002188 |
| Tolgabide | ZINC000004217584 |
| Tolimidone | ZINC000017120697 |
| Toliprolol | ZINC000002033145, ZINC000003644104 |
| Tolmesoxide | ZINC000000000791, ZINC000004611429 |
| Tolmetin | ZINC000000002191 |
| Tolnapersine | ZINC000000000792, ZINC000001846110 |
| Tolnidamine | ZINC000000002192 |
| Tolonidine | ZINC000000002193 |
| Tolonium | ZINC000004217587 |
| Toloxatone | ZINC000002011566, ZINC000005782275 |
| Tolpadol | ZINC000001999505, ZINC000031297954, ZINC000031297957 |
| Tolpentamide | ZINC000000002194 |
| Tolperisone | ZINC000000057519, ZINC000000057520 |
| Tolpiprazole | ZINC000000002195 |
| Tolpronine | ZINC000000000794, ZINC000003637819 |
| Tolpropamine | ZINC000001481933, ZINC000001842662 |
| Tolpyrramide | ZINC000000002196 |
| Tolquinzole | ZINC000000000795, ZINC000033903721 |
| Tolterodine | ZINC000000968336 |
| Toltrazuril | ZINC000000538476 |
| Tolufazepam | ZINC000000538478 |
| Tolvaptan | ZINC000001490477 |
| Tolycaine | ZINC000000002197 |
| Tomeglovir | ZINC000003923823 |
| Tomelukast | ZINC000003873163 |
| Tomoglumide | ZINC000005512692, ZINC000005512694 |
| Tomoxiprole | ZINC000000000796 |
| Tonabersat | ZINC000003823813 |
| Tonapofylline | ZINC000000603777 |
| Topilutamide | ZINC000034035805, ZINC000034035806 |
| Topiramate | ZINC000001543366, ZINC000003831559, ZINC000013612334, ZINC000013783493, ZINC000021984495, ZINC000023586802, ZINC000043574047, ZINC000056863626, ZINC000095616603, ZINC000096085732 |
| Topixantrone | ZINC000001538987 |
| Topotecan | ZINC000001611274 |
| Toprilidine | ZINC000001846333 |
| Toquizine | ZINC000000608107, ZINC000002029587, ZINC000005317406 |
| Torbafylline | ZINC000002016021 |
| Torcitabine | ZINC000000004253 |
| Toreforant | ZINC000043175812 |
| Torsemide | ZINC000000005823 |
| Tosedostat | ZINC000013914293 |
| Tosifen | ZINC000000002199 |
| Tosufloxacin | ZINC000021983587, ZINC000021983589 |

Table S1. (continued next page)

| Drug name | ZINC ID |
| --- | --- |
| Tosulur | ZINC000003604332 |
| Tozadenant | ZINC000013986943 |
| Tozasertib | ZINC000003820040 |
| Trabodenoson | ZINC000035931662, ZINC000214055339, ZINC000214055453, ZINC000642805870, ZINC000642805871 |
| Tracazolate | ZINC000026568953 |
| Tradipitant | ZINC000043194077 |
| Tramadol | ZINC000000000853, ZINC000000002200, ZINC000001849532, ZINC000002015652 |
| Tramazoline | ZINC000001534528 |
| Tramiprosate | ZINC000001529636 |
| Trandolapril | ZINC000001530704, ZINC000001853202, ZINC000001853205, ZINC000001853210, ZINC000003814193, ZINC000003831562, ZINC000003831563, ZINC000003831564, ZINC000003831565, ZINC000013298449, ZINC000013298452, ZINC000013298454, ZINC000035335172, ZINC000230591566 |
| Tranexamic_acid | ZINC000001542907, ZINC000100007011, ZINC000100071256 |
| Transcainide | ZINC000005782463, ZINC000095564686, ZINC000095564687 |
| Trantelinium | ZINC000100368273 |
| Trapencaine | ZINC000004217596, ZINC000005513064, ZINC000005513072, ZINC000039297512 |
| Traxanox | ZINC000000002203 |
| Traxoprodil | ZINC000000005936 |
| Trazitiline | ZINC000022463350 |
| Trazium | ZINC000005935149, ZINC000005935151 |
| Trazodone | ZINC000000538483 |
| Trazolopride | ZINC000033940852 |
| Trecadrine | ZINC000005751639 |
| Trefentanil | ZINC000000538484 |
| Trelanserin | ZINC000000602552 |
| Trelnarizine | ZINC000033954104 |
| Treloxinate | ZINC000000002204 |
| Trenizine | ZINC000022463111, ZINC000072266906 |
| Trepibutone | ZINC000000002208 |
| Trepirium | ZINC000001999360, ZINC000001999361 |
| Treprostinil | ZINC000003800475 |
| Treptilamine | ZINC000004214521 |
| Triacetyldiphenolisatin | ZINC000004217281 |
| Triafungin | ZINC000000002210 |
| Triamterene | ZINC000000120286 |
| Tribendilol | ZINC000033940853, ZINC000033940854 |
| Tribenoside | ZINC000003956884, ZINC000004654789 |
| Tribromsalan | ZINC000000538492 |
| Tributyl-citrate | ZINC000003875494 |
| Tricetamide | ZINC000001847507 |
| Triciribine-phosphate | ZINC000003916663, ZINC000005385243, ZINC000005385244, ZINC000005385245, ZINC000017104328, ZINC000026489083, ZINC000031750529, ZINC000044608145 |
| Triclabendazole | ZINC000001444556 |
| Triclazate | ZINC000000000806, ZINC000001692983 |
| Triclocarban | ZINC000000121480 |
| Triclosan | ZINC000000002216 |
| Tricyclamol | ZINC000001481939, ZINC000001842627 |
| Tridihexethyl | ZINC000000967932, ZINC000000967933 |
| Trifarotene | ZINC000113700519 |

Table S1. (continued next page)

| Drug name | ZINC ID |
| --- | --- |
| Trifenagrel | ZINC000000538498 |
| Trifezolac | ZINC000000538502 |
| Triflocin | ZINC000030691313 |
| Triflubazam | ZINC000000002218 |
| Triflumidate | ZINC000002014836 |
| Trifluomeprazine | ZINC000001579054, ZINC000004214555 |
| Trifluoperazine | ZINC000019418959 |
| Trifluperidol | ZINC000000538505 |
| Triflupromazine | ZINC000000538507 |
| Trifluridine | ZINC000000002219, ZINC000000056707, ZINC000003842753, ZINC000003984032, ZINC000004245721, ZINC000004529568, ZINC000008698272, ZINC000009214438 |
| Triflusal | ZINC000000002220 |
| Trihexyphenidyl | ZINC000000968273, ZINC000000968274 |
| Trimazosin | ZINC000000601252 |
| Trimebutine | ZINC000000608117, ZINC000003831578 |
| Trimecaine | ZINC000000042136 |
| Trimeperidine | ZINC000000000807, ZINC000002041327, ZINC000005768358, ZINC000005768424 |
| Trimetazidine | ZINC000019358638 |
| Trimethadione | ZINC000001530710 |
| Trimethamide | ZINC000000002225 |
| Trimethaphan | ZINC000003831581, ZINC000003831583 |
| Trimethidinium | ZINC000000000810, ZINC000004611018 |
| Trimethobenzamide | ZINC000000538509 |
| Trimethoprim | ZINC000006627681 |
| Trimethyldiphenylpropylamine | ZINC000002015731, ZINC000002015732 |
| Trimetozine | ZINC000000000811 |
| Trimetrexate | ZINC000000598852 |
| Trimexiline | ZINC000004214560, ZINC000033827808 |
| Trimipramine | ZINC000000968275, ZINC000003831586 |
| Trimoxamine | ZINC000000000813, ZINC000002029624 |
| Tripamide | ZINC000001481940 |
| Triparanol | ZINC000001692389, ZINC000003875502 |
| Tripelennamine | ZINC000019117728 |
| Triprolidine | ZINC000012503099 |
| Trixolane | ZINC000033852239, ZINC000033852240, ZINC000033852241, ZINC000033852242 |
| Trizoxime | ZINC000100367373 |
| Trocimine | ZINC000000000815 |
| Troglitazone | ZINC000000968276, ZINC000000968277, ZINC000000968278, ZINC000000968279 |
| Trolnitrate | ZINC000032709512 |
| Tromantadine | ZINC000004214578 |
| Tropabazate | ZINC000100369772 |
| Tropanserin | ZINC000100086928 |
| Tropapride | ZINC000100370403 |
| Tropenziline | ZINC000004217609 |
| Tropesin | ZINC000000638627, ZINC000000638630 |
| Tropicamide | ZINC000000057532, ZINC000000057533 |
| Tropisetron | ZINC000100019233 |
| Tropodifene | ZINC000100376956, ZINC000100376960 |
| Troquidazole | ZINC000005422165 |

Table S1. (continued next page)

| Drug name | ZINC ID |
| --- | --- |
| Trospectomycin | ZINC000004214587 |
| Trovafloxacin | ZINC000100030989 |
| Troxacitabine | ZINC000001642845 |
| Troxipide | ZINC000000000817, ZINC000003812903 |
| Troxolamide | ZINC000002016046, ZINC000031291756 |
| Troxonium | ZINC000001626909 |
| Troxypyrrolium | ZINC000000000818 |
| Tubulozole | ZINC000003873395, ZINC000003873396, ZINC000003873398, ZINC000003873399 |
| Tuclazepam | ZINC000000000820, ZINC000004214594 |
| Tulobuterol | ZINC000000020236, ZINC000000057521 |
| Tulopafant | ZINC000002016001 |
| Tuvatidine | ZINC000004217611 |
| Tymazoline | ZINC000000002232 |
| Ubenimex | ZINC000001542895 |
| Ubisindine | ZINC000000000822, ZINC000004214601 |
| Ubrogepant | ZINC000095598454 |
| Udenafil | ZINC000004824235, ZINC000013916432 |
| Ufenamate | ZINC000001542912 |
| Ufiprazole | ZINC000013603907 |
| Uk432097 | ZINC000095539256, ZINC000150664074 |
| Ulifloxacin | ZINC000003791737, ZINC000004655141 |
| Ulodesine | ZINC000005973821 |
| Umeclidinium | ZINC000034608502 |
| Umespirone | ZINC000003781315 |
| Upadacitinib | ZINC000147178941, ZINC000147180128 |
| Upidosin | ZINC000003796141 |
| Uprifosbuvir | ZINC000149133266, ZINC000504242574, ZINC000504242575, ZINC000504242576, ZINC000504242577, ZINC000725382217 |
| Uprosertib | ZINC000043197676 |
| Urapidil | ZINC000001544805 |
| Uridine-triacetate | ZINC000000598966, ZINC000000643247, ZINC000001319228, ZINC000003843198, ZINC000004011869, ZINC000004011870, ZINC000004011871, ZINC000014825926, ZINC000019924448, ZINC000033951772, ZINC000033951773, ZINC000095829741 |
| Utibaprilat | ZINC000003798525 |
| Utibapril | ZINC000003782895 |
| Vabicaserin | ZINC000030691381 |
| Vadadustat | ZINC000117532869 |
| Vadocaine | ZINC000000000823, ZINC000001846083 |
| Valacyclovir | ZINC000001530713 |
| Valategrast | ZINC000072190226 |
| Valdecoxib | ZINC000000006694 |
| Valethamate | ZINC000002041203, ZINC000002041204, ZINC000002041205, ZINC000002041206 |
| Valganciclovir | ZINC000001543916, ZINC000001995484 |
| Valnemulin | ZINC000030727788 |
| Valproic_acid | ZINC000003008621 |
| Valpromide | ZINC000000002238 |
| Valsartan | ZINC000003831602, ZINC000003875259 |
| Valtorcitabine | ZINC000056898743 |
| Vamicamide | ZINC000000005295, ZINC000013736666, ZINC000013736669, ZINC000013736672 |
| Vandetanib | ZINC000053683345 |
| Vaneprim | ZINC000003604326, ZINC000003604328 |

Table S1. (continued next page)

| Drug name | ZINC ID |
| --- | --- |
| Vanoxerine | ZINC000022034135 |
| Vanyldisulfamide | ZINC000003604324 |
| Vapiprost | ZINC000013719916 |
| Vapitadine | ZINC000000008768 |
| Vardenafil | ZINC000018324776 |
| Varenicline | ZINC000001481833 |
| Varespladib-methyl | ZINC000001550156 |
| Varespladib | ZINC000001543773 |
| Varlitinib | ZINC000013980035 |
| Vatalanib | ZINC000000007460 |
| Vebufloxacin | ZINC000001999493, ZINC000005320340 |
| Vedaclidine | ZINC000001851108, ZINC000001851109 |
| Vedaprofen | ZINC000000000828, ZINC000001842638 |
| Veliflapon | ZINC000000598193 |
| Veliparib | ZINC000084610155 |
| Velnacrine | ZINC000000003711, ZINC000000087000 |
| Velpatasvir | ZINC000203686879, ZINC000220902773, ZINC000504665931, ZINC000504665932, ZINC000504665933 |
| Vemurafenib | ZINC000052509366 |
| Venetoclax | ZINC000150338755 |
| Venlafaxine | ZINC000000006016, ZINC000000896698 |
| Venritidine | ZINC000100369525, ZINC000100369529 |
| Veralipride | ZINC000000601278, ZINC000000640548 |
| Verazide | ZINC000000061565 |
| Verecimon | ZINC000038562120 |
| Verlukast | ZINC000003784127, ZINC000003784397, ZINC000011616769, ZINC000011616770 |
| Vernakalant | ZINC000022010910 |
| Verofylline | ZINC000000000831, ZINC000011726209 |
| Verosudil | ZINC000115224602, ZINC000115224604 |
| Verubecestat | ZINC000144542146 |
| Verubulin | ZINC000035978229 |
| Verucerfont | ZINC000034661169 |
| Vesnarinone | ZINC000003781942 |
| Vestipitant | ZINC000003936035 |
| Vetrabutine | ZINC000000000832, ZINC000005965114 |
| Vicriviroc | ZINC000022010579 |
| Vidarabine-phosphoric-acid | ZINC000013543718, ZINC000504744046, ZINC000504744047, ZINC000504744048 |
| Vidarabine | ZINC000000895113, ZINC000000896706, ZINC000000970363, ZINC000002047403, ZINC000002047673, ZINC000002169830, ZINC000002572650, ZINC000003201876, ZINC000003201878, ZINC000003830178, ZINC000003830179, ZINC000003978047, ZINC000003978049, ZINC000004048240, ZINC000008580514 |
| Vidupiprant | ZINC000043206238 |
| Vigabatrin | ZINC000000403618, ZINC000003798750 |
| Viloxazine | ZINC000000000833, ZINC000001999403 |
| Viminol | ZINC000002019964, ZINC000005599001, ZINC000005599258, ZINC000005599259 |
| Vinblastine | ZINC000085432544 |
| Vinburnine | ZINC000019796061 |
| Vincamine | ZINC000001069082 |
| Vincanol | ZINC000004214628 |
| Vincantril | ZINC000031297735, ZINC000031297738 |

Table S1. (continued next page)

| Drug name | ZINC ID |
| --- | --- |
| Vindeburnol | ZINC000000000838, ZINC000001846122, ZINC000006117846, ZINC000036457832 |
| Vindesine | ZINC000008214470 |
| Vinflunine | ZINC000085537078 |
| Vinmegallate | ZINC000072266239 |
| Vinpoline | ZINC000004214635, ZINC000072266867 |
| Vintoperol | ZINC000004214637 |
| Vipadenant | ZINC000040863182 |
| Viquidil | ZINC000001583681, ZINC000001583682, ZINC000001583683, ZINC000004626710 |
| Viroxime | ZINC000017130448, ZINC000100088129 |
| Vismodegib | ZINC000040899447 |
| Visnadine | ZINC000014883340, ZINC000014883342 |
| Vofopitant | ZINC000022445948 |
| Voglibose | ZINC000003788703 |
| Volasertib | ZINC000100071772 |
| Volinanserin | ZINC000000598040 |
| Volixibat | ZINC000164888446 |
| Vonoprazan | ZINC000034842823 |
| Vorapaxar | ZINC000003925861 |
| Voriconazole | ZINC000000000842, ZINC000000014864, ZINC000011592754, ZINC000011592755 |
| Vorozole | ZINC000003786387, ZINC000003791538 |
| Vortioxetine | ZINC000034051848 |
| Vosaroxin | ZINC000026186622 |
| Voxergolide | ZINC000031544269 |
| Voxtalisib | ZINC000035308805 |
| Vs-5584 | ZINC000049090010 |
| Vx-702 | ZINC000036377992 |
| Vx-745 | ZINC000013493055 |
| Xaliproden | ZINC000000577115 |
| Xanoxic-acid | ZINC000004217622 |
| Xanthinol | ZINC000000000843, ZINC000006507052 |
| Xanthiol | ZINC000019366479, ZINC000019368884 |
| Xantocillin | ZINC000060184509 |
| Xenalipin | ZINC000000439311 |
| Xenazoic-acid | ZINC000000608131, ZINC000002044292 |
| Xenbucin | ZINC000000000111, ZINC000002015169 |
| Xenthiorate | ZINC000004214649, ZINC000033956457 |
| Xibenolol | ZINC000000000844, ZINC000001871526 |
| Xibornol | ZINC000031333982 |
| Xilobam | ZINC000012409258 |
| Ximelagatran | ZINC000012504524 |
| Ximoprofen | ZINC000000000845, ZINC000004214656, ZINC000005751713, ZINC000038615303 |
| Xinidamine | ZINC000000002246 |
| Xipamide | ZINC000000538538 |
| Xipranolol | ZINC000002019968, ZINC000002019969 |
| Xl-019 | ZINC000095560529, ZINC000095930152 |
| Xl-228 | ZINC000203005669 |
| Xorphanol | ZINC000004217628 |
| Xylamidine | ZINC000000000846, ZINC000005782247 |
| Xylazine | ZINC000000002248 |
| Xylocoumarol | ZINC000005765149 |

Table S1. (continued next page)

| Drug name | ZINC ID |
| --- | --- |
| Xylometazoline | ZINC000000057534 |
| Xyloxemine | ZINC000002003703 |
| Y-39983 | ZINC000013907839, ZINC000098091948 |
| Yohimbic-acid | ZINC000000000847, ZINC000003881708, ZINC000003947438, ZINC000003947439, ZINC000003947440, ZINC000005223909, ZINC000005776998, ZINC000009214629, ZINC000009302199, ZINC000072186823, ZINC000247338204, ZINC000247338215 |
| Yohimbine | ZINC000003860825 |
| Z160 | ZINC000020509316 |
| Zabiciprilat | ZINC000003793034 |
| Zabicipril | ZINC000003793032 |
| Zacopride | ZINC000000000848, ZINC000000003961 |
| Zafirlukast | ZINC000000896717 |
| Zalcitabine | ZINC000000039906, ZINC000000039908, ZINC000017175067, ZINC000017175068 |
| Zaldaride | ZINC000002016009, ZINC000013813280 |
| Zaltoprofen | ZINC000000016678, ZINC000003875311 |
| Zanapezil | ZINC000000598255 |
| Zankiren | ZINC000027084753 |
| Zapizolam | ZINC000000002251 |
| Zaprinast | ZINC000014953365 |
| Zardaverine | ZINC000009230249 |
| Zatosetron | ZINC000100093179 |
| Zd-4190 | ZINC000001490292 |
| Zenarestat | ZINC000000596737 |
| Zepastine | ZINC000100380177, ZINC000100380183 |
| Zeranol | ZINC000003831615 |
| Zetidoline | ZINC000000002252 |
| Zibotentan | ZINC000001491485 |
| Zicronapine | ZINC000022938019 |
| Zidapamide | ZINC000000608139, ZINC000004214668 |
| Zileuton | ZINC000000000850, ZINC000000896731 |
| Zilpaterol | ZINC000000004010, ZINC000003785294, ZINC000043163828, ZINC000043163830 |
| Zimeldine | ZINC000008099545 |
| Zimidoben | ZINC000000002255 |
| Zindotrine | ZINC000000002256 |
| Zindoxifene | ZINC000000538547 |
| Zinoconazole | ZINC000004217637 |
| Zipeprol | ZINC000030691529, ZINC000030691540 |
| Ziprasidone | ZINC000000538550 |
| Zofenopril | ZINC000003775162 |
| Zoficonazole | ZINC000004214672, ZINC000033865799 |
| Zolamine | ZINC000000002257 |
| Zolasartan | ZINC000001552379 |
| Zolazepam | ZINC000000002259 |
| Zoledronic-acid | ZINC000003803652 |
| Zolenzepine | ZINC000022463356 |
| Zolertine | ZINC000005421643 |
| Zoliflodacin | ZINC000145806066 |
| Zolimidine | ZINC000000002261 |
| Zoliprofen | ZINC000000000851, ZINC000001846293 |
| Zolmitriptan | ZINC000000005298, ZINC000000015515 |

Table S1. (continued next page)

| Drug name | ZINC ID |
| --- | --- |
| Zoloperone | ZINC000036093059 |
| Zolpidem | ZINC000000003876 |
| Zomebazam | ZINC000000002262 |
| Zomepirac | ZINC000000057537 |
| Zometapine | ZINC000000002263 |
| Zonampanel | ZINC000001891930 |
| Zoniclezole | ZINC000000015778, ZINC000001851203 |
| Zoniporide | ZINC000003933046 |
| Zopiclone | ZINC000019632834, ZINC000019632839 |
| Zopolrestat | ZINC000000538557 |
| Zosuquidar | ZINC000100029945 |
| Zotepine | ZINC000000002264 |
| Zstk-474 | ZINC000100683572, ZINC000100683574 |
| Zucapsaicin | ZINC000004468952 |
| Zuclopenthixol_acetate | ZINC000030691716 |
